# Differential Proteomic Analysis of Human Placenta-Derived Mesenchymal Stem Cells Cultured on Normal Tissue Culture Surface and Hyaluronan-Coated Surface 

**DOI:** 10.1155/2016/2809192

**Published:** 2015-12-29

**Authors:** Tzyy Yue Wong, Ying-Hui Chen, Szu-Heng Liu, Mairim Alexandra Solis, Chen-Hsiang Yu, Chiung-Hsin Chang, Lynn L. H. Huang

**Affiliations:** ^1^Institute of Biotechnology, College of Bioscience and Biotechnology, National Cheng Kung University, Tainan 701, Taiwan; ^2^Department of Obstetrics and Gynecology, National Cheng Kung University, Tainan 701, Taiwan; ^3^Department of Biotechnology and Bioindustry Sciences, National Cheng Kung University, Tainan 701, Taiwan; ^4^Institute of Clinical Medicine, College of Medicine, National Cheng Kung University, Tainan 701, Taiwan; ^5^Research Center of Excellence in Regenerative Medicine, National Cheng Kung University, Tainan 701, Taiwan; ^6^Advanced Optoelectronic Technology Center, National Cheng Kung University, Tainan 701, Taiwan

## Abstract

Our previous results showed that hyaluronan (HA) preserved human placenta-derived mesenchymal stem cells (PDMSC) in a slow cell cycling mode similar to quiescence, the pristine state of stem cells *in vivo*, and HA was found to prevent murine adipose-derived mesenchymal stem cells from senescence. Here, stable isotope labeling by amino acid in cell culture (SILAC) proteomic profiling was used to evaluate the effects of HA on aging phenomenon in stem cells, comparing (1) old and young passage PDMSC cultured on normal tissue culture surface (TCS); (2) old passage on HA-coated surface (CHA) compared to TCS; (3) old and young passage on CHA. The results indicated that senescence-associated protein transgelin (TAGLN) was upregulated in old TCS. Protein CYR61, reportedly senescence-related, was downregulated in old CHA compared to old TCS. The SIRT1-interacting Nicotinamide phosphoribosyltransferase (NAMPT) increased by 2.23-fold in old CHA compared to old TCS, and is 0.48-fold lower in old TCS compared to young TCS. Results also indicated that components of endoplasmic reticulum associated degradation (ERAD) pathway were upregulated in old CHA compared to old TCS cells, potentially for overcoming stress to maintain cell function and suppress senescence. Our data points to pathways that may be targeted by HA to maintain stem cells youth.

## 1. Introduction

Senescence is when cells reach an irreversible growth arrest [1] and is known to play roles in various biological processes such as development, apoptosis, and aging [2]. It was reviewed that mitotic cells, cells undergoing proliferation, are prone to senescence [3]. Mitotic cells include stem cells, epithelial, vascular, and fibroblastic cells. The model for cellular senescence first originated from Hayflick and Moorhead, who reported in 1961 that normal human diploid cells had limited replicative lifespan [4]. Since then, senescence is believed to play a role in two potential circumstances, namely, (1) to prevent tumor growth and (2) as a normal way towards aging. The trigger of cellular aging includes many factors, such as oxidative stress [5], genomic instability [6], altered niche microenvironment [6], altered mitochondrial function [7, 8], altered epigenetic regulations [9], and stem cells exhaustion [9]. Previous study showed that genome stability such as maintained telomere length extended cell population doubling and reduced cellular aging in normal retinal pigment epithelial cells and foreskin fibroblasts [10]. Thus, the link between limited replicative senescence and actual aging phenomenon had been established.

The known aging mechanisms include telomere shortening [11], reactive oxygen species (ROS) accumulation, accumulated DNA damage-induced cell cycle regulation factors p53, p16, and p21 [3, 6], and altered cytoplasmic metabolism [12], all potentially leading to aging. The aging of stem cells leading to stem cell population decline had been suggested to occur in the human disease known as Hutchinson Gilford Progeria Syndrome [13]. The mesenchymal stem cells (MSC) from aged Sprague-Dawley rats were found to have compromised MSC function compared to that from young Sprague-Dawley rats [14]. The MSC are maintained* in vivo* as multipotent cells, capable of adipogenesis, osteogenesis, and chondrogenesis differentiation. Although it is well-known that stem cells are preserved in a pristine or youthful state compared to somatic cells* in vivo*, the mechanism involved is not fully understood. As a result, MSC are presented as a good model for studying the aging phenomenon.

The goal was to study aging phenomenon using stable isotope labeling by amino acid in cell culture (SILAC), a nonradioactive labeling method to label human placenta-derived MSC (PDMSC). This study aimed to confirm the previous results observed with hyaluronan (HA) presence. HA is a major component of extracellular matrix (ECM) that belongs to the glycosaminoglycan (GAG) family. It was observed that HA played a role in maintaining PDMSC in slow-cell cycling mode similar to stem cell quiescence [15]. HA induced multidrug resistance in PDMSC via CD44-PI3K-Akt pathway [16]. HA was found to prevent murine adipose-derived stromal cells from senescence [17]. PDMSC of older passage was compared to younger passage. It was found that PDMSC underwent replicative senescence after long-term* in vitro* expansion, in particular, the upregulation of proaging proteins and structural proteins. Molecules pertaining to normal cell function maintenance such as the endoplasmic reticulum-associated degradation (ERAD) pathway during stress and the COP9 signalosome-specific phosphorylation for p53 degradation were upregulated under HA presence compared to without HA, suggesting that the PDMSC under HA culture condition intended to suppress the stress of aging.

## 2. Materials and Methods

### 2.1. Cell Isolation

Full-term human placentas were obtained from mothers at the National Cheng Kung University Hospital with informed consent. Procedure of the human placenta handling and cell isolation was approved by the Institutional Review Board. PDMSC were isolated based on previous method [15]. In brief, the chorionic villi layer was harvested and rinsed in Hanks' Balanced Salt Solution (Sigma, St. Louis, MO, USA). The tissues were cut into tiny pieces and digested by 347 U/mL Collagenase Type 2 (Worthington Biochemical Corporation) at 37°C for 40 min. The digested tissues went through filters from 500 and 104 to 37 *μ*m. Percoll (Percoll; GE Healthcare, Uppsala, Sweden) was used for density gradient centrifugation to isolate mononuclear cells. The isolated cells were seeded at 3 × 10^4^ per cm^2^ in Dulbecco's modified Eagle's medium-low glucose, with 10% fetal bovine serum from Gibco BRL, Life Technologies, Grand Island, NY, USA, at 37°C with 5% CO_2_. Cells were cultured for 10–14 days until they reached a confluence of 70–80% and then passaged. The cells cultured on 30 *μ*g/cm^2^ HA–coated plates were termed CHA; those cultured on polystyrene tissue-culture surface were termed TCS.

### 2.2. Differentiation Potential Analysis

For adipogenic differentiation, induction medium contained final concentration of 1 *μ*M dimethyl sulfoxide (DMSO; Sigma, St. Louis, MO, USA), 0.2 mM indomethacin (Sigma, St. Louis, MO, USA), 0.5 mM 3-Isobutyl-1-methylxanthine (IBMX; Sigma, St. Louis, MO, USA), 10 *μ*M insulin, and 10% fetal bovine serum (FBS; GibcoBRL, Grand Island, NY, USA) in Dulbecco's modified Eagle's medium high glucose medium (DMEM; Gibco BRL, Grand Island, NY, USA). Cells were seeded at density 1 × 10^4^ per cm^2^ until 100% confluence was reached, and medium was changed to induction medium for 4 weeks. Induction medium was changed every 72 hours. Oil Red O (Sigma, St. Louis, MO, USA) was used to stain oil droplets. In brief, cells were fixed in 4% paraformaldehyde, washed with 1x phosphate buffer saline (PBS), rinsed in 60% isopropanol for three minutes, and stained in Oil Red O for 1 hour. After that, cells were washed using 60% isopropanol once, then rinsed in double-distilled water, and rinsed in 0.05% (w/v) sodium carbonate solution (Riedel-de Haën, Sigma, St. Louis, MO, USA) for two minutes. Finally, cells were counterstained with hematoxylin for one minute, washed with water, and analyzed under microscope. For chondrogenic differentiation, induction medium contained final concentration of 6.25 *μ*g/mL insulin, 50 nM ascorbic acid (JT Baker), and 10 ng/mL tumor growth factor-beta1 (TGF-*β*1; CellGS, St. Louis, MO, USA) in DMEM high glucose medium without FBS. Cells were seeded at density 1 × 10^4^ per cm^2^ until 100% confluence was reached, and medium was changed to induction medium for 4 weeks. Induction medium was changed every 72 hours. Accumulation of glycosaminoglycan was analyzed by staining with Alcian blue (Sigma, St. Louis, MO, USA). Briefly, cells were fixed in 4% paraformaldehyde, washed with PBS, and incubated in 1 N hydrogen chloride (HCl; Sigma, St. Louis, MO, USA) solution for five minutes. Next, cells were stained with 3% Alcian blue solution in 0.1 N HCl for 30 minutes. Finally, cells were washed with water and analyzed under microscope. Osteogenesis differentiation assay was performed using the induction medium supplemented with 10 *μ*M DMSO, 10 nM ascorbic acid, 10 mM 2-glycerophosphate (Sigma, St. Louis, MO, USA), and 10% FBS in DMEM high glucose medium. Seeding cell density was 1 × 10^4^ per cm^2^, and experiment was performed upon reaching 100% confluence. Calcification was visualized after staining with Alizarin Red S (Sigma, St. Louis, MO, USA). Cells were fixed in 4% paraformaldehyde, washed with PBS, and stained in Alizarin Red S solution for 20 minutes. Finally, cells were washed with water three times and analyzed microscopically.

### 2.3. Cumulative Population Doubling

To determine cumulative population doubling, cells were seeded at 0.7 × 10^4^ per cm^2^ for normal tissue-culture surface without HA (TCS), and 2.5 × 10^4^ per cm^2^ for HA-coated surface (CHA) on 24-well plate. Cells were subcultured when 80% confluence was reached, and cell number was counted. Population doubling was calculated using the equation: Logarithm of (final total cell number/initial cell number seeded) to base two. Finally, cumulative population doubling was derived by taking the sum of each population doubling.

### 2.4. SILAC Cell Culture

The cells, PDMSC, were cultured in arginine and lysine minus DMEM-low glucose (Sigma, St. Louis, MO, USA), with 10% dialyzed FBS (Thermo Fisher Scientific Incorporation, MA, USA) for 5-6 doublings, and tested for heavy-labeled incorporation efficiency before analysis of protein. The heavy amino acids ^13^C_6_ L-Arginine-HCl and ^13^C_6_ L-lysine-2HCl were supplemented at final concentration 0.1 mg/mL in DMEM-low glucose for heavy labeling, whereas L-arginine-HCl and L-lysine-2HCl were supplemented at final concentration 0.1 mg/mL in DMEM-low glucose for light labeling. All the amino acids for labeling were purchased from Thermo Fisher Scientific Incorporation, MA, USA. Incorporation of cells with either heavy or light amino acids commenced from passages two to five for young PDMSC, and from passages 14 to 17 for old PDMSC. The old TCS (OTCS^H^) and young CHA (YCHA^H^) were heavy-labeled; the young TCS (YTCS^L^) and old CHA (OCHA^L^) were light-labeled.

### 2.5. Hyaluronan-Coated Surface Preparation

Hyaluronan (Mw = 1470 kDa; Lifecore, MN, USA) was first dissolved in double-distilled water and then to working concentrations right before use. The hyaluronan was administered onto polystyrene surface and dried using hot plate for 30 min. The final concentration of hyaluronan was 30 *μ*g/cm^2^.

### 2.6. Sample Preparation

Method for sample collection was referred to previous [18] method with a few modifications. Reduction buffer containing 25 mM ammonium bicarbonate (ABC; Sigma, St. Louis, MO, USA), 2 mM dithiothreitol (DTT; JT Baker), and 8 M urea (Sigma, St. Louis, MO, USA) with complete protease inhibitor mixture (Roche Applied Science) was used to lyse cells on ice. A scraper was used to collect lysates and transferred to a new microcentrifuge tube. The lysates were left on ice for 30 min and vortexed intermittently in every 5 min. After that, lysates were centrifuged for 20 min at 18°C, 20,000 g. Supernatant was collected in new microcentrifuge tube, quantified using Bradford assay. The collected protein lysates were checked for integrity by 8% sodium dodecyl sulfate-polyacrylamide gel electrophoresis (SDS-PAGE) before further analysis. Protein concentration was measured using Bradford assay (Thermo Fisher Scientific Incorporation, MA, USA), and absorbance was measured at 595 nm. Protein of 10 *μ*g was added into 10 *μ*L of reduction buffer, kept at 37°C for 1 hr. Solution of 20 mM iodoacetamide (Sigma, St. Louis, MO, USA) prepared in 25 mM of ABC was added for sulphur-hydrogen groups alkylation, reacted at room temperature for 1 hr in the dark. Then, 20 mM DTT was used to quench the alkylation. The concentration of urea was brought down to less than 1 M with the 25 mM ABC. Lysates were digested in 0.1 *μ*g/*μ*L lysine-C (Wako Chemicals, Richmond, VA, USA) at 37°C for 2 hr. Followed by further 0.1 *μ*g/*μ*L trypsin (Promega, Madison, WI, USA) digestion at 30°C for 12 hr. The reaction was stopped by adding 1% of formic acid (Sigma, St. Louis, MO, USA). Each protein sample was loaded on C_18_ Ziptip (Millipore, Temecula, CA, USA) for purification. Finally, the mixture was desiccated using speed vacuum until dried before sending for LTQ-Orbitrap XL (Thermo Fisher Scientific Incorporation, MA, USA) LC-MS/MS at the Academia Sinica, Institute of Biological Chemistry. The protein lysates of OTCS^H^ were mixed with YTCS^L^, OCHA^L^ mixed with OTCS^H^, and OCHA^L^ mixed with YCHA^H^ in same cell number.

### 2.7. Incorporation Efficiency Analysis

Before sending experimental mixed samples for liquid chromatography-tandem mass spectrometry (LC-MS/MS) identification and quantitation, aliquots of the heavy-labeled protein lysates were first assessed for incorporation efficiency using LC-MS/MS for heavy amino acids-labeled peptides incorporation percentage. The parameters set for incorporation efficiency analysis were the same as in the mass spectrometry data analysis for the mixed experimental groups mentioned in the following section. The incorporation percentage was analyzed using DanteR (1.0.0.10 version) software in which parameters were set according to MaxQuant format. The incorporation percentage above 95% for heavy amino acids was required for further protein identification and quantitation.

### 2.8. Mass Spectrometry Data Analysis

Raw data obtained from LC-MS/MS was analyzed using MaxQuant (1.3.0.5 version, Max Planck Institute of Biochemistry), and proteins were identified based on the human FASTA file (downloaded in June 2013) from Uniprot. The raw data from LC-MS/MS was uploaded to MaxQuant, and MS/MS spectra were further searched using Andromeda search engine. For parameters, the number of missed cleavages allowed was two. For MS/MS spectrum and sequence parameters, Fourier transform mass spectrum (FTMS), ion-trap mass spectrum (ITMS), and time-of-flight (TOF) were set to 20 p.p.m, 0.5 Da, and 0.1 Da, respectively, whereas for the top peaks per 100 Da, FTMS, ITMS, and TOF were set to 10 p.p.m, 6 Da, and 10 Da, respectively. The raw data was searched with fixed modification carbamidomethyl. For identification and quantification parameters, peptide false discovery rate (FDR), site FDR, maximal peptide posterior error probability (PEP), minimum peptides, minimal razor plus unique peptides, and minimal unique peptides were set at 0.01, 0.01, 1, 1, 1, and zero, respectively. Finally, the protein FDR was set at 0.01%. To be considered SILAC pair, the identified peptides had mass difference of 6 Da. From MaxQuant analysis, the protein identity, the heavy to light ratios, and peptide intensities were shown in columns in the result. Protein abundance was derived from the peptide intensity in logarithm to base of 10 and the normalized heavy- and light-labeled protein ratios in logarithm to base of two. The normalized ratios ≧1.50 were discussed in this study.

### 2.9. Functional Analysis

To analyze protein functions, UniProtKB/Swiss-Prot was used, in combination with STRING9.1. Proteins with significance analyzed from Perseus software (1.3.0.4, Max Planck Institute of Biochemistry) for normalized ratios were selected from the experimental groups and searched using UniProtKB/Swiss-Prot. Proteins that lack previous study as reference were confirmed using STRING9.1 for protein-protein networks.

### 2.10. Statistical Analysis

For statistical analysis, Student's *t*-test was used to calculate *P* value. For proteomic analysis, the software Perseus was used to calculate significance *B* for *P* value. Proteins that deviate significantly from zero point (where heavy to light ratio equals zero) in the abundant proteins show the highest significance *B* value with the lowest *P* value where *P* values below 0.05 were identified as significant.

## 3. Results

### 3.1. Hyaluronan Influenced PDMSC Proliferation

**Figure 1:**
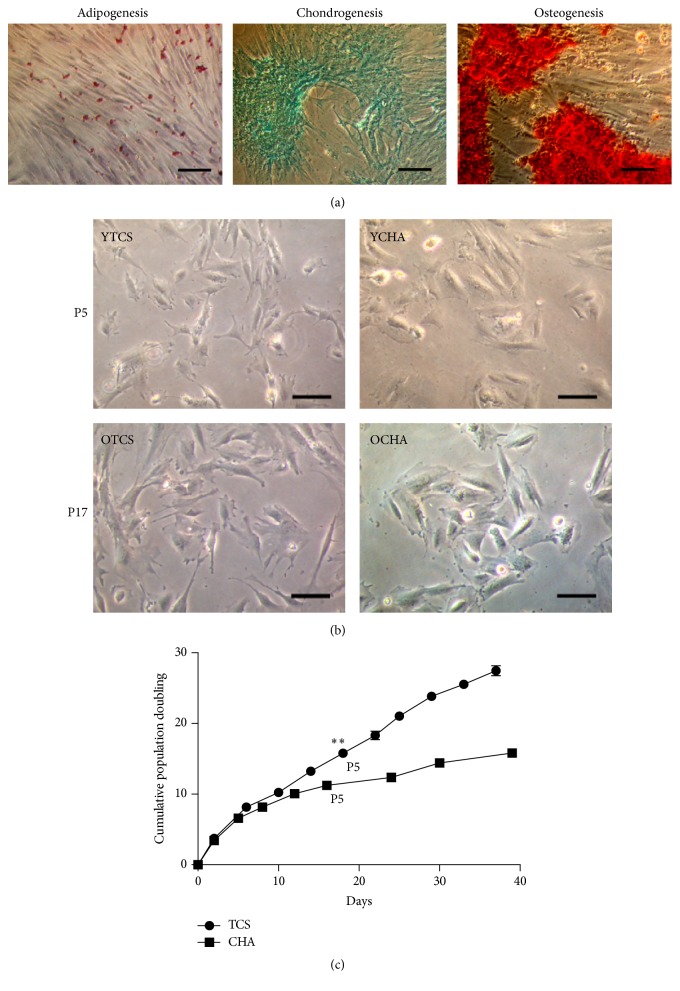
Hyaluronan-coated surface affected cell proliferation of PDMSC. (a) The PDMSC grown on tissue-culture polystyrene surface differentiated to adipocytes, chondrocytes, and osteocytes after induction for 4 weeks. Scale bar = 100 *μ*m. (b) The PDMSC presented fibroblastic morphology on tissue-culture polystyrene surface (TCS) and formed aggregate on tissue-culture polystyrene surface coated with 30 *μ*g/mL hyaluronan (CHA). The cells at P5 are noted as young and P17 as old. In CHA, the culture of PDMSC on HA-coated surface began at P2; thus, P2 + 3 passages (P5) and P2 + 15 passages (P17) for young and old CHA, respectively. Scale bar = 100 *μ*m. (c) The proliferation of PDMSC on TCS and CHA grown for a period of 5 weeks. Each of the data points represents cumulated population doubling for one passage. Three independent experiments were performed in TCS and CHA and data are represented in mean ± SD. ^*∗∗*^ *P* value <0.01.

**Figure 2:**
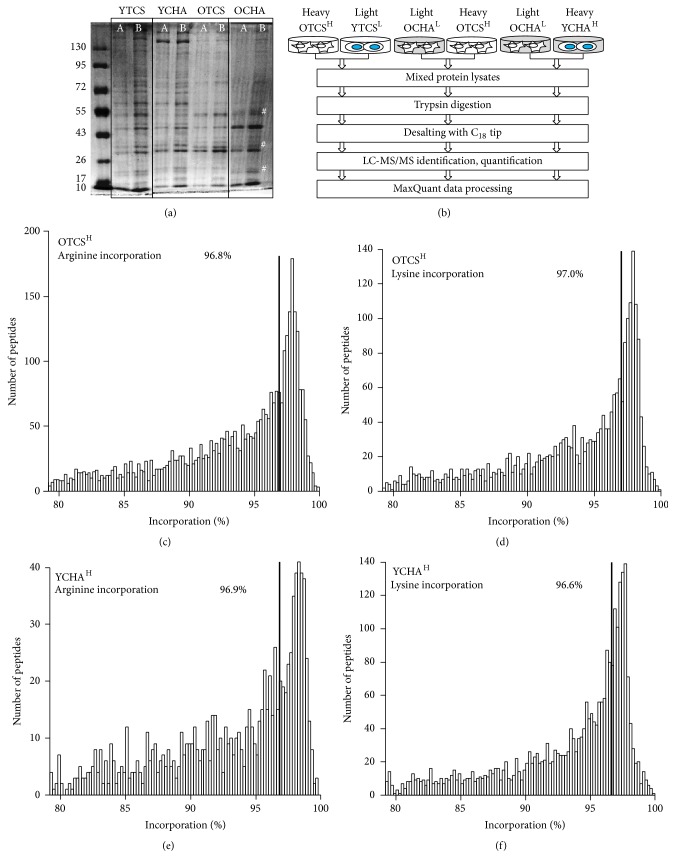
Proteomic workflow and incorporation rate of SILAC-labeled PDMSC. (a) The protein lysates collected at P5 and P17 for PDMSC cultured on TCS and CHA were separated on 8% SDS gel and stained with coomassie blue. The amounts of protein loaded were 10 *μ*g (Lane A) and 30 *μ*g (Lane B), respectively, for YTCS, YCHA, OTCS, and OCHA. The symbol (#) represents varied protein bands in the OCHA (Lane B) compared to OTCS (Lane B). (b) The illustration shows experimental workflow. (c-d) Heavy amino acids incorporation for OTCS^H^ after 5-6 doublings. (e-f) Heavy amino acids incorporation for YCHA^H^ after 5-6 doublings. Incorporation efficiency of the heavy arginine and lysine for PDMSC were determined before further protein identification and quantitation analysis using LC-MS/MS.

The PDMSC isolated from human placenta demonstrated adipogenic, chondrogenic, and osteogenic differentiation potential after a period of 4-week induction (Figure 1(a)). The morphology for PDMSC cultured on TCS was fibroblastic at passage 5 and was more spread at passage 17 (Figure 1(b)). In contrary, PDMSC cultured on CHA formed aggregate (Figure 1(b)). Proliferative profile for PDMSC cultured on TCS varied significantly from CHA (Figure 1(c)). To further investigate hyaluronan effect on PDMSC, protein lysates were collected and integrity was determined using SDS gel for YTCS, YCHA, OTCS, and OCHA (Figure 2(a)). Figures 2(c)–2(f) show the incorporation percentage for heavy arginine and lysine which were above 95% for OTCS and YCHA.

### 3.2. Different Patterns of Aging Phenomena with and without Hyaluronan

**Figure 3:**
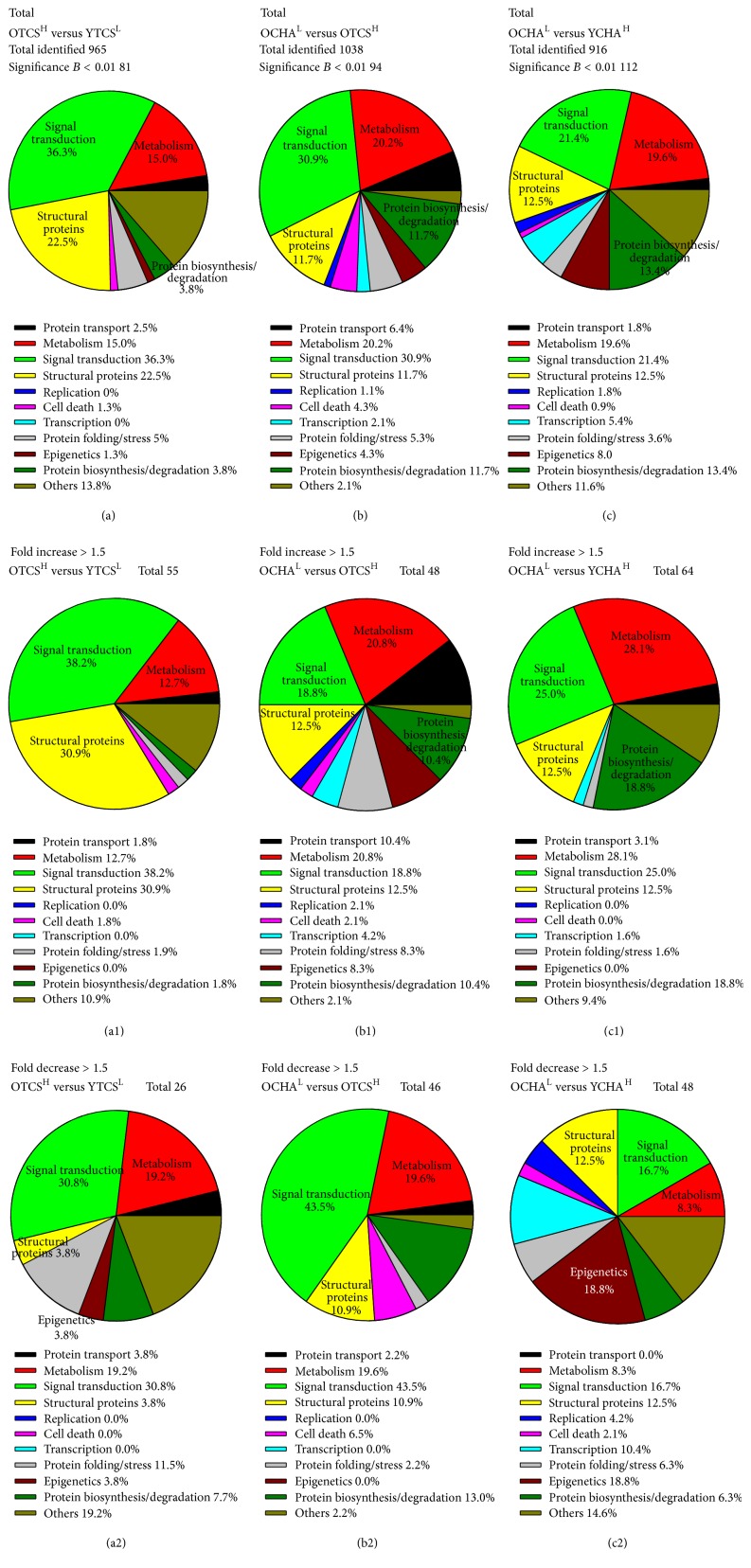
The aging phenomena in HA absence detected high percentages of signal transduction and structural proteins. Pie charts representing the total proteins identified with ratio above 1.5 in the (a) OTCS^H^ versus YTCS^L^, (b) OCHA^L^ versus OTCS^H^, and (c) OCHA^L^ versus YCHA^H^ groups, respectively. (a1) The OTCS^H^ had 0% of replication-related proteins, showing that replication had slowed down or ceased. (a2) The upregulated proteins in OTCS^H^ had 30.9% of structural proteins and 3.8% of downregulated proteins cell structural. Cell structure may have played a major role in the aging cells. (b1) In comparison to OTCS^H^, OCHA^L^ had 10.4% upregulated proteins of biosynthesis/degradation proteins, which correlated with the high percentage of metabolic proteins (20.8%), indicating that OCHA^L^ had higher metabolic activity. (b2) The group OCHA^L^ had 43.5% downregulated proteins for signal transduction. (c1) The metabolic activity (28.1%) in OCHA^L^ was higher compared to YCHA^H^, which may have correlated with the protein biosynthesis/degradation activity of 18.8%. The aging phenomena in the OCHA^H^ differed from that in OTCS^H^ in (a1), where the upregulated 12.7% of metabolic and 1.8% biosynthesis/degradation proteins. (c2) Epigenetics consisted 18.8% of the downregulated proteins in OCHA^L^, suggesting epigenetic regulation of cells after long-term HA presence.

Result showed that a total of 965, 1038, and 916 proteins were identified in the groups OTCS^H^ versus YTCS^L^, OCHA^L^ versus OTCS^H^, and OCHA^L^ versus YCHA^H^, respectively (Figures 3(a)–3(c)). The protein distribution in the aging phenomenon for OTCS^H^:YTCS^L^ differed from OCHA^L^:YCHA^H^ in that OCHA^L^:YCHA^H^ had 13.4% and 8.0% proteins for biosynthesis/degradation and epigenetics, respectively; OTCS^H^:YTCS^L^ had 3.8% and 1.3% proteins for biosynthesis/degradation and epigenetics, respectively (Figures 3(a) and 3(c)). Between OCHA^L^ and OTCS^H^, 11 out of 94 (11.7%) were structural, and 29 out of 94 (30.9%) were signal transduction proteins (Figures 3(a) and 3(b)). Interestingly, replication-related genes were not found to be modulated in the OTCS^H^:YTCS^L^ (Figure 3(a)).

**Figure 4:**
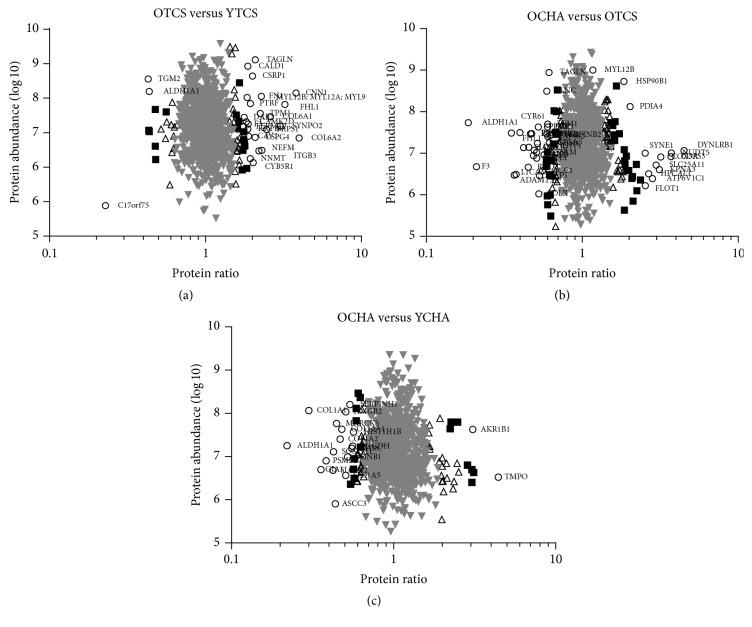
The normalized heavy- and light-labeled protein ratios plotted against total peptide intensities. The distribution of proteins identified is lower at higher abundance, confirming that protein quantification is more accurate. The data points are colored based on their significance *B* calculated using software Perseus where the gray and black triangles represent *P* value >0.05, squares *P* value <0.01, and circles *P* value <0.001. Abundant protein ratios for (a) OTCS^H^ versus YTCS^L^, (b) OCHA^L^ versus OTCS^H^, and (c) OCHA^L^ versus YCHA^H^.

The OCHA^L^:YCHA^H^ had 18 out of 64 (28.1%) compared to 7 out of 55 (12.7%) of upregulated metabolism-related proteins (Figures 3(a1) and 3(c1)). The OCHA^L^ maintained a high percentage of 20.8% metabolism-related proteins compared to OTCS^H^ (Figure 3(b1)). The OTCS^H^:YTCS^L^ had 38.2% for signal transduction and 30.9% for structural proteins compared to 25% signal transduction and 12.5% structural proteins in OCHA^L^:YCHA^H^ (Figures 3(a1) and 3(c1)). In contrary, 43.5% for signal transduction and 10.9% structural proteins were downregulated in OCHA^L^:OTCS^H^ (Figure 3(b2)). To represent the distribution cloud of abundant proteins identified, normalized heavy to light (H/L) ratios were plotted against peptide intensities for the groups OTCS^H^:YTCS^L^ (Figure 4(a)), OCHA^L^:OTCS^H^ (Figure 4(b)), and OCHA^L^:YCHA^H^ (Figure 4(c)). Each of the identified up- or down regulated proteins with significance *B* value between 0.01 and 0.001 was labeled with gene names (Figures 4(a)–4(c)).

### 3.3. Downregulation of Proaging Proteins in Old Passage Cells under Hyaluronan Presence

Tables 1 and 2 show the proteins with significance *B* value between 0.01 and 0.001 and this overlapped in the groups OTCS^H^:YTCS^L^, OCHA^L^:OTCS^H^ and OCHA^L^:YCHA^H^. The Lamin-B1 (LMNB1) was 0.52-fold less in OCHA^L^ compared to OTCS^H^. Previous study reported that nesprin-1 (SYNE1) maintained nuclear integrity and was 2.54-fold downregulated in OTCS^H^ compared to OCHA^L^. As for HMGA1, it was 1.61-fold downregulated in OTCS^H^ versus OCHA^L^. In consistency, the senescence-related protein transgelin (TAGLN) was 0.61-fold less in OCHA^L^ versus OTCS^H^, and 2.1-fold higher in OTCS^H^ versus YTCS. The CCN family protein, cysteine rich protein 61 (CYR61), was 0.35-fold less in OCHA^L^ compared to OTCS^H^. Furthermore, the probable fructose-2,6-bisphosphatase TIGAR (TIGAR) decrease by 0.59-fold in OTCS^H^ compared to YTCS^L^ (Table 1).

The Rho-related GTP-binding protein RhoE (RND3) had 1.59-fold increase in OTCS^H^ compared to YTCS^L^ and 0.45-fold decrease in OCHA^L^ compared to OTCS^H^ (Table 1). Consistent with previous report that Lin11-Isl-1-Mec-3 or LIM domain was activated in case of RND3 upregulation [32], the actin-binding domain protein 1 (LIMA1) and four and a half LIM domains protein 1 (FHL1) had 1.52- and 3.23-fold increase in OTCS^H^ versus YTCS^L^, respectively (Table 1). Consistently, LIMA1 and FHL1 were 0.45- and 0.40-fold lower in OCHA^L^ compared to OTCS^H^, respectively. Furthermore, tropomyosin alpha-1 (TPM1) for cell contraction [36] was 0.6-fold lower in OCHA^L^ versus OTCS^H^ and 2.25-fold higher in OTCS^H^ versus YTCS^L^ (Table 1). Other actin-binding proteins were neurofilament medium polypeptide (NEFM) and filamin-C (FLNC). The OCHA^L^ had 0.6-fold of NEFM and 0.59-fold of FLNC decrease versus OTCS^H^. In addition, OTCS^H^ had 2.3-fold of NEFM and 1.65-fold of FLNC increase versus YTCS^L^ (Table 1).

### 3.4. Activation of Endoplasmic Reticulum-Associated Degradation Pathway Molecules in Hyaluronan Presence

The endoplasmic reticulum (ER) stress factors, protein disulfide-isomerase A4 (PDIA4), endoplasmin (HSP90B1), hypoxia upregulated protein 1 (HYOU1), and 78 kDa glucose-regulated protein (HSPA5), were 2.02-, 1.85-, 1.73-, and 1.66-fold higher, respectively, in OCHA^L^ compared to OTCS^H^ (Table 2). The Ras-related protein RRAS2 was 0.6-fold lower in OCHA^L^ compared to OTCS^H^ (Table 2). And the protein degradation COP9 signalosome complex components COPS2 and COPS3 were 1.76- and 3.71-fold higher in OCHA^L^ compared to OTCS^H^ (Table 2). Additionally, the ubiquitin/proteasomal system proteins ubiquitin carboxyl-terminal hydroxylase 5 (USP5) was 1.52-fold higher in OCHA^L^ versus OTCS^H^ (Table 2). Consistent with previous reports on the environmental stress-related protein dual specificity mitogen-activated protein kinase kinase 3, dual specificity mitogen-activated protein kinase kinase 6 (MAP2K3; MAP2K6) was 0.62-fold lower in OCHA^L^ compared to OTCS^H^ (Table 2). And the Nicotinamide phosphoribosyltransferase (NAMPT), a major regulator of SIRT1, was 2.23-fold higher in OCHA^L^ compared to OTCS^H^ and 0.48-fold lower in OTCS^H^ compared to YTCS^L^ (Table 2).

### 3.5. Potential p53-Mediated Senescence Suppression Detected under Hyaluronan Culture Condition

**Figure 5:**
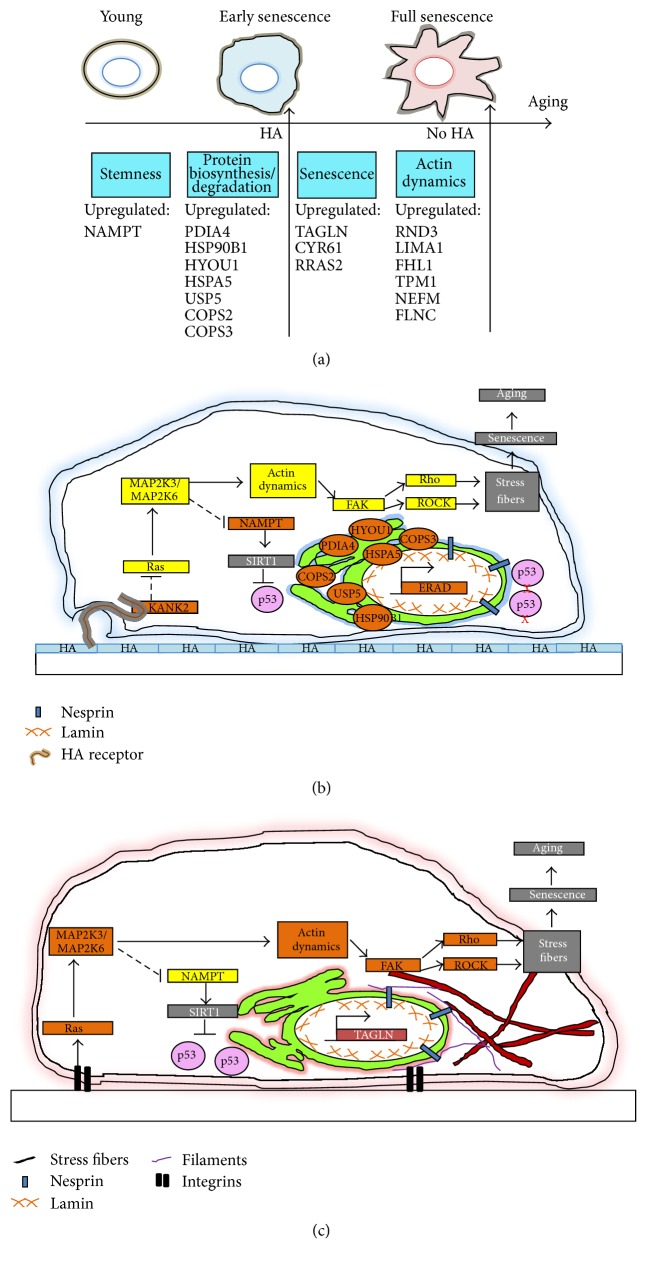
PDMSC underwent normal aging process without HA presence. (a) The aging phenomenon of PDMSC was hypothesized to be attenuated under HA presence. (b) HA was hypothesized to bind to its receptor with ankyrin repeat domain (KANK2) and suppressed the downstream actin-interacting proteins. (c) In absence of HA, the Ras signaling was hypothesized to have increased over long period of expansion and increased the downstream actin dynamics, focal adhesion kinase (FAK), stress fiber formation, and enhanced aging. Yellow boxes represented downregulation; orange boxes represented upregulation.

The hypothesized pathway of the detected aging-related molecules is depicted in Figure 5. In absence of HA, the upregulation of Ras signaling, MAP2K3; MAP2K6, and actin-interacting proteins (RND3, LIMA1, FHL1, NEFM, TPM1, and FLNC) may have led to potential stress fiber formation, promoting senescence and aging (Figure 5(c)). However, HA presence led to the downregulation of Ras signaling, MAP2K3; MAP2K6, actin-interacting proteins (RND3, LIMA1, FHL1, NEFM, TPM1, and FLNC), upregulation of NAMPT, and upregulation of ER stress chaperone molecules which is the endoplasmic reticulum-associated degradation (ERAD) pathway. The upregulated NAMPT may have prevented p53-mediated senescence through p53 degradation and thus maintained the normal phenotype of the stem cells.

## 4. Discussion

In general, MSC are maintained in quiescence state when not differentiated. However, the MSC eventually lose their stem cell functions as cells are passaged for a prolonged period of time. As the number of senescent cells is accumulated due to environmental stress upon* in vitro* culture, the cells are prone to aging.

Structural proteins play important role in cell motility, cell adhesion, and cell shape which include nuclear structure maintenance. The structural protein consisted of intermediate filament, microtubules, and microfilaments. It is known that nuclear structure integrity is important for stem cell maintenance. The lamin-B1 (LMNB1) maintained nuclear structure via interacting with cytoskeleton, and its downregulation may lead to full senescence. LMNB1 protein levels declined in senescent human dermal fibroblasts and keratinocytes [19]. The spectrin repeat containing nuclear envelope 1 (SYNE-1) is required for maintenance of nuclear organization and structural integrity. According to STRING9.1 analysis, LMNB1 interacts with SYNE-1, and SYNE-1 links the nuclei to cytoskeletons via interacting with the nuclear envelope and F-actin.

The transgelin (TAGLN) was reported to interact with actin [20] to regulate the actin dynamics and stress fibers during senescence. In case of oxidative stress, the actin interaction with filaments was reported to be increased accompanied by cell apoptosis in yeast [21]. In yeast, loss of the gene encoding the actin-bundling protein Scp1p, which is homologue of mammalian SM22/transgelin (TAGLN), increased sensitivity to apoptosis during oxidative stress [21]. The cysteine-rich angiogenic inducer 61 CYR61, a CCN family protein that interacts with integrin, activates Rac1 and pRb, which led to senescence [24]. The CCN family protein includes Cyr61 (cysteine rich protein 61), CTGF (Connective Tissue Growth Factor), and NOV (nephroblastoma overexpressed gene). The CYR61 interacted with integrin *α*V*β*3 to support cell survival [25]; however, it promoted apoptosis in fibroblasts via interacting with *α*6*β*1 integrin [26].

The GTP-binding protein family is composed of Rho, Ras, Rab, Sar1/Arf, and Ran [48]. The Rho protein was reported to carry out three interactions in cells to form the stress fibers: (1) interacting with Rho-kinase (ROCK) and downstream LIM kinase to increase actin polymerization, (2) interacting with ROCK to stabilize actin, and (3) interacting with diaphanous (Dia) to assemble actin-myosin [32]. During senescence, actin filaments play important role in the cell structure [21]. Senescent human diploid fibroblasts had increased actin stress fibers, focal adhesion protein rearrangement, de novo protein synthesis, and enlarged cell size [49]. The RND3 is a Rho protein known to regulate actin polymerization [32] which is involved in actin-cytoskeleton dynamics [48]. With regard to stress fibers and Rho expression, the increase of RND3 and other structural proteins including LIMA1, FHL1, PDLIM1, TPM1, FLNC, and NEFM indicated that PDMSC may have undergone stress fiber formation towards senescence; however, it was inhibited under HA treatment condition.

Furthermore, the GTP protein Ras is induced during senescence, with upregulation of the cell cycle arrest factor p16 [50]. Ras signaling is important for stress response [20] and normal actin remodeling [20]. The Ras signaling is regulated by adenylate cyclase (Cyr1) to produce cAMP, which activated cAMP-dependent protein kinase A (PKA), leading to expression of stress response genes in normal cells [20, 37]. However, continued Ras-cAMP signaling led to abnormal actin remodeling and apoptosis during nutritional stress [20]. Previously, overexpression of Ras-cAMP inhibitor phosphodiesterase-2 (PDE2) in yeast reduced reaction oxygen species, and apoptosis, suggesting that Ras-cAMP may be induced by oxidative stress [51]. Another Ras signaling, RAS2, was found to regulate cell polarity in yeast through TPM1 and actin during temperature stress response [37].

The microenvironment confers cells to various types of environmental stresses. Whether the path to aging is due to intracellular factors or extracellular factors requires further studies. Other than nutritional stress, oxidative stress, and temperature stress mentioned above, endoplasmic reticulum stress (ER stress) was reported to be age-related [41] and is associated with upregulation of chaperones, induction of ERAD pathway, and attenuated protein translation [52]. The ER stress-responsive chaperone components include heat shock proteins [53], calnexin, calreticulin, and protein disulfide isomerases [41, 42]. Under environmental stress, normal cells utilize the ER stress components to protect from aggregation of misfolded proteins in order to maintain normal functions [41, 52]. Previous study indicated that heat shock protein 78 kDa glucose-regulated protein (HSPA5) is a biomarker for ER stress [53] which maintained integrity of neuronal cells [54]. However, the ER stress responsive proteins were indicated to be compromised in aged cells [52, 55]. Previous study indicated that decrease in ERAD factors shortened lifespan of* C. elegans* [56]. In consistency, the ER stress molecules (HSP90B1, HSPA5, PDI4, and HYOU1) were upregulated in OCHA^L^ compared to OTCS^H^ (Table 2).

Apart from ER stress chaperones, misfolded proteins are also processed through the ubiquitin/proteasomal system (UPS). During senescence, p53 is stabilized due to DNA damage response [3], and misfolded proteins are aggregated [57]. The ER stress responsive chaperones tried to prevent secretion of misfolded proteins and stabilization of p53 [57, 58]. It was reported that young cells possess normal protein degradation system that prevent damaged protein aggregation; however, this protein degradation system was compromised in old cells [59, 60]. It was reported that p53 targeted and downregulated TIGAR during stress responses such as DNA damage and activation of oncogene [27], which was consistent with our data that TIGAR was decreased in OTCS^H^ versus YTCS^L^ (Table 2). However, the role of p53 is complicated such that it regulates not only DNA damage response, but also energy metabolism glycolysis and oxidative stress [61]. Previous study indicated that p53 induced TIGAR expression to reduce glycolysis [62]. It was stated that p53 promoted cell survival and tumor growth; however, HA receptor CD44 was suppressed by p53 since HA had counter effect on p53 [63]. In this case, the role of HA may have been to overcome the proaging stress inducible by p53. Similarly, the COPS2, COPS3, and USP5 which may degrade p53 were significantly upregulated in OCHA^L^ compared to OTCS^H^ (Table 2).

HA is produced on the cell surface by HA synthases HAS1, HAS2, and HAS3 located in plasma membrane. The role of HA varies from normal stem cells to cancer cells. HA is known to play a role in wound repair without scar in fetal tissue [64]. Previous study indicated that endogenous HA regulated differentiation of embryonic stem cells [65]. Another previous study showed that HA-coated surface enhanced chondrogenesis [66]. Normal cells produced HA and secreted into the extracellular matrix; however, they altered structure of HA activated ERK1/2 to promote senescence [67]. In contrary, HA given in the form of hydrogel was shown to maintain self-renewal of embryonic stem cells [68]. Apart from this, HA presence provided stem cells with a hypoxic microenvironment upon cell culture for embryonic stem cells [69]. Although HA is a major component of extracellular matrix, the evidence of how HA is associated with stem cells maintenance is still lacking. According to study, stem cells reside in niche with hypoxic conditions [69, 70]. For spermatozoa and oocytes, HA was reported to maintain genome integrity [69], suggesting that HA confers stem cells functional maintenance. Based on our findings, HA may have downregulated the proaging protein TAGLN and the actin-interacting proteins RND3, LIMA1, FHL1, PDLIM1, TPM1, FLNC, and NEFM to maintain youthful cell structure. In addition, HA may have maintained normal PDMSC phenotype with the increase in ER stress factors to attenuate aging.

## Supplementary Material

Three Tables showing the full identified proteins are available as supplementary materials: (1) Table S1. Total proteins identified for OTCS^H^:YTCS^L^. (2) Table S2. Total proteins identified for OCHA^L^:OTCS^H^. (3) Table S3. Total proteins identified for OCHA^L^:YCHA^H^.

## Figures and Tables

**Table 1:** Structural maintenance and aging-related proteins. H, heavy; L, light.

Category	Protein IDs	Protein names	Gene name	Normalized ratio			Function	Reference
				H/L SC^b^ [%]	H/L^a^ SC^b^ [%]	H/L^a^ SC^b^ [%]		
				OTCS^H^:YTCS^L^	OCHA^L^:OTCS^H^	OCHA^L^:YCHA^H^		
Structural	P20700	Lamin-B1	LMNB1	0.94787 (8.4)	1.1111 (8.9)	0.5203 (7.3)	Nuclear structure	[19]

Structural	Q8NF91	Nesprin-1	SYNE1	NA^c^	2.5356 (0.4)	NA^c^	Nuclear maintenance	STRING^e^

Signal transduction	Q01995	Transgelin	TAGLN	2.0888 (65.2)	0.6132 (69.2)	1.0023 (51.7)	Replicative senescence	[20, 21]

Protein biosynthesis/degradation	P17096	High mobility group protein HMG-I/HMG-Y	HMGA1	0.86138 (23.4)	1.6148 (23.4)	1.063 (23.4)	Senescence, stemness	[22, 23]

Cell death	O00622	Protein CYR61	CYR61	1.0073 (22)	0.3514 (35.7)	NA^c^	Senescence, survival	[24–26]

Protein folding/stress	Q9NQ88	Probable fructose-2,6-bisphosphatase TIGAR	TIGAR	0.59062 (13)	1.149 (13)	1.42 (13)	Senescence, stress-responsive	[27, 28]

Structural	Q13459	Unconventional myosin-IXb	MYO9B	0.4769 (1.3)	N/A	2.1214 (1.1)	Cytoskeletons. Rho-mediated signaling	[29, 30]

Structural	O95747	Serine/threonine- protein kinase OSR1	OXSR1	ND^d^	ND^d^	1.6339 (6.5)	Cytoskeletons. Stress- responsive, regulates actin	[31]

Signal transduction	P61587	Rho-related GTP-binding protein RhoE	RND3	1.5923 (10.2)	0.4502 (17.2)	ND^d^	Cytoskeletons. Stress fiber formation, cell motility, and adhesion	[32–34]

Structural	Q9UHB6	LIM domain and actin-binding protein 1	LIMA1	1.5189 (15.3)	0.4547 (13.0)	0.6461 (7.6)	Cytoskeletons	[32]

Signal transduction	Q13642	Four and a half LIM domains protein 1	FHL1	3.2279 (24.1)	0.3981 (16.1)	1.2503 (12.7)	Cytoskeletons. Cardiac cell differentiation	[32]

Structural	O00151	PDZ and LIM domain protein 1	PDLIM1	1.5045 (37.1)	1.2779 (37.7)	1.4622 (27.4)	Cytoskeletons	[32]

Structural	P07197	Neurofilament medium polypeptide	NEFM	2.2981 (5.1)	0.4864 (9.4)	ND^d^	Cytoskeletons. Protein transport in neuron cells, axonal growth.	[35]

Structural	P09493	Tropomyosin alpha-1 chain	TPM1	2.2465 (35.2)	0.6003 (26.1)	NA^c^	Cytoskeletons. Cardiac muscle contraction	[36]

Structural	Q14315	Filamin-C	FLNC	1.6548 (19.6)	0.5924 (23.4)	1.2207 (15.3)	Cytoskeletons. Interacts with actin	STRING^e^

^a^Normalized ratio H/L reversed: 1/[H/L].

^b^Sequence coverage %.

^c^Not applicable.

^d^Not detected.

^e^Analyzed by STRING9.1.

**Table 2:** Stress-responsive, intracellular signaling proteins. H, heavy; L, light.

Category	Protein IDs	Protein names	Gene name	Normalized ratios			Function	Reference
				H/L SC^b^ [%]	H/L^a^ SC^b^ [%]	H/L^a^ SC^b^ [%]		
				OTCS^H^:YTCS^L^	OCHA^L^:OTCS^H^	OCHA^L^:YCHA^H^		
Signal transduction	P62070; P10301	Ras-related protein R-Ras2; Ras-related protein R-Ras	RRAS2; RRAS	1.4273 (11.5)	0.6302 (11.3)	NA^c^	Intracellular signaling	[37, 38]

Protein folding/stress	P46734; P52564	Dual specificity mitogen-activated protein kinase kinase 3; dual specificity mitogen-activated protein kinase kinase 6	MAP2K3; MAP2K6	1.2744 (9.2)	0.6174 (9.2)	ND^d^	Environmental stress	[39] STRING^e^

Metabolism	P43490	Nicotinamide phosphoribosyltransferase	NAMPT	0.47879 (13.8)	2.2273 (22.4)	1.3492 (27.1)	Interaction with SIRT1	[40]

Protein folding/stress	P13667	Protein disulfide-isomerase A4	PDIA4	0.83829 (22.3)	2.0283 (27.1)	1.46726 (29.1)	Protein folding	[41, 42]

Protein folding/stress	P14625; Q58FF3	Endoplasmin	HSP90B1	0.98611 (33.4)	1.8484 (33.3)	1.4263 (30.0)	Protein degradation	[41]

Protein folding/stress	Q9Y4L1	Hypoxia upregulated protein 1	HYOU1	0.894 (15.8)	1.73295 (14.6)	1.2425 (19.8)	Protein folding	[41]

Protein folding/stress	P11021	78 kDa glucose-regulated protein	HSPA5	0.873 (28.9)	1.6583 (27.2)	1.2862 (31.5)	Protein folding	[41, 42]

Signal transduction	P63098; Q96LZ3	Calcineurin subunit B type 1	PPP3R1	NA^c^	1.6969 (34.7)	ND^d^	Ca^2+^ regulation	STRING^e^

Signal transduction	P37235; P84074; P61601	Hippocalcin-like protein 1; neuron-specific calcium-binding protein hippocalcin	HPCAL1; HPCA	NA^c^	2.6682 (10.4)	2.5190 (18.1)	Ca^2+^ regulation	STRING^e^

Signal transduction	Q6UXH1	Cysteine-rich with EGF-like domain protein 2	CRELD2	NA^c^	1.7720 (8.2)	1.5329 (8.2)	Ca^2+^ regulation	STRING^e^

Protein biosynthesis/degradation	P45974	Ubiquitin carboxyl-terminal hydrolase 5	USP5	1.042 (6.5)	1.5207 (8.0)	2.1350 (4.4)	p53 degradation	[43]

Protein biosynthesis/degradation	P61201	COP9 signalosome complex subunit 2	COPS2	NA^c^	1.7612 (6.5)	1.2704 (8.6)	p53 degradation	[44, 45]

Protein biosynthesis/degradation	Q9UNS2	COP9 signalosome complex subunit 3	COPS3	NA^c^	3.7121 (13.5)	NA^c^	p53 degradation	[45]

Signal transduction	Q63ZY3	KN motif and ankyrin repeat domain-containing protein 2	KANK2	ND^d^	2.0810 (3.1)	0.801 (4.5)	Binding of ankyrin domain to CD44	[46, 47]

^a^Normalized ratio H/L reversed: 1/[H/L].

^b^Sequence coverage %.

^c^Not applicable.

^d^Not detected.

^e^Analyzed by STRING9.1.
