# Supplementary material for: Differential Proteomic Analysis of Human Placenta-Derived Mesenchymal Stem Cells Cultured on Normal Tissue Culture Surface and Hyaluronan-Coated Surface
Source: Stem Cells Int. 2015 Dec 29;2016:2809192. doi: 10.1155/2016/2809192 (PMC4709773; doi:10.1155/2016/2809192)
Supplement: Supplementary file 1 — Three Tables showing the full identified proteins are available as supplementary materials: (1) Table S1. Total proteins identified for OTCSH:YTCSL. (2) Table S2. Total proteins identified for OCHAL:OTCSH. (3) Table S3. Total proteins identified for OCHAL:YCHAH. [file 2809192.f1.pdf]

Supplementary Table S1

Total proteins identified for OTCS:YTCS

Protein IDs	Protein names	Gene names	Sequence coverage [%]	Ratio H/L OTCS^{H}: YTCS^{L}
Q9HAS0	Protein Njmu-R1	C17orf75	6.1	0.22831
P21980	Protein-glutamine gamma-glutamyltransferase 2	TGM2	25.6	0.42949
Q9UJU6	Drebrin-like protein	DBNL	11.2	0.43223
P00352	Retinal dehydrogenase 1	ALDH1A1	33.5	0.43593
P36542	ATP synthase subunit gamma, mitochondrial	ATP5C1	7.4	0.43611
P61916	Epididymal secretory protein E1	NPC2	38.4	0.47639
Q13459	Unconventional myosin-IXb	MYO9B	1.3	0.47691
P43490	Nicotinamide phosphoribosyltransferase	NAMPT	13.8	0.47879
Q14914	Prostaglandin reductase 1	PTGR1	25.5	0.51905
P53634	Dipeptidyl peptidase 1;Dipeptidyl peptidase 1 exclusion domain chain;Dipeptidyl peptidase 1 heavy chain;Dipeptidyl peptidase 1 light chain	CTSC	14	0.55378
P06454	Prothymosin alpha;Thymosin alpha-1	PTMA	26.1	0.56063
P20618	Proteasome subunit beta type-1	PSMB1	19.9	0.5644
Q9NQ88	Probable fructose-2,6-bisphosphatase TIGAR	TIGAR	13	0.59062
Q96C90	Protein phosphatase 1 regulatory subunit 14B	PPP1R14B	30.6	0.60349
Q92973;O14787	Transportin-1	TNPO1	6.3	0.60905
Q92598;O95757	Heat shock protein 105 kDa	HSPH1	24.7	0.61323
P28482	Mitogen-activated protein kinase 1	MAPK1	6.9	0.62381
Q9BS26	Endoplasmic reticulum resident protein 44	ERP44	10.6	0.62547
Q9UKK9	ADP-sugar pyrophosphatase	NUDT5	12.3	0.63161
P14735	Insulin-degrading enzyme	IDE	2.4	0.63259
P39687;O43423;O95626	Acidic leucine-rich nuclear phosphoprotein 32 family member A;Acidic leucine-rich nuclear phosphoprotein 32 family member C;Acidic leucine-rich nuclear phosphoprotein 32 family member D	ANP32A;ANP32C;ANP32D	8.8	0.63612
P50281	Matrix metalloproteinase-14	MMP14	7.2	0.63877
P02794	Ferritin heavy chain	FTH1	14.2	0.65603
Q16576;Q09028	Histone-binding protein RBBP7;Histone-binding protein RBBP4	RBBP7;RBBP4	11.3	0.65678

Q2TB90	Putative hexokinase HKDC1	HKDC1	2.9	0.6636
P15586	N-acetylglucosamine-6-sulfatase	GNS	7.2	0.66644
P34897;P34896	Serine hydroxymethyltransferase, mitochondrial	SHMT2	11.1	0.6753
Q16658	Fascin	FSCN1	19.5	0.67718
Q969H8	UPF0556 protein C19orf10	C19orf10	20.2	0.6796
P00491	Purine nucleoside phosphorylase	PNP	5.9	0.68025
P61604	10 kDa heat shock protein, mitochondrial	HSPE1	35.3	0.68156
P43487	Ran-specific GTPase-activating protein	RANBP1	29.9	0.68262
Q02790	Peptidyl-prolyl cis-trans isomerase FKBP4;Peptidyl-prolyl cis-trans isomerase FKBP4, N-terminally processed	FKBP4	29.2	0.68387
P15531	Nucleoside diphosphate kinase A	NME1	33.6	0.69364
P08621	U1 small nuclear ribonucleoprotein 70 kDa	SNRNP70	7.8	0.69518
Q15393	Splicing factor 3B subunit 3	SF3B3	3.4	0.69673
O75608	Acyl-protein thioesterase 1	LYPLA1	10.9	0.69882
P62328	Thymosin beta-4;Hematopoietic system regulatory peptide	TMSB4X	61.4	0.69989
P45973	Chromobox protein homolog 5	CBX5	12.6	0.70025
Q9BTT0	Acidic leucine-rich nuclear phosphoprotein 32 family member E	ANP32E	19	0.70221
P16035	Metalloproteinase inhibitor 2	TIMP2	15.9	0.70559
Q9Y617	Phosphoserine aminotransferase	PSAT1	21.4	0.7109
P10599	Thioredoxin	TXN	66.7	0.71296
P31948	Stress-induced-phosphoprotein 1	STIP1	21	0.7141
P09496	Clathrin light chain A	CLTA	6.9	0.71453
P49458	Signal recognition particle 9 kDa protein	SRP9	31.4	0.71491
P21266	Glutathione S-transferase Mu 3	GSTM3	22.7	0.71568
Q9H1E3	Nuclear ubiquitous casein and cyclin-dependent kinase substrate 1	NUCKS1	10.7	0.71729
P46926;Q8TDQ7	Glucosamine-6-phosphate isomerase 1;Glucosamine-6-phosphate isomerase 2	GNPDA1;GNPDA2	6.2	0.71759
P12004	Proliferating cell nuclear antigen	PCNA	29.5	0.71769
P62633	Cellular nucleic acid-binding protein	CNBP	14.1	0.71775
P38159;Q96E39;Q8N7X1;O75526	RNA-binding motif protein, X chromosome;RNA-binding motif protein, X chromosome, N-terminally processed;RNA binding motif protein, X-linked-like-1;RNA binding motif	RBMX;RBMXL1	9.5	0.71873

	protein, X-linked-like-1, N-terminally processed			
Q99714	3-hydroxyacyl-CoA dehydrogenase type-2	HSD17B10	16.5	0.71894
P11413	Glucose-6-phosphate 1-dehydrogenase	G6PD	8.2	0.71919
O95881	Thioredoxin domain-containing protein 12	TXNDC12	30.8	0.72135
P61421	V-type proton ATPase subunit d 1	ATP6V0D1	6.8	0.72345
P00492	Hypoxanthine-guanine phosphoribosyltransferase	HPRT1	22.5	0.72373
P09417	Dihydropteridine reductase	QDPR	35.7	0.72392
P10809	60 kDa heat shock protein, mitochondrial	HSPD1	37.5	0.73079
Q9HC38	Glyoxalase domain-containing protein 4	GLOD4	9.6	0.73174
P18084	Integrin beta-5	ITGB5	4.8	0.73432
P05120	Plasminogen activator inhibitor 2	SERPINB2	21	0.73452
P53597	Succinyl-CoA ligase [ADP/GDP-forming] subunit alpha, mitochondrial	SUCLG1	6.9	0.73614
Q13162	Peroxiredoxin-4	PRDX4	18.1	0.73694
P52815	39S ribosomal protein L12, mitochondrial	MRPL12	23.2	0.73714
Q9H488	GDP-fucose protein O-fucosyltransferase 1	POFUT1	6.7	0.73849
Q96RS6	NudC domain-containing protein 1	NUDCD1	6.3	0.74089
P06744;CON__Q3ZBD7	Glucose-6-phosphate isomerase	GPI	23.1	0.74139
P10412;P16402;Q02539;P22492	Histone H1.4;Histone H1.3	HIST1H1E;HIST1H1D	17.8	0.74359
Q01081;Q8WU68	Splicing factor U2AF 35 kDa subunit;Splicing factor U2AF 26 kDa subunit	U2AF1;U2AF1L4	14.2	0.74574
P61970	Nuclear transport factor 2	NUTF2	45.7	0.74593
P32119	Peroxiredoxin-2	PRDX2	47.5	0.74664
Q9P1F3	Costars family protein ABRACL	ABRACL	16	0.75053
O43396	Thioredoxin-like protein 1	TXNL1	19	0.75206
Q9UMS4	Pre-mRNA-processing factor 19	PRPF19	18.5	0.75251
O75531	Barrier-to-autointegration factor	BANF1	43.8	0.75311
P08107	Heat shock 70 kDa protein 1A/1B	HSPA1A	18.6	0.75374
Q9NUU7;Q9UHL0	ATP-dependent RNA helicase DDX19A;ATP-dependent RNA helicase DDX25	DDX19A;DDX25	7.5	0.75513
P26639	Threonine--tRNA ligase, cytoplasmic	TARS	22	0.75701
O43175	D-3-phosphoglycerate dehydrogenase	PHGDH	20.6	0.75953
P30040	Endoplasmic reticulum resident protein 29	ERP29	21.8	0.75981

P07305	Histone H1.0	H1F0	11.9	0.76062
Q9UJZ1	Stomatin-like protein 2	STOML2	12.9	0.76142
Q07021	Complement component 1 Q subcomponent-binding protein, mitochondrial	C1QBP	9.2	0.76221
P38646	Stress-70 protein, mitochondrial	HSPA9	22.7	0.76232
P50454	Serpin H1	SERPINH1	29.7	0.76374
Q9NR45	Sialic acid synthase	NANS	10.6	0.76406
P34932	Heat shock 70 kDa protein 4	HSPA4	21.8	0.76591
Q15185	Prostaglandin E synthase 3	PTGES3	25	0.76618
P61981	14-3-3 protein gamma;14-3-3 protein gamma, N-terminally processed	YWHAG	41.3	0.7668
P09211	Glutathione S-transferase P	GSTP1	57.6	0.77089
P49915	GMP synthase [glutamine-hydrolyzing]	GMPS	4.2	0.77115
P49419	Alpha-aminoadipic semialdehyde dehydrogenase	ALDH7A1	5.4	0.77167
Q8IVL6	Prolyl 3-hydroxylase 3	LEPREL2	2.7	0.77522
P61221	ATP-binding cassette sub-family E member 1	ABCE1	5.7	0.77589
P28074	Proteasome subunit beta type-5	PSMB5	13.3	0.77916
P49411	Elongation factor Tu, mitochondrial	TUFM	17.5	0.77917
P28838	Cytosol aminopeptidase	LAP3	15.8	0.77948
O00244	Copper transport protein ATOX1	ATOX1	45.6	0.77976
P23229	Integrin alpha-6;Integrin alpha-6 heavy chain;Integrin alpha-6 light chain	ITGA6	3.1	0.77983
Q9UBE0	SUMO-activating enzyme subunit 1	SAE1	7.5	0.78022
P60174	Triosephosphate isomerase	TPI1	46.2	0.78086
P55145	Mesencephalic astrocyte-derived neurotrophic factor	MANF	23.1	0.78422
P25786	Proteasome subunit alpha type-1	PSMA1	22.1	0.78471
P49773	Histidine triad nucleotide-binding protein 1	HINT1	27.8	0.78651
P23381	Tryptophan--tRNA ligase, cytoplasmic;T1-TrpRS;T2-TrpRS	WARS	10.2	0.78658
Q99829	Copine-1	CPNE1	7.3	0.78736
O76003	Glutaredoxin-3	GLRX3	28.4	0.79
O00560	Syntenin-1	SDCBP	11.4	0.79102
P61960	Ubiquitin-fold modifier 1	UFM1	58.8	0.79204
P29401	Transketolase	TKT	26	0.79253
P78417	Glutathione S-transferase omega-1	GSTO1	41.5	0.79298
Q13435	Splicing factor 3B subunit 2	SF3B2	5	0.79322

P07339	Cathepsin D;Cathepsin D light chain;Cathepsin D heavy chain	CTSD	17.7	0.7935
Q8NBS9	Thioredoxin domain-containing protein 5	TXNDC5	13.2	0.79597
Q99873;Q9NR22	Protein arginine N-methyltransferase 1	PRMT1	19.4	0.79622
P17301	Integrin alpha-2	ITGA2	9.2	0.79664
P55263	Adenosine kinase	ADK	18.5	0.79739
P49588	Alanine--tRNA ligase, cytoplasmic	AARS	16.2	0.79957
P13693;Q56UQ5	Translationally-controlled tumor protein	TPT1	32	0.80023
Q9NRX4	14 kDa phosphohistidine phosphatase	PHPT1	38.4	0.80045
P31939	Bifunctional purine biosynthesis protein PURH;Phosphoribosylaminoimidazolecarboxamide formyltransferase;IMP cyclohydrolase	ATIC	23.5	0.80056
Q92905	COP9 signalosome complex subunit 5	COPS5	11.7	0.80224
P84098	60S ribosomal protein L19	RPL19	8.7	0.80271
P50395	Rab GDP dissociation inhibitor beta	GDI2	40.9	0.80347
Q9Y3F4	Serine-threonine kinase receptor-associated protein	STRAP	35.1	0.80351
P63244	Guanine nucleotide-binding protein subunit beta-2-like 1	GNB2L1	45.1	0.80493
P13639	Elongation factor 2	EEF2	38.9	0.80534
P31150	Rab GDP dissociation inhibitor alpha	GDI1	32.2	0.80616
O75390	Citrate synthase, mitochondrial	CS	9	0.80795
P61009	Signal peptidase complex subunit 3	SPCS3	12.8	0.80813
P40926	Malate dehydrogenase, mitochondrial	MDH2	45	0.809
P16152	Carbonyl reductase [NADPH] 1	CBR1	26.4	0.81063
P62714;P67775	Serine/threonine-protein phosphatase 2A catalytic subunit beta isoform;Serine/threonine-protein phosphatase 2A catalytic subunit alpha isoform	PPP2CB;PPP2CA	12.9	0.81133
P60900	Proteasome subunit alpha type-6	PSMA6	25.6	0.81243
O75874	Isocitrate dehydrogenase [NADP] cytoplasmic	IDH1	13.8	0.8135
P27797	Calreticulin	CALR	47	0.8159
P22392;O60361	Nucleoside diphosphate kinase B;Putative nucleoside diphosphate kinase	NME2;NME2P1	40.1	0.81592
P37837	Transaldolase	TALDO1	37.7	0.81595
Q14019	Coactosin-like protein	COTL1	33.8	0.81869
P08865	40S ribosomal protein SA	RPSA	23.7	0.81906

P00558;P07205	Phosphoglycerate kinase 1	PGK1	56.1	0.81947
P27658	Collagen alpha-1(VIII) chain;Vastatin	COL8A1	3.2	0.82027
P49023	Paxillin	PXN	13.2	0.82237
Q16881;Q9NNW7	Thioredoxin reductase 1, cytoplasmic	TXNRD1	35.1	0.82238
P11586	C-1-tetrahydrofolate synthase, cytoplasmic;Methylenetetrahydrofolate dehydrogenase;Methenyltetrahydrofolate cyclohydrolase;Formyltetrahydrofolate synthetase	MTHFD1	14.3	0.82261
Q13185;P83916	Chromobox protein homolog 3	CBX3	30.1	0.82289
P11766	Alcohol dehydrogenase class-3	ADH5	41.4	0.82353
P26368	Splicing factor U2AF 65 kDa subunit	U2AF2	9.9	0.82367
P63220	40S ribosomal protein S21	RPS21	30.1	0.82478
P04406	Glyceraldehyde-3-phosphate dehydrogenase	GAPDH	45.1	0.82517
P49591	Serine--tRNA ligase, cytoplasmic	SARS	16.9	0.82588
P16401	Histone H1.5	HIST1H1B	11.9	0.8261
O14561	Acyl carrier protein, mitochondrial	NDUFAB1	12.2	0.82624
Q13509;CON__ENSEMBL:ENSBTAP00000025008;Q9H4B7;A6NKZ8	Tubulin beta-3 chain	TUBB3	29.6	0.82628
Q01105	Protein SET	SET	18.3	0.82651
P39748	Flap endonuclease 1	FEN1	12.1	0.82686
P51858	Hepatoma-derived growth factor	HDGF	17.1	0.82749
P63151;Q66LE6;Q00005	Serine/threonine-protein phosphatase 2A 55 kDa regulatory subunit B alpha isoform	PPP2R2A	9.8	0.82756
P35232	Prohibitin	PHB	21.3	0.82788
P55884	Eukaryotic translation initiation factor 3 subunit B	EIF3B	8.4	0.82919
P30041	Peroxiredoxin-6	PRDX6	36.2	0.83006
P00505	Aspartate aminotransferase, mitochondrial	GOT2	11.9	0.83089
Q13501	Sequestosome-1	SQSTM1	10.2	0.83125
P07954	Fumarate hydratase, mitochondrial	FH	19	0.83256

P21796	Voltage-dependent anion-selective channel protein 1	VDAC1	45.9	0.83345
P58546	Myotrophin	MTPN	46.6	0.83354
P30043	Flavin reductase (NADPH)	BLVRB	23.8	0.83402
P51149	Ras-related protein Rab-7a	RAB7A	34.8	0.83576
P14550	Alcohol dehydrogenase [NADP(+)]	AKR1A1	27.1	0.83626
P13010	X-ray repair cross-complementing protein 5	XRCC5	27.3	0.83759
P30048	Thioredoxin-dependent peroxide reductase, mitochondrial	PRDX3	20.7	0.8381
P13667	Protein disulfide-isomerase A4	PDIA4	22.3	0.83829
P36776	Lon protease homolog, mitochondrial	LONP1	2.6	0.83909
P54886	Delta-1-pyrroline-5-carboxylate synthase;Glutamate 5-kinase;Gamma-glutamyl phosphate reductase	ALDH18A1	6.9	0.84017
P12236;P12235	ADP/ATP translocase 3	SLC25A6	34.6	0.84178
P23284	Peptidyl-prolyl cis-trans isomerase B	PPIB	38.9	0.84316
P23526	Adenosylhomocysteinase	AHCY	14.1	0.84326
P13674	Prolyl 4-hydroxylase subunit alpha-1	P4HA1	15.5	0.84347
P07686	Beta-hexosaminidase subunit beta;Beta-hexosaminidase subunit beta chain B;Beta-hexosaminidase subunit beta chain A	HEXB	15.1	0.84373
P63241;Q9GZV4;Q6IS14	Eukaryotic translation initiation factor 5A-1;Eukaryotic translation initiation factor 5A-2;Eukaryotic translation initiation factor 5A-1-like	EIF5A;EIF5A2;EIF5AL1	44.8	0.84416
P52597	Heterogeneous nuclear ribonucleoprotein F;Heterogeneous nuclear ribonucleoprotein F, N-terminally processed	HNRNPF	16.4	0.84489
P50502;Q8NFI4;Q8IZP2	Hsc70-interacting protein;Putative protein FAM10A5;Putative protein FAM10A4	ST13;ST13P5;ST13P4	8.1	0.84543
Q14956	Transmembrane glycoprotein NMB	GPNMB	4.7	0.84546
P25789	Proteasome subunit alpha type-4	PSMA4	32.2	0.84617
O60701	UDP-glucose 6-dehydrogenase	UGDH	27.1	0.84638
O00299	Chloride intracellular channel protein 1	CLIC1	51	0.84806
P27695	DNA-(apurinic or apyrimidinic site) lyase;DNA-(apurinic or apyrimidinic site) lyase,	APEX1	11.3	0.84876

	mitochondrial			
P62258	14-3-3 protein epsilon	YWHAE	42.7	0.84884
P07858	Cathepsin B;Cathepsin B light chain;Cathepsin B heavy chain	CTSB	21.2	0.85128
P05387	60S acidic ribosomal protein P2	RPLP2	60.9	0.85422
Q13596	Sorting nexin-1	SNX1	4.6	0.85474
P80723	Brain acid soluble protein 1	BASP1	54.6	0.85525
Q9HB71	Calcyclin-binding protein	CACYBP	32.9	0.85534
P18669;Q8N0Y7;P15259	Phosphoglycerate mutase 1;Probable phosphoglycerate mutase 4;Phosphoglycerate mutase 2	PGAM1;PGAM4;PGAM2	35	0.85577
O75533	Splicing factor 3B subunit 1	SF3B1	3	0.85791
P25787	Proteasome subunit alpha type-2	PSMA2	27.8	0.85897
P06733;P09104;P13929	Alpha-enolase	ENO1	51.8	0.85942
Q93009	Ubiquitin carboxyl-terminal hydrolase 7	USP7	4.3	0.85959
P22307	Non-specific lipid-transfer protein	SCP2	19.9	0.85971
P48147	Prolyl endopeptidase	PREP	8.5	0.86
O75347	Tubulin-specific chaperone A	TBCA	26.9	0.86129
P17096	High mobility group protein HMG-I/HMG-Y	HMGA1	23.4	0.86138
P13804	Electron transfer flavoprotein subunit alpha, mitochondrial	ETFA	35.7	0.86152
P30101	Protein disulfide-isomerase A3	PDIA3	30.7	0.86515
P07195	L-lactate dehydrogenase B chain	LDHB	42.5	0.8663
P30084	Enoyl-CoA hydratase, mitochondrial	ECHS1	20.7	0.8677
P26447	Protein S100-A4	S100A4	27.7	0.86897
Q5JPE7;P69849;Q15155	Nodal modulator 2;Nodal modulator 3;Nodal modulator 1	NOMO2;NOMO3;NOMO1	4.3	0.87104
P31946	14-3-3 protein beta/alpha;14-3-3 protein beta/alpha, N-terminally processed	YWHAB	39.4	0.87147
Q13765;Q9BZK3	Nascent polypeptide-associated complex subunit alpha	NACA	19.5	0.87224
P84090	Enhancer of rudimentary homolog	ERH	33.7	0.87227
P41567;O60739	Eukaryotic translation initiation factor 1;Eukaryotic translation initiation factor 1b	EIF1;EIF1B	23.9	0.8727
P52565	Rho GDP-dissociation inhibitor 1	ARHGDIA	31.4	0.87272
P11177	Pyruvate dehydrogenase E1 component subunit	PDHB	5.6	0.87279

	beta, mitochondrial			
P11021	78 kDa glucose-regulated protein	HSPA5	28.9	0.873
P09651;Q32P51	Heterogeneous nuclear ribonucleoprotein A1;Heterogeneous nuclear ribonucleoprotein A1-like 2	HNRNPA1;HNRNPA1L2	19.4	0.87315
P52209	6-phosphogluconate dehydrogenase, decarboxylating	PGD	20.5	0.87341
Q01813	6-phosphofructokinase type C	PFKP	15.7	0.87548
P29144	Tripeptidyl-peptidase 2	TPP2	7.3	0.87554
P08758	Annexin A5	ANXA5	30.3	0.87601
Q9Y277	Voltage-dependent anion-selective channel protein 3	VDAC3	38.9	0.87706
Q9ULC4	Malignant T-cell-amplified sequence 1	MCTS1	40.9	0.87732
P55072	Transitional endoplasmic reticulum ATPase	VCP	30.4	0.87851
Q13263	Transcription intermediary factor 1-beta	TRIM28	7.9	0.87945
O15372	Eukaryotic translation initiation factor 3 subunit H	EIF3H	14.2	0.87957
Q13200	26S proteasome non-ATPase regulatory subunit 2	PSMD2	18.1	0.87957
Q6YN16	Hydroxysteroid dehydrogenase-like protein 2	HSDL2	8.9	0.88068
P0C7P4;P47985	Putative cytochrome b-c1 complex subunit Rieske-like protein 1;Cytochrome b-c1 complex subunit Rieske, mitochondrial;Cytochrome b-c1 complex subunit 11	UQCRFS1P1;UQCRFS1	7.4	0.88108
P09429;B2RPK0;P23497	High mobility group protein B1	HMGB1	38.6	0.88166
P17174	Aspartate aminotransferase, cytoplasmic	GOT1	23.7	0.88229
Q04837	Single-stranded DNA-binding protein, mitochondrial	SSBP1	27.7	0.88257
Q04446	1,4-alpha-glucan-branching enzyme	GBE1	12.4	0.88363
Q16527	Cysteine and glycine-rich protein 2	CSRP2	14	0.88417
P00367;P49448	Glutamate dehydrogenase 1, mitochondrial;Glutamate dehydrogenase 2, mitochondrial	GLUD1;GLUD2	20.3	0.88666
O00505	Importin subunit alpha-3	KPNA3	13.1	0.88689
Q9UNZ2	NSFL1 cofactor p47	NSFL1C	13.5	0.88752
P14314	Glucosidase 2 subunit beta	PRKCSH	16.7	0.88902
P28066	Proteasome subunit alpha type-5	PSMA5	25.7	0.88932
Q92734	Protein TFG	TFG	6.8	0.89056

Q16891	Mitochondrial inner membrane protein	IMMT	13.6	0.89077
Q13838;O00148	Spliceosome RNA helicase DDX39B	DDX39B	29.7	0.89116
Q92945	Far upstream element-binding protein 2	KHSRP	8.3	0.89116
O75947	ATP synthase subunit d, mitochondrial	ATP5H	47.2	0.89123
Q9Y266	Nuclear migration protein nudC	NUDC	11.2	0.89248
O60814;P57053;P58876;P62807;Q5QNW6;Q93079;Q99877;Q99879;Q99880;Q96A08	Histone H2B type 1-K;Histone H2B type F-S;Histone H2B type 1-D;Histone H2B type 1-C/E/F/G/I;Histone H2B type 2-F;Histone H2B type 1-H;Histone H2B type 1-N;Histone H2B type 1-M;Histone H2B type 1-L	HIST1H2BK;H2BFS;HIST1H2BD;HIST1H2BC;HIST2H2BF;HIST1H2BH;HIST1H2BN;HIST1H2BM;HIST1H2BL	16.7	0.89336
Q9Y4L1	Hypoxia up-regulated protein 1	HYOU1	15.8	0.89408
P46108	Adapter molecule crk	CRK	14.8	0.89419
Q15293	Reticulocalbin-1	RCN1	19.9	0.89529
Q99497	Protein DJ-1	PARK7	52.4	0.89538
P45880	Voltage-dependent anion-selective channel protein 2	VDAC2	30.3	0.89569
Q9HC98;Q8TDX7	Serine/threonine-protein kinase Nek6;Serine/threonine-protein kinase Nek7	NEK6;NEK7	13.4	0.89601
P62195	26S protease regulatory subunit 8	PSMC5	17	0.89654
P61289	Proteasome activator complex subunit 3	PSME3	9.4	0.89736
O43684	Mitotic checkpoint protein BUB3	BUB3	8.2	0.89758
P31943	Heterogeneous nuclear ribonucleoprotein H;Heterogeneous nuclear ribonucleoprotein H, N-terminally processed	HNRNPH1	17.1	0.89978
P46779	60S ribosomal protein L28	RPL28	19	0.90033
P04350	Tubulin beta-4A chain	TUBB4A	23.2	0.90078
Q9NTK5	Obg-like ATPase 1	OLA1	9.8	0.90168
P49720	Proteasome subunit beta type-3	PSMB3	22.4	0.90224
Q9Y6M1	Insulin-like growth factor 2 mRNA-binding protein 2	IGF2BP2	6	0.90285
Q99471	Prefoldin subunit 5	PFDN5	33.1	0.90332
Q6NUK1	Calcium-binding mitochondrial carrier protein	SLC25A24	4.2	0.90395

	SCaMC-1			
P46060	Ran GTPase-activating protein 1	RANGAP1	7.7	0.90427
P25398	40S ribosomal protein S12	RPS12	62.9	0.90458
Q9C0H2	Protein tweety homolog 3	TTYH3	2.7	0.90508
P22234	Multifunctional protein ADE2;Phosphoribosylaminoimidazole-succinocarboxamide synthase;Phosphoribosylaminoimidazole carboxylase	PAICS	21.2	0.90512
O75718	Cartilage-associated protein	CRTAP	13.7	0.90526
P04181	Ornithine aminotransferase, mitochondrial;Ornithine aminotransferase, hepatic form;Ornithine aminotransferase, renal form	OAT	13.9	0.90757
P48047	ATP synthase subunit O, mitochondrial	ATP5O	9.9	0.90892
O14737	Programmed cell death protein 5	PDCD5	18.4	0.90933
P35580	Myosin-10	MYH10	18.1	0.90999
O14818;Q8TAA3	Proteasome subunit alpha type-7	PSMA7	21.8	0.9118
Q04917	14-3-3 protein eta	YWHAH	37	0.91438
P49189	4-trimethylaminobutyraldehyde dehydrogenase	ALDH9A1	19.6	0.9147
P06748	Nucleophosmin	NPM1	19.7	0.91573
P48643	T-complex protein 1 subunit epsilon	CCT5	26.2	0.91578
P06899;P23527;P33778;Q16778;Q8N257;Q6DN03;Q6DRA6	Histone H2B type 1-J;Histone H2B type 1-O;Histone H2B type 1-B;Histone H2B type 2-E;Histone H2B type 3-B;Putative histone H2B type 2-C;Putative histone H2B type 2-D	HIST1H2BJ;HIST1H2BO;HIST1H2BB;HIST2H2BE;HIST3H2BB;HIST2H2BC;HIST2H2BD	16.7	0.9169
P68036	Ubiquitin-conjugating enzyme E2 L3	UBE2L3	43.5	0.91778
P78371	T-complex protein 1 subunit beta	CCT2	43.9	0.91832
P21589	5-nucleotidase	NT5E	23.7	0.91962
P61769	Beta-2-microglobulin;Beta-2-microglobulin form pI 5.3	B2M	35.3	0.92027
P09936	Ubiquitin carboxyl-terminal hydrolase isozyme L1	UCHL1	46.6	0.9206
P60842	Eukaryotic initiation factor 4A-I	EIF4A1	24.4	0.92122
P48681	Nestin	NES	7.2	0.92172
Q06830	Peroxiredoxin-1	PRDX1	55.8	0.92205

P22102	Trifunctional purine biosynthetic protein adenosine-3;Phosphoribosylamine--glycine ligase;Phosphoribosylformylglycinamidine cyclo-ligase;Phosphoribosylglycinamide formyltransferase	GART	6.5	0.92212
Q9UQ80	Proliferation-associated protein 2G4	PA2G4	28.9	0.92307
P24752	Acetyl-CoA acetyltransferase, mitochondrial	ACAT1	23.2	0.92311
P17980	26S protease regulatory subunit 6A	PSMC3	21.2	0.92422
P29317	Ephrin type-A receptor 2	EPHA2	3.5	0.92436
P23528	Cofilin-1	CFL1	71.1	0.92449
Q86V81	THO complex subunit 4	ALYREF	13.2	0.92502
O00231	26S proteasome non-ATPase regulatory subunit 11	PSMD11	20.9	0.9252
P62136;P36873	Serine/threonine-protein phosphatase PP1-alpha catalytic subunit;Serine/threonine-protein phosphatase PP1-gamma catalytic subunit	PPP1CA;PPP1CC	28.8	0.92541
Q99798	Aconitate hydratase, mitochondrial	ACO2	8.3	0.92758
P25788	Proteasome subunit alpha type-3	PSMA3	12.9	0.92768
P05388;Q8NHW5	60S acidic ribosomal protein P0;60S acidic ribosomal protein P0-like	RPLP0;RPLP0P6	40.7	0.92779
P62979;P0CG48;P0CG47;P62987	Ubiquitin-40S ribosomal protein S27a;Ubiquitin;40S ribosomal protein S27a;Polyubiquitin-C;Ubiquitin;Polyubiquitin-B;Ubiquitin;Ubiquitin-60S ribosomal protein L40;Ubiquitin;60S ribosomal protein L40	RPS27A;UBC;UBB;UBA52	18.6	0.92796
P30086	Phosphatidylethanolamine-binding protein 1;Hippocampal cholinergic neurostimulating peptide	PEBP1	55.6	0.92862
P68371	Tubulin beta-4B chain	TUBB4B	24.7	0.92924
P41250	Glycine--tRNA ligase	GARS	19.5	0.92972
P42166;P42167	Lamina-associated polypeptide 2, isoform alpha;Thymopoietin;Thymopentin;Lamina-associated polypeptide 2, isoforms beta/gamma;Thymopoietin;Thymopentin	TMPO	5.8	0.93019
O43324	Eukaryotic translation elongation factor 1 epsilon-1	EEF1E1	20.7	0.93026
Q04760	Lactoylglutathione lyase	GLO1	39.1	0.93157
P11142	Heat shock cognate 71 kDa protein	HSPA8	41.8	0.93167
P49368	T-complex protein 1 subunit gamma	CCT3	19.8	0.93305
P35222;P14	Catenin beta-1;Junction plakoglobin	CTNNB1;J	4.5	0.93306

923		UP		
P35613	Basigin	BSG	7.8	0.93331
P18085;P84085	ADP-ribosylation factor 4;ADP-ribosylation factor 5	ARF4;ARF5	31.7	0.93361
P50990	T-complex protein 1 subunit theta	CCT8	24.1	0.93453
P27348	14-3-3 protein theta	YWHAQ	44.9	0.93482
P60228	Eukaryotic translation initiation factor 3 subunit E	EIF3E	18.4	0.93512
P36405	ADP-ribosylation factor-like protein 3	ARL3	16.5	0.93534
Q15181	Inorganic pyrophosphatase	PPA1	47.8	0.93634
P55786;A6NEC2	Puromycin-sensitive aminopeptidase	NPEPPS	11.8	0.93678
P38606	V-type proton ATPase catalytic subunit A	ATP6V1A	13	0.9368
Q06210;O94808	Glucosamine--fructose-6-phosphate aminotransferase [isomerizing] 1	GFPT1	15	0.93685
P54577	Tyrosine--tRNA ligase, cytoplasmic	YARS	10.6	0.93694
P06730;A6NMX2	Eukaryotic translation initiation factor 4E;Eukaryotic translation initiation factor 4E type 1B	EIF4E;EIF4E1B	8.3	0.93768
P62917	60S ribosomal protein L8	RPL8	21.8	0.93778
P22061	Protein-L-isoaspartate(D-aspartate) O-methyltransferase	PCMT1	15	0.93857
P61247	40S ribosomal protein S3a	RPS3A	32.2	0.93879
Q92747	Actin-related protein 2/3 complex subunit 1A	ARPC1A	7.8	0.93889
P22626	Heterogeneous nuclear ribonucleoproteins A2/B1	HNRNPA2B1	7.9	0.93951
P55036;A2A3N6	26S proteasome non-ATPase regulatory subunit 4	PSMD4	21	0.94044
P38919	Eukaryotic initiation factor 4A-III	EIF4A3	7.3	0.94109
Q9NP79	Vacuolar protein sorting-associated protein VTA1 homolog	VTA1	11.1	0.94159
P61254;Q9UNX3	60S ribosomal protein L26;60S ribosomal protein L26-like 1	RPL26;RPL26L1	15.2	0.94178
P22314	Ubiquitin-like modifier-activating enzyme 1	UBA1	18	0.94208
P07237	Protein disulfide-isomerase	P4HB	47	0.94227
P47755	F-actin-capping protein subunit alpha-2	CAPZA2	32.2	0.9424
P25705	ATP synthase subunit alpha, mitochondrial	ATP5A1	21.5	0.9452
P62841	40S ribosomal protein S15	RPS15	42.8	0.94562
P14866	Heterogeneous nuclear ribonucleoprotein L	HNRNPL	30.4	0.94593

Q96QR8	Transcriptional activator protein Pur-beta	PURB	14.1	0.94653
P52907	F-actin-capping protein subunit alpha-1	CAPZA1	33.6	0.94679
Q9Y230	RuvB-like 2	RUVBL2	6	0.94687
Q15758	Neutral amino acid transporter B(0)	SLC1A5	8.7	0.94692
P12956	X-ray repair cross-complementing protein 6	XRCC6	27.6	0.94733
P63000	Ras-related C3 botulinum toxin substrate 1	RAC1	20.8	0.94769
P20700	Lamin-B1	LMNB1	8.4	0.94787
P06576	ATP synthase subunit beta, mitochondrial	ATP5B	25.3	0.94795
Q9UJ70	N-acetyl-D-glucosamine kinase	NAGK	18.6	0.9483
Q13283	Ras GTPase-activating protein-binding protein 1	G3BP1	15.9	0.9488
P11216	Glycogen phosphorylase, brain form	PYGB	10	0.9491
Q14697	Neutral alpha-glucosidase AB	GANAB	6.9	0.94989
P52272	Heterogeneous nuclear ribonucleoprotein M	HNRNPM	10.8	0.95002
O43776	Asparagine--tRNA ligase, cytoplasmic	NARS	5.5	0.9501
P35998	26S protease regulatory subunit 7	PSMC2	15.5	0.95078
Q9Y5S9	RNA-binding protein 8A	RBM8A	21.3	0.95094
P04075;P09972;P05062	Fructose-bisphosphate aldolase A	ALDOA	53.8	0.95212
Q15365	Poly(rC)-binding protein 1	PCBP1	27.2	0.95216
Q96QK1	Vacuolar protein sorting-associated protein 35	VPS35	15.1	0.95216
P62314	Small nuclear ribonucleoprotein Sm D1	SNRPD1	26.1	0.95245
Q13310	Polyadenylate-binding protein 4	PABPC4	11	0.95351
P30153	Serine/threonine-protein phosphatase 2A 65 kDa regulatory subunit A alpha isoform	PPP2R1A	27.8	0.95379
Q9NZ08	Endoplasmic reticulum aminopeptidase 1	ERAP1	3.5	0.95536
P53621	Coatomer subunit alpha;Xenin;Proxenin	COPA	11.6	0.95598
P62805	Histone H4	HIST1H4A	23.3	0.95598
Q99733	Nucleosome assembly protein 1-like 4	NAP1L4	19.5	0.95626
P19338	Nucleolin	NCL	24.9	0.95688
Q9UBQ0	Vacuolar protein sorting-associated protein 29	VPS29	14.3	0.95724
Q32P28	Prolyl 3-hydroxylase 1	LEPRE1	9.9	0.95793
P14868	Aspartate--tRNA ligase, cytoplasmic	DARS	10	0.9591
P26641	Elongation factor 1-gamma	EEF1G	18.3	0.96142
Q13347	Eukaryotic translation initiation factor 3 subunit I	EIF3I	28	0.96165
P62937;A2BFH1;Q9Y536	Peptidyl-prolyl cis-trans isomerase A	PPIA	55.2	0.96256

P29692	Elongation factor 1-delta	EEF1D	16.7	0.96309
P36578	60S ribosomal protein L4	RPL4	13.3	0.9632
Q9BVK6;Q7Z7H5	Transmembrane emp24 domain-containing protein 9	TMED9	14.9	0.96325
P63208	S-phase kinase-associated protein 1	SKP1	35.6	0.96362
Q14157	Ubiquitin-associated protein 2-like	UBAP2L	10.6	0.96383
P04080	Cystatin-B	CSTB	55.1	0.96419
P42704	Leucine-rich PPR motif-containing protein, mitochondrial	LRPPRC	13.3	0.96481
Q96TA1	Niban-like protein 1	FAM129B	10.7	0.96515
Q14683	Structural maintenance of chromosomes protein 1A	SMC1A	2.4	0.96544
P15121	Aldose reductase	AKR1B1	36.1	0.96689
P09960	Leukotriene A-4 hydrolase	LTA4H	13.4	0.96698
P17987	T-complex protein 1 subunit alpha	TCP1	33.6	0.96708
P09497	Clathrin light chain B	CLTB	13.1	0.96755
P26196	Probable ATP-dependent RNA helicase DDX6	DDX6	15.5	0.96825
P43686	26S protease regulatory subunit 6B	PSMC4	11.2	0.96883
P50914	60S ribosomal protein L14	RPL14	10.2	0.96904
P07099	Epoxide hydrolase 1	EPHX1	11.6	0.96952
Q5JRX3	Presequence protease, mitochondrial	PITRM1	6	0.96991
P21399	Cytoplasmic aconitate hydratase	ACO1	9.7	0.97011
P29966	Myristoylated alanine-rich C-kinase substrate	MARCKS	19.6	0.97014
Q96EY7	Pentatricopeptide repeat-containing protein 3, mitochondrial	PTCD3	4.2	0.97054
P24539	ATP synthase subunit b, mitochondrial	ATP5F1	10.5	0.97057
Q9Y224	UPF0568 protein C14orf166	C14orf166	27.9	0.97069
P55209	Nucleosome assembly protein 1-like 1	NAP1L1	17.4	0.97089
Q58FF8	Putative heat shock protein HSP 90-beta 2	HSP90AB2P	10.8	0.97152
P24534	Elongation factor 1-beta	EEF1B2	14.7	0.9721
P43034	Platelet-activating factor acetylhydrolase IB subunit alpha	PAFAH1B1	10.7	0.97212
Q02543	60S ribosomal protein L18a	RPL18A	42	0.97291
Q9Y333	U6 snRNA-associated Sm-like protein LSm2	LSM2	20	0.97343
P62316	Small nuclear ribonucleoprotein Sm D2	SNRPD2	26.3	0.97441
P62753	40S ribosomal protein S6	RPS6	15.3	0.97441
P14618;P30	Pyruvate kinase isozymes M1/M2	PKM2	48.4	0.97473

613				
P39023;Q92901	60S ribosomal protein L3	RPL3	34.2	0.97531
Q15233	Non-POU domain-containing octamer-binding protein	NONO	6.4	0.97569
O95816	BAG family molecular chaperone regulator 2	BAG2	26.1	0.97656
Q9H4A4	Aminopeptidase B	RNPEP	13.4	0.97672
P30044	Peroxiredoxin-5, mitochondrial	PRDX5	36.9	0.9769
P22695	Cytochrome b-c1 complex subunit 2, mitochondrial	UQCRC2	10.4	0.97738
Q14103	Heterogeneous nuclear ribonucleoprotein D0	HNRNPD	26.8	0.97768
P04844	Dolichyl-diphosphooligosaccharide--protein glycosyltransferase subunit 2	RPN2	13.8	0.97818
P40925	Malate dehydrogenase, cytoplasmic	MDH1	20.7	0.97857
P49207	60S ribosomal protein L34	RPL34	21.4	0.97883
P50991	T-complex protein 1 subunit delta	CCT4	21.3	0.97935
Q13740	CD166 antigen	ALCAM	11	0.97974
P62424	60S ribosomal protein L7a	RPL7A	34.2	0.97987
P46777	60S ribosomal protein L5	RPL5	24.6	0.98045
Q9Y305	Acyl-coenzyme A thioesterase 9, mitochondrial	ACOT9	11.2	0.98324
P07437	Tubulin beta chain	TUBB	24.8	0.98379
P47756	F-actin-capping protein subunit beta	CAPZB	18.4	0.98451
P62241	40S ribosomal protein S8	RPS8	15.4	0.98537
Q9UHD1	Cysteine and histidine-rich domain-containing protein 1	CHORDC1	9	0.98588
P14625;Q58FF3	Endoplasmin	HSP90B1	33.4	0.98611
O43143	Putative pre-mRNA-splicing factor ATP-dependent RNA helicase DHX15	DHX15	4.5	0.98674
Q96M27	Protein PRRC1	PRRC1	7.4	0.98712
P05455	Lupus La protein	SSB	20.6	0.98782
P09382	Galectin-1	LGALS1	48.1	0.98784
P55060	Exportin-2	CSE1L	11	0.98866
Q3ZCM7	Tubulin beta-8 chain	TUBB8	13.7	0.989
P09486	SPARC	SPARC	12.2	0.98906
Q9HD45	Transmembrane 9 superfamily member 3	TM9SF3	3.4	0.98943
Q9NX40	OCIA domain-containing protein 1	OCIAD1	12.7	0.98952
P62750	60S ribosomal protein L23a	RPL23A	42.9	0.98956

P40227;Q92526	T-complex protein 1 subunit zeta	CCT6A	43.5	0.99023
Q02809	Procollagen-lysine,2-oxoglutarate 5-dioxygenase 1	PLOD1	12.2	0.99056
Q6DKJ4	Nucleoredoxin	NXN	4.8	0.99082
O43768;P56211	Alpha-endosulfine;cAMP-regulated phosphoprotein 19	ENSA;ARPP19	28.1	0.99103
P62266	40S ribosomal protein S23	RPS23	15.4	0.9912
P12081	Histidine--tRNA ligase, cytoplasmic	HARS	15.1	0.99163
Q9NR30;Q9BQ39	Nucleolar RNA helicase 2	DDX21	6.4	0.99213
P08238;Q58FF7	Heat shock protein HSP 90-beta	HSP90AB1	24.3	0.99239
O76094	Signal recognition particle 72 kDa protein	SRP72	10.9	0.9943
P07737;CON__P02584	Profilin-1	PFN1	75	0.99431
P04083	Annexin A1	ANXA1	46	0.99434
Q99729	Heterogeneous nuclear ribonucleoprotein A/B	HNRNPAB	6.9	0.99453
P09110	3-ketoacyl-CoA thiolase, peroxisomal	ACAA1	5	0.99471
P61923	Coatomer subunit zeta-1	COPZ1	13.6	0.99477
P50479	PDZ and LIM domain protein 4	PDLIM4	9.4	0.99478
P53992	Protein transport protein Sec24C	SEC24C	2.7	0.99572
Q14257	Reticulocalbin-2	RCN2	11.7	0.99595
Q02878	60S ribosomal protein L6	RPL6	21.9	0.9964
Q02818	Nucleobindin-1	NUCB1	4.6	0.99695
O60888	Protein CutA	CUTA	23.5	0.99711
P26599;O95758	Polypyrimidine tract-binding protein 1	PTBP1	23.9	0.99764
P62191	26S protease regulatory subunit 4	PSMC1	6.8	0.99788
Q96CS3	FAS-associated factor 2	FAF2	7.9	0.99798
Q9P0K7	Ankycorbin	RAI14	5	0.99798
P35606	Coatomer subunit beta	COPB2	8.5	0.99891
O00203;Q13367	AP-3 complex subunit beta-1;AP-3 complex subunit beta-2	AP3B1;AP3B2	3.4	0.999
P49257	Protein ERGIC-53	LMAN1	7.1	0.99907
P60033	CD81 antigen	CD81	25	0.99917
P08195	4F2 cell-surface antigen heavy chain	SLC3A2	13.5	0.99926
Q96KP4	Cytosolic non-specific dipeptidase	CNDP2	16.6	1.0004

Q9Y394	Dehydrogenase/reductase SDR family member 7	DHRS7	14.2	1.0008
P00568	Adenylate kinase isoenzyme 1	AK1	17.5	1.0016
O00303	Eukaryotic translation initiation factor 3 subunit F	EIF3F	12.9	1.0022
P04843	Dolichyl-diphosphooligosaccharide--protein glycosyltransferase subunit 1	RPN1	21.7	1.0026
P61313	60S ribosomal protein L15	RPL15	24	1.0028
P02786	Transferrin receptor protein 1;Transferrin receptor protein 1, serum form	TFRC	7.9	1.0032
O15305	Phosphomannomutase 2	PMM2	9.3	1.0038
P35237	Serpin B6	SERPINB6	25.3	1.0041
Q00839	Heterogeneous nuclear ribonucleoprotein U	HNRNPU	16.8	1.0047
P04908;P0C0S8;P20671;Q6FI13;Q7L7L0;Q93077;Q16777;Q9BTM1;Q96KK5;Q99878	Histone H2A type 1-B/E;Histone H2A type 1;Histone H2A type 1-D;Histone H2A type 2-A;Histone H2A type 3;Histone H2A type 1-C;Histone H2A type 2-C;Histone H2A.J;Histone H2A type 1-H;Histone H2A type 1-J	HIST1H2AB;HIST1H2AG;HIST1H2AD;HIST2H2AA3;HIST3H2A;HIST1H2AC;HIST2H2AC;H2AFJ;HIST1H2AH;HIST1H2AJ	26.2	1.005
Q15366;P57721	Poly(rC)-binding protein 2	PCBP2	28.2	1.005
Q9Y5Y2	Cytosolic Fe-S cluster assembly factor NUBP2	NUBP2	19.6	1.0064
O00622	Protein CYR61	CYR61	22	1.0073
O00487	26S proteasome non-ATPase regulatory subunit 14	PSMD14	28.7	1.0086
P05386	60S acidic ribosomal protein P1	RPLP1	74.6	1.0092
O14579	Coatomer subunit epsilon	COPE	21.1	1.011
O95336	6-phosphogluconolactonase	PGLS	17.4	1.0112
P05023;P50993;P13637;P54707;Q13733	Sodium/potassium-transporting ATPase subunit alpha-1	ATP1A1	10.4	1.0113
Q14444	Caprin-1	CAPRIN1	15.2	1.012
P53618	Coatomer subunit beta	COPB1	5.8	1.0121
Q92841	Probable ATP-dependent RNA helicase DDX17	DDX17	16.2	1.0123

O43390	Heterogeneous nuclear ribonucleoprotein R	HNRNPR	11.2	1.0129
O95394	Phosphoacetylglucosamine mutase	PGM3	5	1.014
O14979	Heterogeneous nuclear ribonucleoprotein D-like	HNRPDL	13.3	1.0141
P50995	Annexin A11	ANXA11	8.9	1.0144
Q99832	T-complex protein 1 subunit eta	CCT7	33	1.0144
Q9NYU2	UDP-glucose:glycoprotein glucosyltransferase 1	UGGT1	6	1.0147
Q6PIU2	Neutral cholesterol ester hydrolase 1	NCEH1	10.5	1.015
P63104	14-3-3 protein zeta/delta	YWHAZ	56.7	1.0151
P00338	L-lactate dehydrogenase A chain	LDHA	57.5	1.0154
P61224;A6NIZ1	Ras-related protein Rap-1b;Ras-related protein Rap-1b-like protein	RAP1B	27.2	1.0156
Q16630	Cleavage and polyadenylation specificity factor subunit 6	CPSF6	4.5	1.016
O95571	Protein ETHE1, mitochondrial	ETHE1	19.3	1.0161
P08473	Neprilysin	MME	8.1	1.0169
P61019;Q8WUD1	Ras-related protein Rab-2A;Ras-related protein Rab-2B	RAB2A;RAB2B	12.3	1.0177
Q08211	ATP-dependent RNA helicase A	DHX9	10.4	1.0178
O75369	Filamin-B	FLNB	23.2	1.0181
P20962	Parathymosin	PTMS	21.6	1.0183
Q7KZF4	Staphylococcal nuclease domain-containing protein 1	SND1	17.9	1.0184
P61353	60S ribosomal protein L27	RPL27	14.7	1.0186
Q9UHD8	Septin-9	Sep-09	14.3	1.0195
P18621	60S ribosomal protein L17	RPL17	41.8	1.0201
Q8WZ42	Titin	TTN	0	1.0204
Q9Y265	RuvB-like 1	RUVBL1	16.4	1.0206
P31930	Cytochrome b-c1 complex subunit 1, mitochondrial	UQCRC1	13.1	1.0214
Q14203	Dynactin subunit 1	DCTN1	3.9	1.023
Q9Y3I0	tRNA-splicing ligase RtcB homolog	C22orf28	11.3	1.0235
O95373;O15397	Importin-7	IPO7	12.7	1.0237
Q13409	Cytoplasmic dynein 1 intermediate chain 2	DYNC1I2	8.6	1.0248
P62906	60S ribosomal protein L10a	RPL10A	35.9	1.0252
Q96FW1	Ubiquitin thioesterase OTUB1	OTUB1	14.4	1.0257
P62826	GTP-binding nuclear protein Ran	RAN	31	1.0261
P62263	40S ribosomal protein S14	RPS14	13.9	1.0264

P15170;Q8IYD1	Eukaryotic peptide chain release factor GTP-binding subunit ERF3A	GSPT1	28.1	1.0267
Q9Y383;Q9NQ29	Putative RNA-binding protein Luc7-like 2;Putative RNA-binding protein Luc7-like 1	LUC7L2;LUC7L	6.9	1.0267
Q9NQR4	Omega-amidase NIT2	NIT2	11.6	1.0273
O75534	Cold shock domain-containing protein E1	CSDE1	7.3	1.0274
P67809;Q9Y2T7	Nuclease-sensitive element-binding protein 1	YBX1	47.5	1.0284
Q13151	Heterogeneous nuclear ribonucleoprotein A0	HNRNPA0	19.3	1.0294
P13489	Ribonuclease inhibitor	RNH1	28.2	1.0297
Q96AY3	Peptidyl-prolyl cis-trans isomerase FKBP10	FKBP10	8.8	1.0299
P48059	LIM and senescent cell antigen-like-containing domain protein 1	LIMS1	14.2	1.0301
Q12906	Interleukin enhancer-binding factor 3	ILF3	8.4	1.0301
P61978	Heterogeneous nuclear ribonucleoprotein K	HNRNPK	30.2	1.0312
Q14108	Lysosome membrane protein 2	SCARB2	6.3	1.0323
P61026	Ras-related protein Rab-10	RAB10	11.5	1.0327
Q8WX93;Q86TC9	Palladin	PALLD	6.4	1.0328
P13473	Lysosome-associated membrane glycoprotein 2	LAMP2	4.1	1.0336
P07108	Acyl-CoA-binding protein	DBI	42.5	1.0345
P38117	Electron transfer flavoprotein subunit beta	ETFB	7.1	1.0347
P51148	Ras-related protein Rab-5C	RAB5C	31.5	1.0359
Q9NP72	Ras-related protein Rab-18	RAB18	26.7	1.0361
Q8NC51	Plasminogen activator inhibitor 1 RNA-binding protein	SERBP1	8.6	1.0373
P07900;Q58FG0;Q14568;Q58FF6;Q58FG1	Heat shock protein HSP 90-alpha	HSP90AA1	23.2	1.0382
P40939	Trifunctional enzyme subunit alpha, mitochondrial;Long-chain enoyl-CoA hydratase;Long chain 3-hydroxyacyl-CoA dehydrogenase	HADHA	22.7	1.0383
P54578	Ubiquitin carboxyl-terminal hydrolase 14	USP14	24.5	1.0395
O75822	Eukaryotic translation initiation factor 3 subunit J	EIF3J	12.8	1.0396
P62877	E3 ubiquitin-protein ligase RBX1	RBX1	17.6	1.0398
P60903	Protein S100-A10	S100A10	35.1	1.0399

O60506	Heterogeneous nuclear ribonucleoprotein Q	SYNCRIP	20.2	1.0404
P51659	Peroxisomal multifunctional enzyme type 2;(3R)-hydroxyacyl-CoA dehydrogenase;Enoyl-CoA hydratase 2	HSD17B4	10.1	1.0405
P16949	Stathmin	STMN1	28.9	1.0408
P83731	60S ribosomal protein L24	RPL24	21	1.0408
Q9Y678;Q9UBF2	Coatomer subunit gamma-1	COPG1	24.7	1.0414
P45974;Q92995	Ubiquitin carboxyl-terminal hydrolase 5	USP5	6.5	1.042
P31949	Protein S100-A11	S100A11	43.8	1.0423
P36957	Dihydrolipoyllysine-residue succinyltransferase component of 2-oxoglutarate dehydrogenase complex, mitochondrial	DLST	9.5	1.043
Q96AE4	Far upstream element-binding protein 1	FUBP1	13.5	1.043
P61513;A6NKH3	60S ribosomal protein L37a	RPL37A	41.3	1.0431
O60716	Catenin delta-1	CTNND1	5.9	1.0436
P62249	40S ribosomal protein S16	RPS16	12.3	1.0438
P61158;Q9P1U1	Actin-related protein 3	ACTR3	38.5	1.0452
Q99584	Protein S100-A13	S100A13	32.7	1.0452
Q9UI12	V-type proton ATPase subunit H	ATP6V1H	12.4	1.0453
Q16643	Drebrin	DBN1	5.9	1.0461
P01111;P01112;P01116	GTPase NRas;GTPase HRas;GTPase HRas, N-terminally processed;GTPase KRas;GTPase KRas, N-terminally processed	NRAS;HRAS;KRAS	13.8	1.0464
O60784	Target of Myb protein 1	TOM1	5.9	1.0466
P07996;P35442;P49746	Thrombospondin-1	THBS1	16.2	1.0483
P20810	Calpastatin	CAST	7.1	1.0483
O15511;Q9BPX5	Actin-related protein 2/3 complex subunit 5;Actin-related protein 2/3 complex subunit 5-like protein	ARPC5;ARPC5L	13.9	1.0488
P27635;Q96L21	60S ribosomal protein L10;60S ribosomal protein L10-like	RPL10;RPL10L	13.1	1.0491
P46778	60S ribosomal protein L21	RPL21	25	1.0493
P06753	Tropomyosin alpha-3 chain	TPM3	36.6	1.0505

Q9UL46	Proteasome activator complex subunit 2	PSME2	24.3	1.0505
Q07954	Prolow-density lipoprotein receptor-related protein 1;Low-density lipoprotein receptor-related protein 1 85 kDa subunit;Low-density lipoprotein receptor-related protein 1 515 kDa subunit;Low-density lipoprotein receptor-related protein 1 intracellular domain	LRP1	0.7	1.0508
P54136	Arginine--tRNA ligase, cytoplasmic	RARS	17.4	1.0509
Q15084	Protein disulfide-isomerase A6	PDIA6	13.6	1.0513
P04899;P09471;Q5JWF2;P63092;P38405;A8MTJ3;P19087;P11488	Guanine nucleotide-binding protein G(i) subunit alpha-2	GNAI2	17.7	1.0517
Q06323	Proteasome activator complex subunit 1	PSME1	15.7	1.0529
Q9HDC9	Adipocyte plasma membrane-associated protein	APMAP	5.3	1.053
P62701;P22090	40S ribosomal protein S4, X isoform	RPS4X	18.3	1.0541
P62888	60S ribosomal protein L30	RPL30	27	1.0547
P52788	Spermine synthase	SMS	21.3	1.055
Q27J81	Inverted formin-2	INF2	6.6	1.0552
P35637	RNA-binding protein FUS	FUS	7.4	1.0556
Q9UKY7	Protein CDV3 homolog	CDV3	18.6	1.056
P15311	Ezrin	EZR	30.7	1.0573
P25445	Tumor necrosis factor receptor superfamily member 6	FAS	10.7	1.0575
P35241	Radixin	RDX	18.2	1.0576
P10620	Microsomal glutathione S-transferase 1	MGST1	20	1.0578
P41091;Q2VIR3	Eukaryotic translation initiation factor 2 subunit 3;Putative eukaryotic translation initiation factor 2 subunit 3-like protein	EIF2S3;EIF2S3L	14.2	1.0585
Q9Y262	Eukaryotic translation initiation factor 3 subunit L	EIF3L	9.9	1.0587
O43583	Density-regulated protein	DENR	13.1	1.0596
P16615;O14983;Q93084	Sarcoplasmic/endoplasmic reticulum calcium ATPase 2;Sarcoplasmic/endoplasmic reticulum calcium ATPase 1	ATP2A2;ATP2A1	10.6	1.0603
P35221	Catenin alpha-1	CTNNA1	14.3	1.0603

Q2TAA2	Isoamyl acetate-hydrolyzing esterase 1 homolog	IAH1	15.7	1.0614
P62910	60S ribosomal protein L32	RPL32	28.1	1.062
O94905	Erlin-2	ERLIN2	26	1.0634
Q13561	Dynactin subunit 2	DCTN2	24.7	1.0634
Q6P2Q9	Pre-mRNA-processing-splicing factor 8	PRPF8	2	1.0638
Q15436	Protein transport protein Sec23A	SEC23A	17.8	1.0645
O75083	WD repeat-containing protein 1	WDR1	49.7	1.0648
P11940;Q9H361;Q4VXU2;Q96DU9	Polyadenylate-binding protein 1;Polyadenylate-binding protein 3	PABPC1;PABPC3	18.7	1.0651
P46821	Microtubule-associated protein 1B;MAP1 light chain LC1	MAP1B	2.6	1.0664
P30520	Adenylosuccinate synthetase isozyme 2	ADSS	4.4	1.0669
P62847	40S ribosomal protein S24	RPS24	19.5	1.0673
Q15046	Lysine--tRNA ligase	KARS	3.9	1.0683
Q9NSD9	Phenylalanine--tRNA ligase beta subunit	FARSB	5.3	1.0684
P48444	Coatomer subunit delta	ARCN1	21.1	1.0703
Q9Y6C9	Mitochondrial carrier homolog 2	MTCH2	14.9	1.0703
Q9P0L0	Vesicle-associated membrane protein-associated protein A	VAPA	25.3	1.0708
Q12905	Interleukin enhancer-binding factor 2	ILF2	15.6	1.0709
P62829	60S ribosomal protein L23	RPL23	37.9	1.0732
O43242	26S proteasome non-ATPase regulatory subunit 3	PSMD3	13.9	1.0765
P62158;P27482	Calmodulin	CALM1	34.9	1.0773
Q9H0U4;Q92928	Ras-related protein Rab-1B;Putative Ras-related protein Rab-1C	RAB1B;RAB1C	36.8	1.0775
P20290	Transcription factor BTF3	BTF3	34.5	1.0779
P39019	40S ribosomal protein S19	RPS19	24.8	1.0779
P99999	Cytochrome c	CYCS	30.5	1.0782
P55735	Protein SEC13 homolog	SEC13	26.7	1.0794
P21964	Catechol O-methyltransferase	COMT	16.6	1.0819
P46977	Dolichyl-diphosphooligosaccharide--protein glycosyltransferase subunit STT3A	STT3A	6	1.0824
P48735	Isocitrate dehydrogenase [NADP], mitochondrial	IDH2	8.2	1.0836
Q16531	DNA damage-binding protein 1	DDB1	1.6	1.0836

O15145	Actin-related protein 2/3 complex subunit 3	ARPC3	26.4	1.0837
Q14247	Src substrate cortactin	CTTN	11.1	1.0848
O14976	Cyclin-G-associated kinase	GAK	1.9	1.0863
P62333	26S protease regulatory subunit 10B	PSMC6	13.1	1.0869
Q00688	Peptidyl-prolyl cis-trans isomerase FKBP3	FKBP3	22.8	1.088
Q96G03	Phosphoglucomutase-2	PGM2	16.3	1.0881
P15144	Aminopeptidase N	ANPEP	13.4	1.0885
O43399	Tumor protein D54	TPD52L2	23.3	1.0892
Q70UQ0	Inhibitor of nuclear factor kappa-B kinase-interacting protein	IKBIP	9.7	1.0895
Q15435	Protein phosphatase 1 regulatory subunit 7	PPP1R7	10.3	1.0902
Q9UBQ5	Eukaryotic translation initiation factor 3 subunit K	EIF3K	25.7	1.0916
P10768	S-formylglutathione hydrolase	ESD	18.1	1.0919
Q9NP97;Q8TF09	Dynein light chain roadblock-type 1;Dynein light chain roadblock-type 2	DYNLRB1;DYNLRB2	30.2	1.0928
P18464;P30491;P30498;P30685;Q95604;P30504;P30508;P30510;P04222;P30501;Q07000;P10319;P30490;Q29940;P30499;P30505;Q29865;Q29963;Q9TNN7;P30460	HLA class I histocompatibility antigen, B-51 alpha chain;HLA class I histocompatibility antigen, B-53 alpha chain;HLA class I histocompatibility antigen, B-78 alpha chain;HLA class I histocompatibility antigen, B-35 alpha chain;HLA class I histocompatibility antigen, Cw-17 alpha chain;HLA class I histocompatibility antigen, Cw-4 alpha chain;HLA class I histocompatibility antigen, Cw-12 alpha chain;HLA class I histocompatibility antigen, Cw-14 alpha chain;HLA class I histocompatibility antigen, Cw-3 alpha chain;HLA class I histocompatibility antigen, Cw-2 alpha chain;HLA class I histocompatibility antigen, Cw-15 alpha chain;HLA class I histocompatibility antigen, B-58 alpha chain;HLA class I histocompatibility antigen, B-52 alpha chain;HLA class I histocompatibility antigen, B-59 alpha chain	HLA-B;HLA-C	13.8	1.093
Q16851	UTP--glucose-1-phosphate uridylyltransferase	UGP2	10.4	1.0946
Q9UNM6	26S proteasome non-ATPase regulatory subunit 13	PSMD13	22.1	1.0946
Q53GQ0	Estradiol 17-beta-dehydrogenase 12	HSD17B12	24.7	1.0947
P62854;Q5JNZ5	40S ribosomal protein S26;Putative 40S ribosomal protein S26-like 1	RPS26;RPS26P11	23.5	1.0956
O94979	Protein transport protein Sec31A	SEC31A	9.1	1.0959

P11279	Lysosome-associated membrane glycoprotein 1	LAMP1	6.5	1.096
Q15287	RNA-binding protein with serine-rich domain 1	RNPS1	8.9	1.0967
O43852	Calumenin	CALU	38.1	1.0971
P61163	Alpha-centractin	ACTR1A	26.6	1.0976
Q9ULV4	Coronin-1C	CORO1C	27.2	1.0986
P07942	Laminin subunit beta-1	LAMB1	3.8	1.0993
Q9Y281	Cofilin-2	CFL2	57.2	1.0993
Q92882	Osteoclast-stimulating factor 1	OSTF1	14.5	1.0995
Q86VP6;O75155	Cullin-associated NEDD8-dissociated protein 1	CAND1	8.3	1.1
Q8TAT6	Nuclear protein localization protein 4 homolog	NPLOC4	7.6	1.1002
Q16543	Hsp90 co-chaperone Cdc37	CDC37	8.7	1.1013
Q07866;Q9H0B6	Kinesin light chain 1	KLC1	18	1.1018
P46776	60S ribosomal protein L27a	RPL27A	21.6	1.102
P30050	60S ribosomal protein L12	RPL12	24.2	1.1029
Q14974	Importin subunit beta-1	KPNB1	17.2	1.1043
Q14195;Q14194	Dihydropyrimidinase-related protein 3	DPYSL3	23.5	1.1044
Q14764	Major vault protein	MVP	22.6	1.1045
Q8N1G4	Leucine-rich repeat-containing protein 47	LRRC47	3.6	1.1047
Q07020	60S ribosomal protein L18	RPL18	26.1	1.1065
P17844	Probable ATP-dependent RNA helicase DDX5	DDX5	14.5	1.1078
Q12797	Aspartyl/asparaginyl beta-hydroxylase	ASPH	13.3	1.1093
P61160	Actin-related protein 2	ACTR2	15.2	1.1099
P62873;P16520	Guanine nucleotide-binding protein G(I)/G(S)/G(T) subunit beta-1	GNB1	34.4	1.1105
P08708;P0CW22	40S ribosomal protein S17;40S ribosomal protein S17-like	RPS17;RPS17L	48.1	1.1131
Q14152	Eukaryotic translation initiation factor 3 subunit A	EIF3A	6.9	1.1142
P32321	Deoxycytidylate deaminase	DCTD	19.1	1.1149
P61204;P84077	ADP-ribosylation factor 3;ADP-ribosylation factor 1	ARF3;ARF1	43.6	1.115
P51572	B-cell receptor-associated protein 31	BCAP31	13.4	1.1153
Q16222	UDP-N-acetylhexosamine pyrophosphorylase;UDP-N-acetylgalactosamine pyrophosphorylase;UDP-N-acetylglucosamine	UAP1	8.6	1.1155

	pyrophosphorylase			
P23246	Splicing factor, proline- and glutamine-rich	SFPQ	6.5	1.1157
P55010	Eukaryotic translation initiation factor 5	EIF5	7.9	1.116
P62269	40S ribosomal protein S18	RPS18	19.1	1.116
Q13148	TAR DNA-binding protein 43	TARDBP	8.7	1.1177
Q02952	A-kinase anchor protein 12	AKAP12	7.9	1.1184
Q15942	Zyxin	ZYX	22	1.1191
P23396	40S ribosomal protein S3	RPS3	33.7	1.1198
P41252	Isoleucine--tRNA ligase, cytoplasmic	IARS	11.9	1.1201
Q13637;P57729;O14966	Ras-related protein Rab-32;Ras-related protein Rab-38;Ras-related protein Rab-7L1	RAB32;RAB38;RAB7L1	11.1	1.1211
P02545	Prelamin-A/C;Lamin-A/C	LMNA	26.4	1.1221
Q00610;P53675	Clathrin heavy chain 1	CLTC	27.4	1.1233
P62913	60S ribosomal protein L11	RPL11	16.9	1.1234
P21926	CD9 antigen	CD9	20.2	1.1235
Q16555	Dihydropyrimidinase-related protein 2	DPYSL2	32	1.1237
Q7L2H7	Eukaryotic translation initiation factor 3 subunit M	EIF3M	13.1	1.1241
P07814	Bifunctional glutamate/proline--tRNA ligase;Glutamate--tRNA ligase;Proline--tRNA ligase	EPRS	12	1.1247
P11047	Laminin subunit gamma-1	LAMC1	2.6	1.1253
P20337	Ras-related protein Rab-3B	RAB3B	21	1.1258
P46782	40S ribosomal protein S5;40S ribosomal protein S5, N-terminally processed	RPS5	13.7	1.126
Q96AG4	Leucine-rich repeat-containing protein 59	LRRC59	34.5	1.1265
Q9BUF5	Tubulin beta-6 chain	TUBB6	33.6	1.1277
P19367	Hexokinase-1	HK1	2.9	1.1285
P26583	High mobility group protein B2	HMGB2	20.1	1.1302
P61106	Ras-related protein Rab-14	RAB14	20.9	1.1305
P63167;Q96FJ2	Dynein light chain 1, cytoplasmic;Dynein light chain 2, cytoplasmic	DYNLL1;DYNLL2	37.1	1.1306
O75955	Flotillin-1	FLOT1	13.3	1.1313
Q9Y3B3	Transmembrane emp24 domain-containing protein 7	TMED7	16.1	1.1319
Q7Z2W4	Zinc finger CCCH-type antiviral protein 1	ZC3HAV1	5.2	1.1323

Q02218	2-oxoglutarate dehydrogenase, mitochondrial	OGDH	3.8	1.134
O15143	Actin-related protein 2/3 complex subunit 1B	ARPC1B	27.2	1.1342
P68104;Q5VTE0;Q05639	Elongation factor 1-alpha 1;Putative elongation factor 1-alpha-like 3;Elongation factor 1-alpha 2	EEF1A1;EEF1A1P5;EEF1A2	55.6	1.1342
Q99613	Eukaryotic translation initiation factor 3 subunit C	EIF3C	5.8	1.1358
P08133	Annexin A6	ANXA6	28.7	1.1365
P26038	Moesin	MSN	36.2	1.1376
Q15008	26S proteasome non-ATPase regulatory subunit 6	PSMD6	6.2	1.1406
Q01518	Adenylyl cyclase-associated protein 1	CAP1	45.5	1.1409
Q9BWM7	Sideroflexin-3	SFXN3	9.2	1.1409
P62244	40S ribosomal protein S15a	RPS15A	13.8	1.1429
Q04637;O43432	Eukaryotic translation initiation factor 4 gamma 1;Eukaryotic translation initiation factor 4 gamma 3	EIF4G1;EIF4G3	1.6	1.1464
O00159	Unconventional myosin-Ic	MYO1C	16.5	1.1476
P62820	Ras-related protein Rab-1A	RAB1A	38.5	1.1479
P78527	DNA-dependent protein kinase catalytic subunit	PRKDC	4.5	1.1483
Q8WUM4	Programmed cell death 6-interacting protein	PDCD6IP	12.8	1.1502
P55084	Trifunctional enzyme subunit beta, mitochondrial;3-ketoacyl-CoA thiolase	HADHB	25.5	1.1504
Q13177	Serine/threonine-protein kinase PAK 2;PAK-2p27;PAK-2p34	PAK2	7.1	1.1526
Q99460	26S proteasome non-ATPase regulatory subunit 1	PSMD1	10.5	1.1533
Q07065	Cytoskeleton-associated protein 4	CKAP4	26.2	1.1543
P68402	Platelet-activating factor acetylhydrolase IB subunit beta	PAFAH1B2	8.7	1.1548
Q7L576	Cytoplasmic FMR1-interacting protein 1	CYFIP1	2.1	1.158
P43243	Matrin-3	MATR3	15.8	1.1599
Q15691	Microtubule-associated protein RP/EB family member 1	MAPRE1	10.4	1.1599
Q15121	Astrocytic phosphoprotein PEA-15	PEA15	34.6	1.1601
P67936;Q2TAC2	Tropomyosin alpha-4 chain	TPM4	44.8	1.161
P40429;Q6NVV1	60S ribosomal protein L13a;Putative 60S ribosomal protein L13a-like MGC87657	RPL13A	18.2	1.1611
P62081	40S ribosomal protein S7	RPS7	48.5	1.1611
P52306	Rap1 GTPase-GDP dissociation stimulator 1	RAP1GDS1	7.9	1.1614

P46781	40S ribosomal protein S9	RPS9	21.6	1.1624
P17655	Calpain-2 catalytic subunit	CAPN2	12.1	1.1641
P15374	Ubiquitin carboxyl-terminal hydrolase isozyme L3	UCHL3	25.7	1.1655
P49748	Very long-chain specific acyl-CoA dehydrogenase, mitochondrial	ACADVL	14.5	1.1687
A0AVT1	Ubiquitin-like modifier-activating enzyme 6	UBA6	4.1	1.1689
O43707	Alpha-actinin-4	ACTN4	38.2	1.169
O60664	Perilipin-3	PLIN3	33.4	1.1695
Q9Y696	Chloride intracellular channel protein 4	CLIC4	52.2	1.1701
P02452;CON__Q862S4	Collagen alpha-1(I) chain	COL1A1	9.4	1.1725
P05556	Integrin beta-1	ITGB1	26.4	1.1725
Q9P2E9	Ribosome-binding protein 1	RRBP1	16.6	1.1727
O75506	Heat shock factor-binding protein 1	HSBP1	59.2	1.1731
P15880	40S ribosomal protein S2	RPS2	34.5	1.1741
P21281	V-type proton ATPase subunit B, brain isoform	ATP6V1B2	5.1	1.1762
Q9NYL9	Tropomodulin-3	TMOD3	21.9	1.1765
P52292	Importin subunit alpha-2	KPNA2	11.5	1.1771
P35754	Glutaredoxin-1	GLRX	17.9	1.1783
Q10567	AP-1 complex subunit beta-1	AP1B1	10	1.1798
Q9BW60	Elongation of very long chain fatty acids protein 1	ELOVL1	9	1.1803
Q8N766	Uncharacterized protein KIAA0090	KIAA0090	3	1.1809
Q15075	Early endosome antigen 1	EEA1	2.6	1.1811
Q9UBQ7	Glyoxylate reductase/hydroxypyruvate reductase	GRHPR	15.2	1.1817
Q9UPN3;Q03001	Microtubule-actin cross-linking factor 1, isoforms 1/2/3/5	MACF1	1.6	1.1818
O60763	General vesicular transport factor p115	USO1	2.5	1.1827
Q92900	Regulator of nonsense transcripts 1	UPF1	3.2	1.184
P48163	NADP-dependent malic enzyme	ME1	5.2	1.1853
P27824	Calnexin	CANX	18.8	1.1878
Q92499	ATP-dependent RNA helicase DDX1	DDX1	9.5	1.1881
Q8TCJ2	Dolichyl-diphosphooligosaccharide--protein glycosyltransferase subunit STT3B	STT3B	3.8	1.189
Q93052	Lipoma-preferred partner	LPP	15.8	1.1898
Q8NBJ5	Procollagen galactosyltransferase 1	GLT25D1	4.5	1.1905
P16070	CD44 antigen	CD44	3.6	1.1925

P00387	NADH-cytochrome b5 reductase 3;NADH-cytochrome b5 reductase 3 membrane-bound form;NADH-cytochrome b5 reductase 3 soluble form	CYB5R3	15.6	1.1927
Q99536	Synaptic vesicle membrane protein VAT-1 homolog	VAT1	8.9	1.193
P07355;A6NMY6	Annexin A2;Putative annexin A2-like protein	ANXA2;ANXA2P2	44.8	1.1931
Q14204	Cytoplasmic dynein 1 heavy chain 1	DYNC1H1	9.7	1.1934
P60953;P17081;Q9H4E5;P15153;P60763;P84095	Cell division control protein 42 homolog;Rho-related GTP-binding protein RhoQ	CDC42;RHOQ	25.7	1.1948
P61088;Q5JXB2	Ubiquitin-conjugating enzyme E2 N;Putative ubiquitin-conjugating enzyme E2 N-like	UBE2N;UBE2NL	16.4	1.1953
P62280	40S ribosomal protein S11	RPS11	16.5	1.1958
O95817	BAG family molecular chaperone regulator 3	BAG3	12.5	1.1959
Q15041	ADP-ribosylation factor-like protein 6-interacting protein 1	ARL6IP1	4.9	1.1967
Q9BQG0	Myb-binding protein 1A	MYBBP1A	2.5	1.2
Q9Y4K0	Lysyl oxidase homolog 2	LOXL2	8.5	1.2005
O15371	Eukaryotic translation initiation factor 3 subunit D	EIF3D	14.6	1.2006
Q15907;P62491;P57735	Ras-related protein Rab-11B;Ras-related protein Rab-11A	RAB11B;RAB11A	24.3	1.2008
O15460	Prolyl 4-hydroxylase subunit alpha-2	P4HA2	13.8	1.2033
Q14192	Four and a half LIM domains protein 2	FHL2	57.3	1.2042
O15144	Actin-related protein 2/3 complex subunit 2	ARPC2	34.3	1.2043
Q16795	NADH dehydrogenase [ubiquinone] 1 alpha subcomplex subunit 9, mitochondrial	NDUFA9	7.7	1.2045
P30085	UMP-CMP kinase	CMPK1	35.7	1.2061
P51452	Dual specificity protein phosphatase 3	DUSP3	13.5	1.2065
Q9UHI8	A disintegrin and metalloproteinase with thrombospondin motifs 1	ADAMTS1	7.2	1.2071
P42677	40S ribosomal protein S27	RPS27	28.6	1.2075
P78344	Eukaryotic translation initiation factor 4 gamma 2	EIF4G2	4.9	1.2075
Q07666	KH domain-containing, RNA-binding, signal transduction-associated protein 1	KHDRBS1	4.5	1.208

P07384	Calpain-1 catalytic subunit	CAPN1	3.5	1.2101
P04632;Q96L46	Calpain small subunit 1	CAPNS1	17.9	1.2102
P28331	NADH-ubiquinone oxidoreductase 75 kDa subunit, mitochondrial	NDUFS1	11.3	1.2126
Q01082;Q9H254;O15020;P11277	Spectrin beta chain, brain 1	SPTBN1	6.9	1.2135
P63173	60S ribosomal protein L38	RPL38	24.3	1.2138
O00629	Importin subunit alpha-4	KPNA4	17.3	1.214
Q9BUJ2	Heterogeneous nuclear ribonucleoprotein U-like protein 1	HNRNPUL1	7.5	1.2142
P62851	40S ribosomal protein S25	RPS25	14.4	1.2171
P62495	Eukaryotic peptide chain release factor subunit 1	ETF1	16.2	1.2176
O14980	Exportin-1	XPO1	5.4	1.2188
P46940;Q86VI3;Q13576	Ras GTPase-activating-like protein IQGAP1	IQGAP1	23.2	1.2192
P45877	Peptidyl-prolyl cis-trans isomerase C	PPIC	14.6	1.2207
O95202	LETM1 and EF-hand domain-containing protein 1, mitochondrial	LETM1	4.9	1.2235
P07910;O60812	Heterogeneous nuclear ribonucleoproteins C1/C2;Heterogeneous nuclear ribonucleoprotein C-like 1	HNRNPC;HNRNPCL1	14.7	1.2256
P07602	Proactivator polypeptide;Saposin-A;Saposin-B-Val;Saposin-B;Saposin-C;Saposin-D	PSAP	12.8	1.2262
Q8IWE2	Protein NOXP20	FAM114A1	12.8	1.227
Q00341	Vigilin	HDLBP	12.4	1.2274
P20340;Q9NRW1	Ras-related protein Rab-6A;Ras-related protein Rab-6B	RAB6A;RAB6B	16.8	1.2279
Q9H3N1	Thioredoxin-related transmembrane protein 1	TMX1	8.2	1.2282
P38571	Lysosomal acid lipase/cholesteryl ester hydrolase	LIPA	10.3	1.2296
P32969	60S ribosomal protein L9	RPL9	18.2	1.2301
P08123	Collagen alpha-2(I) chain	COL1A2	6	1.2355
P20073	Annexin A7	ANXA7	4.1	1.2359
P62277	40S ribosomal protein S13	RPS13	17.9	1.2361
P28161	Glutathione S-transferase Mu 2	GSTM2	14.2	1.2377

O95573	Long-chain-fatty-acid--CoA ligase 3	ACSL3	7.2	1.2399
P33176;Q12840	Kinesin-1 heavy chain	KIF5B	18.2	1.2405
P08648	Integrin alpha-5;Integrin alpha-5 heavy chain;Integrin alpha-5 light chain	ITGA5	7.5	1.2407
Q9NVA2;Q92599;Q14141;Q9P0V9	Septin-11	Sep-11	15.4	1.2411
O75828	Carbonyl reductase [NADPH] 3	CBR3	19.5	1.2432
Q09666	Neuroblast differentiation-associated protein AHNAK	AHNAK	45.1	1.2432
O75368	SH3 domain-binding glutamic acid-rich-like protein	SH3BGRL	47.4	1.2435
P63261	Actin, cytoplasmic 2;Actin, cytoplasmic 2, N-terminally processed	ACTG1	65.1	1.2443
P52566	Rho GDP-dissociation inhibitor 2	ARHGDIB	33.8	1.2448
P12111	Collagen alpha-3(VI) chain	COL6A3	11.4	1.2464
Q7L5N1	COP9 signalosome complex subunit 6	COPS6	16.8	1.2466
P17252;P05771	Protein kinase C alpha type;Protein kinase C beta type	PRKCA;PRKCB	8.3	1.2477
P46939	Utrophin	UTRN	1.8	1.2484
Q16836	Hydroxyacyl-coenzyme A dehydrogenase, mitochondrial	HADH	24.2	1.2527
Q86UE4	Protein LYRIC	MTDH	10.1	1.2571
P62273	40S ribosomal protein S29	RPS29	33.9	1.2604
Q9Y3A5	Ribosome maturation protein SBDS	SBDS	10.4	1.2617
P15151	Poliovirus receptor	PVR	8.4	1.2621
Q16181;Q6ZU15	Septin-7	Sep-07	25.2	1.2643
P40121	Macrophage-capping protein	CAPG	9.8	1.2666
Q92616	Translational activator GCN1	GCN1L1	6.4	1.2684
P37802	Transgelin-2	TAGLN2	53.3	1.2685
Q5T4S7	E3 ubiquitin-protein ligase UBR4	UBR4	1.4	1.2685
P12814;Q08043;P35609	Alpha-actinin-1	ACTN1	37.7	1.2695
Q9NQC3	Reticulon-4	RTN4	3.7	1.2718

Q9H4M9;Q9NZN3;Q9H223	EH domain-containing protein 1	EHD1	20	1.2727
P46734;P52564	Dual specificity mitogen-activated protein kinase kinase 3;Dual specificity mitogen-activated protein kinase kinase 6	MAP2K3;MAP2K6	9.2	1.2744
Q13418	Integrin-linked protein kinase	ILK	10.8	1.2761
P27816	Microtubule-associated protein 4	MAP4	20.6	1.2781
Q32MZ4	Leucine-rich repeat flightless-interacting protein 1	LRRFIP1	9.2	1.279
Q9Y490	Talin-1	TLN1	28.7	1.279
P53396	ATP-citrate synthase	ACLY	22.8	1.2798
P83111	Serine beta-lactamase-like protein LACTB, mitochondrial	LACTB	6.6	1.2818
O00571;O15523;Q9NQI0	ATP-dependent RNA helicase DDX3X;ATP-dependent RNA helicase DDX3Y	DDX3X;DDX3Y	21.5	1.2819
P27105	Erythrocyte band 7 integral membrane protein	STOM	16.7	1.2827
Q15019	Septin-2	Sep-02	13.9	1.2827
P63010	AP-2 complex subunit beta	AP2B1	17.9	1.283
Q7Z4H8	KDEL motif-containing protein 2	KDELC2	4.7	1.2839
Q7L1Q6;Q9Y6E2	Basic leucine zipper and W2 domain-containing protein 1;Basic leucine zipper and W2 domain-containing protein 2	BZW1;BZW2	3.6	1.2847
P18124	60S ribosomal protein L7	RPL7	19.4	1.285
Q9NVD7;Q9HBI1	Alpha-parvin	PARVA	15.3	1.2862
P13797;P13796;Q14651	Plastin-3	PLS3	41.3	1.2877
P78559	Microtubule-associated protein 1A;MAP1 light chain LC2	MAP1A	3.6	1.2878
Q15417	Calponin-3	CNN3	37.1	1.291
P50552	Vasodilator-stimulated phosphoprotein	VASP	12.9	1.2912
Q96JJ7	Protein disulfide-isomerase TMX3	TMX3	7	1.2958
Q9BVA6	Adenosine monophosphate-protein transferase FICD	FICD	3.5	1.3044
Q13885;Q9BVA1;A6N	Tubulin beta-2A chain;Tubulin beta-2B chain	TUBB2A;TUBB2B	24.7	1.3068

NZ2				
P11310	Medium-chain specific acyl-CoA dehydrogenase, mitochondrial	ACADM	6.4	1.3101
O76021	Ribosomal L1 domain-containing protein 1	RSL1D1	15.1	1.311
Q13813	Spectrin alpha chain, brain	SPTAN1	5.9	1.3162
Q13492;O60641	Phosphatidylinositol-binding clathrin assembly protein	PICALM	10	1.3165
O14617	AP-3 complex subunit delta-1	AP3D1	3.2	1.3173
P18206	Vinculin	VCL	30.8	1.3192
P68366	Tubulin alpha-4A chain	TUBA4A	39.5	1.3208
Q13442	28 kDa heat- and acid-stable phosphoprotein	PDAP1	13.8	1.3211
O75367;Q9P0M6	Core histone macro-H2A.1	H2AFY	16.1	1.3233
O14558	Heat shock protein beta-6	HSPB6	38.1	1.3236
P05204	Non-histone chromosomal protein HMG-17	HMGN2	28.9	1.3323
O00469	Procollagen-lysine,2-oxoglutarate 5-dioxygenase 2	PLOD2	13.2	1.3347
Q99439	Calponin-2	CNN2	30.4	1.338
P04792	Heat shock protein beta-1	HSPB1	27.8	1.341
Q13907	Isopentenyl-diphosphate Delta-isomerase 1	IDI1	14.5	1.3431
P30740;O75830;P50453;P50452	Leukocyte elastase inhibitor	SERPINB1	9	1.3471
Q16270	Insulin-like growth factor-binding protein 7	IGFBP7	10.6	1.3485
P54727;P54725	UV excision repair protein RAD23 homolog B	RAD23B	9.3	1.3486
O43795;Q9UBC5	Unconventional myosin-Ib	MYO1B	5.3	1.3515
Q9BSJ8	Extended synaptotagmin-1	ESYT1	10.6	1.3524
Q9Y570	Protein phosphatase methylesterase 1	PPME1	25.9	1.3535
P60981	Destrin	DSTN	58.8	1.3579
P08572	Collagen alpha-2(IV) chain;Canstatin	COL4A2	1.5	1.358
Q9NR12	PDZ and LIM domain protein 7	PDLIM7	28.9	1.3583
Q15149;P58107	Plectin	PLEC	16.8	1.3608
Q86Y82	Syntaxin-12	STX12	18.5	1.3623
Q9NZM1;O75923	Myoferlin	MYOF	12.4	1.3689

Q15404	Ras suppressor protein 1	RSU1	28.9	1.3701
P08134	Rho-related GTP-binding protein RhoC	RHOC	31.1	1.3756
P60660;P14649	Myosin light polypeptide 6	MYL6	38.4	1.3779
P30837	Aldehyde dehydrogenase X, mitochondrial	ALDH1B1	6.6	1.3829
O94973	AP-2 complex subunit alpha-2	AP2A2	6.9	1.39
Q12792	Twinfilin-1	TWF1	13.7	1.3916
Q9Y6G9	Cytoplasmic dynein 1 light intermediate chain 1	DYNC1LI1	17.2	1.3922
P68032;P68133;P62736;P63267	Actin, alpha cardiac muscle 1;Actin, alpha skeletal muscle;Actin, aortic smooth muscle;Actin, gamma-enteric smooth muscle	ACTC1;ACTA1;ACTA2;ACTG2	49.9	1.3994
P54652	Heat shock-related 70 kDa protein 2	HSPA2	13.5	1.4007
Q96HC4	PDZ and LIM domain protein 5	PDLIM5	21	1.4048
Q07960	Rho GTPase-activating protein 1	ARHGAP1	14.6	1.4137
Q00325	Phosphate carrier protein, mitochondrial	SLC25A3	6.9	1.4144
O00410;O60518	Importin-5	IPO5	30.9	1.4164
Q14847	LIM and SH3 domain protein 1	LASP1	21.1	1.4185
O95782	AP-2 complex subunit alpha-1	AP2A1	11.3	1.4196
P46459	Vesicle-fusing ATPase	NSF	2.4	1.4227
P10301;P62070	Ras-related protein R-Ras;Ras-related protein R-Ras2	RRAS;RRAS2	11.5	1.4273
O94925	Glutaminase kidney isoform, mitochondrial	GLS	16.1	1.4295
P08670;P17661	Vimentin	VIM	44.2	1.4346
P63172	Dynein light chain Tctex-type 1	DYNLT1	15.9	1.4401
O00232	26S proteasome non-ATPase regulatory subunit 12	PSMD12	7	1.4405
P61764	Syntaxin-binding protein 1	STXBP1	6.4	1.443
P42574	Caspase-3;Caspase-3 subunit p17;Caspase-3 subunit p12	CASP3	9	1.4491
P49821	NADH dehydrogenase [ubiquinone] flavoprotein 1, mitochondrial	NDUFV1	8	1.4517
O14974	Protein phosphatase 1 regulatory subunit 12A	PPP1R12A	3.8	1.4531
P10644;P31321	cAMP-dependent protein kinase type I-alpha regulatory subunit;cAMP-dependent protein kinase type I-beta regulatory subunit	PRKAR1A;PRKAR1B	4.2	1.4556
Q9BWD1	Acetyl-CoA acetyltransferase, cytosolic	ACAT2	41.8	1.4574
Q9NRV9	Heme-binding protein 1	HEBP1	21.2	1.4646

P23634;P20020;Q16720;Q01814	Plasma membrane calcium-transporting ATPase 4	ATP2B4	8.5	1.4685
P21333	Filamin-A	FLNA	37.1	1.477
Q96N66	Lysophospholipid acyltransferase 7	MBOAT7	8.7	1.4781
Q9UBI6	Guanine nucleotide-binding protein G(I)/G(S)/G(O) subunit gamma-12	GNG12	41.7	1.4882
Q0ZGT2	Nexilin	NEXN	15.6	1.4904
Q13045	Protein flightless-1 homolog	FLII	2.4	1.4913
Q5GLZ8	Probable E3 ubiquitin-protein ligase HERC4	HERC4	10.5	1.4961
Q4L180	Filamin A-interacting protein 1-like	FILIP1L	1.9	1.4985
O00151	PDZ and LIM domain protein 1	PDLIM1	37.1	1.5045
P02462	Collagen alpha-1(IV) chain;Arresten	COL4A1	3	1.5049
Q969G5	Protein kinase C delta-binding protein	PRKCDBP	13	1.5113
P41221;Q9H1J7	Protein Wnt-5a;Protein Wnt-5b	WNT5A;WNT5B	6.8	1.5182
Q9UHB6	LIM domain and actin-binding protein 1	LIMA1	15.3	1.5189
Q6DD88	Atlastin-3	ATL3	30.7	1.5208
Q9BQE5	Apolipoprotein L2	APOL2	7.4	1.5294
Q8N8S7	Protein enabled homolog	ENAH	5.6	1.5494
P35579;P35749	Myosin-9	MYH9	41.6	1.5543
Q86UP2	Kinectin	KTN1	4.8	1.5554
P49327	Fatty acid synthase;[Acyl-carrier-protein] S-acetyltransferase;[Acyl-carrier-protein] S-malonyltransferase;3-oxoacyl-[acyl-carrier-protein] synthase;3-oxoacyl-[acyl-carrier-protein] reductase;3-hydroxypalmitoyl-[acyl-carrier-protein] dehydratase;Enoyl-[acyl-carrier-protein] reductase;Oleoyl-[acyl-carrier-protein] hydrolase	FASN	14.2	1.5555
P49593	Protein phosphatase 1F	PPM1F	13.7	1.5672
Q9NZN4	EH domain-containing protein 2	EHD2	16	1.5781
P61587	Rho-related GTP-binding protein RhoE	RND3	10.2	1.5923
Q15746	Myosin light chain kinase, smooth muscle;Myosin light chain kinase, smooth muscle, deglutamylated form	MYLK	5.8	1.5947
Q9Y4F1	FERM, RhoGEF and pleckstrin domain-containing protein 1	FARP1	7	1.5958

P06756	Integrin alpha-V;Integrin alpha-V heavy chain;Integrin alpha-V light chain	ITGAV	12.1	1.6023
O14602;P47813	Eukaryotic translation initiation factor 1A, Y-chromosomal;Eukaryotic translation initiation factor 1A, X-chromosomal	EIF1AY;EIF1AX	18.1	1.6166
O95340	Bifunctional 3-phosphoadenosine 5-phosphosulfate synthase 2;Sulfate adenylyltransferase;Adenylyl-sulfate kinase	PAPSS2	21.2	1.6352
O60488	Long-chain-fatty-acid--CoA ligase 4	ACSL4	5.9	1.644
P04216	Thy-1 membrane glycoprotein	THY1	14.9	1.6477
Q14315	Filamin-C	FLNC	19.6	1.6548
Q9Y2T2	AP-3 complex subunit mu-1	AP3M1	9.8	1.6553
Q9UBG0	C-type mannose receptor 2	MRC2	4	1.6749
P40123	Adenylyl cyclase-associated protein 2	CAP2	20.8	1.6782
P01130	Low-density lipoprotein receptor	LDLR	4.4	1.7166
Q9UHG3	Prenylcysteine oxidase 1	PCYOX1	8.3	1.74
Q96AC1	Fermitin family homolog 2	FERMT2	17.4	1.7521
Q8TAD7	Overexpressed in colon carcinoma 1 protein	OCC1	54	1.7579
P17812;Q9NRF8	CTP synthase 1	CTPS	10.3	1.7616
P98082	Disabled homolog 2	DAB2	22.5	1.773
Q8WUP2	Filamin-binding LIM protein 1	FBLIM1	14.7	1.7768
P30046;A6NHG4	D-dopachrome decarboxylase;D-dopachrome decarboxylase-like protein	DDT;DDTL	19.5	1.7909
Q13557;Q13554;Q13555;Q9UQM7	Calcium/calmodulin-dependent protein kinase type II subunit delta	CAMK2D	20	1.825
P32455;Q9H0R5	Interferon-induced guanylate-binding protein 1;Guanylate-binding protein 3	GBP1;GBP3	4.4	1.8416
O14950;P19105;P24844	Myosin regulatory light chain 12B;Myosin regulatory light chain 12A;Myosin regulatory light polypeptide 9	MYL12B;MYL12A;MYL9	41.9	1.8486
P05783;CON__P05784;CON__Q497I4;Q92764;CON__Q	Keratin, type I cytoskeletal 18	KRT18	26.7	1.869

92764				
Q05682	Caldesmon	CALD1	21.3	1.8706
P13726	Tissue factor	F3	12.9	1.8811
Q03135	Caveolin-1	CAV1	19.1	1.888
P40261	Nicotinamide N-methyltransferase	NNMT	8.3	1.939
Q6NZI2	Polymerase I and transcript release factor	PTRF	11.5	1.9401
P21291	Cysteine and glycine-rich protein 1	CSRP1	64.8	2.0033
Q9UHQ9	NADH-cytochrome b5 reductase 1	CYB5R1	19.3	2.0245
Q6UVK1	Chondroitin sulfate proteoglycan 4	CSPG4	4.9	2.0826
Q01995	Transgelin	TAGLN	65.2	2.0888
P05106	Integrin beta-3	ITGB3	4.4	2.1996
P09493	Tropomyosin alpha-1 chain	TPM1	35.2	2.2465
P02751	Fibronectin;Anastellin;Ugl-Y1;Ugl-Y2;Ugl-Y3	FN1	15.8	2.2833
P07197	Neurofilament medium polypeptide	NEFM	5.1	2.2981
P60891;P21108;P11908	Ribose-phosphate pyrophosphokinase 1	PRPS1	21.7	2.4631
P12109	Collagen alpha-1(VI) chain	COL6A1	10.3	2.6151
Q9UMS6	Synaptopodin-2	SYNPO2	8.6	3.0174
Q13642	Four and a half LIM domains protein 1	FHL1	24.1	3.2279
P51911	Calponin-1	CNN1	64	3.8031
P12110	Collagen alpha-2(VI) chain	COL6A2	5.8	3.9845

[a] Normalized ratio H/L reversed: 1/[H/L].

[b] Sequence coverage %.

[c] Not applicable.

[d] Not detected.

[e] Analyzed by STRING9.1.

Supplementary Table S2

Protein IDs	Protein names	Gene names	Sequence coverage [%]	Ratio OCHA^{L}: OTCS^{H}
P00352	Retinal dehydrogenase 1	ALDH1A1	42.9	0.1859
P13726	Tissue factor	F3	9.2	0.2097
O00622	Protein CYR61	CYR61	35.7	0.3514
Q9UHI8	A disintegrin and metalloproteinase with thrombospondin motifs 1	ADAMTS1	3.9	0.3664
P32004	Neural cell adhesion molecule L1	L1CAM	2	0.3765
Q13642	Four and a half LIM domains protein 1	FHL1	16.1	0.3981
P08195	4F2 cell-surface antigen heavy chain	SLC3A2	10.2	0.4207
P61587	Rho-related GTP-binding protein RhoE	RND3	17.2	0.4502
Q9UHB6	LIM domain and actin-binding protein 1	LIMA1	13	0.4547
P51665	26S proteasome non-ATPase regulatory subunit 7	PSMD7	6.2	0.4660
P60891;P11908;P21108	Ribose-phosphate pyrophosphokinase 1	PRPS1	37.1	0.4747
P07197	Neurofilament medium polypeptide	NEFM	9.4	0.4864
P05783;CON__P05784	Keratin, type I cytoskeletal 18	KRT18	21.4	0.4916
Q13740	CD166 antigen	ALCAM	14.2	0.5095
Q12841	Follistatin-related protein 1	FSTL1	15.6	0.5103
Q13501	Sequestosome-1	SQSTM1	17.3	0.5160
Q9Y570	Protein phosphatase methylesterase 1	PPME1	27.7	0.5231
P01130	Low-density lipoprotein receptor	LDLR	3.5	0.5265
Q16222	UDP-N-acetylhexosamine pyrophosphorylase;UDP-N-acetylgalactosamine pyrophosphorylase;UDP-N-acetylglucosamine pyrophosphorylase	UAP1	14.2	0.5320
Q8TAD7	Overexpressed in colon carcinoma 1 protein	OCC1	34.9	0.5566
P38571	Lysosomal acid lipase/cholesteryl ester hydrolase	LIPA	17.8	0.5689
Q15758	Neutral amino acid transporter B(0)	SLC1A5	3.7	0.5863
Q9NR30;Q9BQ39	Nucleolar RNA helicase 2	DDX21	7.8	0.5883

Q14315	Filamin-C	FLNC	23.4	0.5924
P98082	Disabled homolog 2	DAB2	16.5	0.5926
Q9BZF1	Oxysterol-binding protein-related protein 8	OSBPL8	2.9	0.5974
P15311	Ezrin	EZR	36.9	0.6001
P20337	Ras-related protein Rab-3B	RAB3B	30.1	0.6002
Q96RD7	Pannexin-1	PANX1	7.7	0.6002
P09493	Tropomyosin alpha-1 chain	TPM1	26.1	0.6003
O95340;O43252	Bifunctional 3-phosphoadenosine 5-phosphosulfate synthase 2;Sulfate adenylyltransferase;Adenylyl-sulfate kinase	PAPSS2	29.8	0.6018
Q01995	Transgelin	TAGLN	69.2	0.6132
P08648	Integrin alpha-5;Integrin alpha-5 heavy chain;Integrin alpha-5 light chain	ITGA5	6.7	0.6141
P46734;P52564	Dual specificity mitogen-activated protein kinase kinase 3;Dual specificity mitogen-activated protein kinase kinase 6	MAP2K3;MAP2K6	9.2	0.6174
P29279	Connective tissue growth factor	CTGF	27.2	0.6211
P35520	Cystathionine beta-synthase	CBS	7.8	0.6236
Q16850	Lanosterol 14-alpha demethylase	CYP51A1	7	0.6285
P62070;P10301	Ras-related protein R-Ras2;Ras-related protein R-Ras	RRAS2;RRAS	11.3	0.6302
P40261	Nicotinamide N-methyltransferase	NNMT	14.4	0.6340
P05120	Plasminogen activator inhibitor 2	SERPINB2	19	0.6361
P04216	Thy-1 membrane glycoprotein	THY1	14.9	0.6391
Q96G03	Phosphoglucomutase-2	PGM2	11.4	0.6403
P23229	Integrin alpha-6;Integrin alpha-6 heavy chain;Integrin alpha-6 light chain	ITGA6	3.3	0.6427
P32455;Q9H0R5	Interferon-induced guanylate-binding protein 1;Guanylate-binding protein 3	GBP1;GBP3	7.3	0.6508
P21926	CD9 antigen	CD9	11.4	0.6533
O94925	Glutaminase kidney isoform, mitochondrial	GLS	8.4	0.6576
P53396	ATP-citrate synthase	ACLY	22.3	0.6604
P15151	Poliovirus receptor	PVR	8.2	0.6649
O76021	Ribosomal L1 domain-containing protein 1	RSL1D1	8	0.6701
Q9Y2D5	A-kinase anchor protein 2	AKAP2	4.4	0.6713
P13612	Integrin alpha-4	ITGA4	2.6	0.6728

P48163	NADP-dependent malic enzyme	ME1	6.8	0.6759
Q16643	Drebrin	DBN1	10.2	0.6769
P37837	Transaldolase	TALDO1	32	0.6836
O76024	Wolframin	WFS1	4.6	0.6847
P09417	Dihydropteridine reductase	QDPR	24.2	0.6849
Q13907	Isopentenyl-diphosphate Delta-isomerase 1	IDI1	9.3	0.6862
Q02790	Peptidyl-prolyl cis-trans isomerase FKBP4;Peptidyl-prolyl cis-trans isomerase FKBP4, N-terminally processed	FKBP4	26.6	0.6884
Q6PIU2	Neutral cholesterol ester hydrolase 1	NCEH1	9.8	0.6895
P21291	Cysteine and glycine-rich protein 1	CSRP1	59.6	0.6926
P29317	Ephrin type-A receptor 2	EPHA2	7	0.6968
Q15274	Nicotinate-nucleotide pyrophosphorylase [carboxylating]	QPRT	6.7	0.6988
Q9UBI6	Guanine nucleotide-binding protein G(I)/G(S)/G(O) subunit gamma-12	GNG12	37.5	0.7079
P33991	DNA replication licensing factor MCM4	MCM4	7.2	0.7108
P07951	Tropomyosin beta chain	TPM2	41.2	0.7175
Q9Y696	Chloride intracellular channel protein 4	CLIC4	58.5	0.7179
P14324	Farnesyl pyrophosphate synthase	FDPS	6.9	0.7205
Q13557;Q13554;Q13555;Q9UQM7	Calcium/calmodulin-dependent protein kinase type II subunit delta	CAMK2D	15.8	0.7237
P12429	Annexin A3	ANXA3	6.2	0.7247
Q8N8S7	Protein enabled homolog	ENAH	9	0.7296
Q9Y281	Cofilin-2	CFL2	63.9	0.7301
O00410;O60518	Importin-5	IPO5	32	0.7309
Q16527	Cysteine and glycine-rich protein 2	CSRP2	10.4	0.7339
Q14847	LIM and SH3 domain protein 1	LASP1	21.8	0.7341
P35613	Basigin	BSG	10.4	0.7356
Q96CS3	FAS-associated factor 2	FAF2	7.9	0.7367
Q9BW60	Elongation of very long chain fatty acids protein 1	ELOVL1	9	0.7412
P46977	Dolichyl-diphosphooligosaccharide--protein glycosyltransferase subunit STT3A	STT3A	4.7	0.7467
Q02952	A-kinase anchor protein 12	AKAP12	11	0.7472

P60033	CD81 antigen	CD81	25	0.7540
Q9BUF5	Tubulin beta-6 chain	TUBB6	27.8	0.7540
P31153	S-adenosylmethionine synthase isoform type-2	MAT2A	8.1	0.7553
Q969G5	Protein kinase C delta-binding protein	PRKCDBP	10.3	0.7557
O00159	Unconventional myosin-Ic	MYO1C	6.1	0.7573
P21266	Glutathione S-transferase Mu 3	GSTM3	16.4	0.7582
P21281	V-type proton ATPase subunit B, brain isoform	ATP6V1B2	20.2	0.7602
P07355;A6NMY6	Annexin A2;Putative annexin A2-like protein	ANXA2;ANXA2P2	46.6	0.7651
Q16629	Serine/arginine-rich splicing factor 7	SRSF7	7.6	0.7656
O60684;P52294	Importin subunit alpha-7;Importin subunit alpha-1	KPNA6;KPNA1	6.5	0.7667
Q16881;Q9NNW7	Thioredoxin reductase 1, cytoplasmic	TXNRD1	23.6	0.7675
P62993	Growth factor receptor-bound protein 2	GRB2	8.8	0.7686
P04844	Dolichyl-diphosphooligosaccharide--protein glycosyltransferase subunit 2	RPN2	24.9	0.7695
Q9BWD1	Acetyl-CoA acetyltransferase, cytosolic	ACAT2	38.3	0.7695
Q9Y4F1	FERM, RhoGEF and pleckstrin domain-containing protein 1	FARP1	6.3	0.7708
Q6NZI2	Polymerase I and transcript release factor	PTRF	20.5	0.7709
P63261	Actin, cytoplasmic 2;Actin, cytoplasmic 2, N-terminally processed	ACTG1	50.4	0.7715
Q08623	Pseudouridine-5-monophosphatase	HDHD1	12.3	0.7732
P41227	N-alpha-acetyltransferase 10	NAA10	19.6	0.7735
P23634	Plasma membrane calcium-transporting ATPase 4	ATP2B4	12.4	0.7760
P13987	CD59 glycoprotein	CD59	18	0.7800
P30520	Adenylosuccinate synthetase isozyme 2	ADSS	7.7	0.7800
P50454	Serpin H1	SERPINH1	45.9	0.7803
O75083	WD repeat-containing protein 1	WDR1	52.5	0.7807
Q13637	Ras-related protein Rab-32	RAB32	12	0.7814
P52788	Spermine synthase	SMS	24.6	0.7817
P52292	Importin subunit alpha-2	KPNA2	13.6	0.7822

P46063	ATP-dependent DNA helicase Q1	RECQL	5.1	0.7828
Q92747	Actin-related protein 2/3 complex subunit 1A	ARPC1A	23	0.7849
P26038	Moesin	MSN	41.9	0.7859
P30740	Leukocyte elastase inhibitor	SERPINB1	6.3	0.7859
P35241	Radixin	RDX	19.4	0.7878
Q6DD88	Atlastin-3	ATL3	25.7	0.7881
P60983	Glia maturation factor beta	GMFB	12	0.7886
Q6UVK1	Chondroitin sulfate proteoglycan 4	CSPG4	1.6	0.7890
Q9UDY4	DnaJ homolog subfamily B member 4	DNAJB4	9.2	0.7907
P07099	Epoxide hydrolase 1	EPHX1	11.6	0.7921
P17844	Probable ATP-dependent RNA helicase DDX5	DDX5	13.8	0.7938
O75131	Copine-3	CPNE3	13.6	0.7941
Q9ULV4	Coronin-1C	CORO1C	36.3	0.7945
P60981	Destrin	DSTN	58.2	0.7961
P22307	Non-specific lipid-transfer protein	SCP2	13.5	0.7977
Q86Y82	Syntaxin-12	STX12	6.5	0.7978
O75534	Cold shock domain-containing protein E1	CSDE1	5.3	0.7981
P35221	Catenin alpha-1	CTNNA1	24.1	0.7985
P17252;P05771	Protein kinase C alpha type;Protein kinase C beta type	PRKCA;PRKCB	4.2	0.7987
P30041	Peroxiredoxin-6	PRDX6	33.5	0.7997
O95817	BAG family molecular chaperone regulator 3	BAG3	10.4	0.8002
Q96AG4	Leucine-rich repeat-containing protein 59	LRRC59	31.3	0.8007
P49327	Fatty acid synthase;[Acyl-carrier-protein] S-acetyltransferase;[Acyl-carrier-protein] S-malonyltransferase;3-oxoacyl-[acyl-carrier-protein] synthase;3-oxoacyl-[acyl-carrier-protein] reductase;3-hydroxypalmitoyl-[acyl-carrier-protein] dehydratase;Enoyl-[acyl-carrier-protein] reductase;Oleoyl-[acyl-carrier-protein] hydrolase	FASN	12.8	0.8073
P51911	Calponin-1	CNN1	59.9	0.8130
P09497	Clathrin light chain B	CLTB	7.4	0.8137
Q9Y639	Neuroplastin	NPTN	4.5	0.8137
Q07021	Complement component 1 Q subcomponent-binding protein, mitochondrial	C1QBP	16.3	0.8144
Q9UMS6	Synaptopodin-2	SYNPO2	8.8	0.8157

P13861	cAMP-dependent protein kinase type II-alpha regulatory subunit	PRKAR2A	4.2	0.8157
P80723	Brain acid soluble protein 1	BASP1	63.4	0.8157
O00425;Q9Y6M1;Q9NZI8	Insulin-like growth factor 2 mRNA-binding protein 3;Insulin-like growth factor 2 mRNA-binding protein 2;Insulin-like growth factor 2 mRNA-binding protein 1	IGF2BP3;IGF2BP2;IGF2BP1	4.7	0.8158
Q9UKY7	Protein CDV3 homolog	CDV3	22.1	0.8159
P26583	High mobility group protein B2	HMGB2	26.3	0.8184
Q9H3N1	Thioredoxin-related transmembrane protein 1	TMX1	15	0.8198
Q96AY3	Peptidyl-prolyl cis-trans isomerase FKBP10	FKBP10	13.2	0.8203
P46060	Ran GTPase-activating protein 1	RANGAP1	22.8	0.8233
Q9UI12	V-type proton ATPase subunit H	ATP6V1H	10.4	0.8256
Q53GQ0	Estradiol 17-beta-dehydrogenase 12	HSD17B12	24.7	0.8274
P84098	60S ribosomal protein L19	RPL19	9.7	0.8284
Q96JJ7	Protein disulfide-isomerase TMX3	TMX3	5.3	0.8297
P16615;Q93084;O14983	Sarcoplasmic/endoplasmic reticulum calcium ATPase 2	ATP2A2	16.1	0.8311
Q15404	Ras suppressor protein 1	RSU1	38.6	0.8311
P54577	Tyrosine--tRNA ligase, cytoplasmic	YARS	13.1	0.8316
Q9UNF0	Protein kinase C and casein kinase substrate in neurons protein 2	PACSIN2	7.6	0.8361
P13797;Q14651;P13796	Plastin-3	PLS3	45.6	0.8377
P13693;Q56UQ5	Translationally-controlled tumor protein	TPT1	51.2	0.8386
P09651;Q32P51	Heterogeneous nuclear ribonucleoprotein A1;Heterogeneous nuclear ribonucleoprotein A1-like 2	HNRNPA1;HNRNPA1L2	21.2	0.8389
Q6DKJ4	Nucleoredoxin	NXN	9.9	0.8389
P16070	CD44 antigen	CD44	8.8	0.8397
P61254;Q9UNX3	60S ribosomal protein L26;60S ribosomal protein L26-like 1	RPL26;RPL26L1	26.9	0.8400
P55010	Eukaryotic translation initiation factor 5	EIF5	7.9	0.8403

P05067	Amyloid beta A4 protein;N-APP;Soluble APP-alpha;Soluble APP-beta;C99;Beta-amyloid protein 42;Beta-amyloid protein 40;C83;P3(42);P3(40);C80;Gamma-secretase C-terminal fragment 59;Gamma-secretase C-terminal fragment 57;Gamma-secretase C-terminal fragment 50;C31	APP	3.8	0.8415
P46779	60S ribosomal protein L28	RPL28	27.7	0.8420
Q8IVL6	Prolyl 3-hydroxylase 3	LEPREL2	5.3	0.8429
P48643	T-complex protein 1 subunit epsilon	CCT5	25	0.8447
P15531	Nucleoside diphosphate kinase A	NME1	21.1	0.8449
Q07065	Cytoskeleton-associated protein 4	CKAP4	22.1	0.8452
P62736;P63267	Actin, aortic smooth muscle;Actin, gamma-enteric smooth muscle	ACTA2;ACTG2	56.8	0.8460
Q9BRX2	Protein pelota homolog	PELO	12.5	0.8472
P30085	UMP-CMP kinase	CMPK1	33.2	0.8476
Q15075	Early endosome antigen 1	EEA1	3.3	0.8481
P47813;O14602	Eukaryotic translation initiation factor 1A, X-chromosomal;Eukaryotic translation initiation factor 1A, Y-chromosomal	EIF1AX;EIF1AY	29.9	0.8482
Q05682	Caldesmon	CALD1	21.8	0.8482
P21964	Catechol O-methyltransferase	COMT	16.2	0.8487
P16152	Carbonyl reductase [NADPH] 1	CBR1	22	0.8495
P29401	Transketolase	TKT	36.6	0.8522
P54727;P54725	UV excision repair protein RAD23 homolog B	RAD23B	17.8	0.8522
O95816	BAG family molecular chaperone regulator 2	BAG2	39.8	0.8525
P05455	Lupus La protein	SSB	27.5	0.8531
Q9UHQ9	NADH-cytochrome b5 reductase 1	CYB5R1	12.1	0.8531
P13674	Prolyl 4-hydroxylase subunit alpha-1	P4HA1	19.5	0.8532
Q9UPN3	Microtubule-actin cross-linking factor 1, isoforms 1/2/3/5	MACF1	1.4	0.8535
P23588	Eukaryotic translation initiation factor 4B	EIF4B	3.4	0.8540
Q14914	Prostaglandin reductase 1	PTGR1	22.2	0.8546
O43242	26S proteasome non-ATPase regulatory subunit 3	PSMD3	6.9	0.8550
P54886	Delta-1-pyrroline-5-carboxylate synthase;Glutamate 5-kinase;Gamma-glutamyl phosphate reductase	ALDH18A1	4.9	0.8562
P58546	Myotrophin	MTPN	61	0.8569

Q15417	Calponin-3	CNN3	36.2	0.8595
O60701	UDP-glucose 6-dehydrogenase	UGDH	32	0.8596
Q8WXF1	Paraspeckle component 1	PSPC1	7.5	0.8602
O75369	Filamin-B	FLNB	35.6	0.8607
Q9NQC3	Reticulon-4	RTN4	3.7	0.8614
P51571	Translocon-associated protein subunit delta	SSR4	14.5	0.8615
Q01082;O15020;P11277;Q9H254	Spectrin beta chain, brain 1	SPTBN1	11.6	0.8618
Q70UQ0	Inhibitor of nuclear factor kappa-B kinase-interacting protein	IKBIP	20.3	0.8619
O43795;Q9UBC5	Unconventional myosin-Ib	MYO1B	8.2	0.8620
O94826	Mitochondrial import receptor subunit TOM70	TOMM70A	2.8	0.8624
P09960	Leukotriene A-4 hydrolase	LTA4H	15.5	0.8629
Q04760	Lactoylglutathione lyase	GLO1	31	0.8635
P04181	Ornithine aminotransferase, mitochondrial;Ornithine aminotransferase, hepatic form;Ornithine aminotransferase, renal form	OAT	16.2	0.8650
Q86UE4	Protein LYRIC	MTDH	14.4	0.8655
Q16836	Hydroxyacyl-coenzyme A dehydrogenase, mitochondrial	HADH	26.8	0.8663
P46108	Adapter molecule crk	CRK	14.1	0.8669
Q9H4M9; Q9H223	EH domain-containing protein 1	EHD1	19.5	0.8669
P49821	NADH dehydrogenase [ubiquinone] flavoprotein 1, mitochondrial	NDUFV1	15.5	0.8681
Q15029	116 kDa U5 small nuclear ribonucleoprotein component	EFTUD2	5.8	0.8681
Q96HY6	DDRGK domain-containing protein 1	DDRGK1	9.9	0.8692
Q9Y3A5	Ribosome maturation protein SBDS	SBDS	14.4	0.8702
O75718	Cartilage-associated protein	CRTAP	10	0.8704
Q13813	Spectrin alpha chain, brain	SPTAN1	10.3	0.8705
O95433	Activator of 90 kDa heat shock protein ATPase homolog 1	AHSA1	16.3	0.8706
Q0ZGT2	Nexilin	NEXN	8.1	0.8710

Q9UBQ5	Eukaryotic translation initiation factor 3 subunit K	EIF3K	25.7	0.8712
P61313	60S ribosomal protein L15	RPL15	13.7	0.8714
O15371	Eukaryotic translation initiation factor 3 subunit D	EIF3D	13.7	0.8715
P21333	Filamin-A	FLNA	36.7	0.8726
P11233;P11234	Ras-related protein Ral-A;Ras-related protein Ral-B	RALA;RALB	11.7	0.8731
P49207	60S ribosomal protein L34	RPL34	21.4	0.8738
Q9UQ80	Proliferation-associated protein 2G4	PA2G4	35	0.8751
Q9NX40	OCIA domain-containing protein 1	OCIAD1	12.7	0.8761
Q92783	Signal transducing adapter molecule 1	STAM	13	0.8768
Q9HB71	Calcyclin-binding protein	CACYBP	32	0.8784
Q15041	ADP-ribosylation factor-like protein 6-interacting protein 1	ARL6IP1	4.9	0.8792
Q06830	Peroxiredoxin-1	PRDX1	57.3	0.8796
Q9P0L0	Vesicle-associated membrane protein-associated protein A	VAPA	20.9	0.8802
Q02543	60S ribosomal protein L18a	RPL18A	26.7	0.8812
Q9NSD9	Phenylalanine--tRNA ligase beta subunit	FARSB	8	0.8818
Q01813	6-phosphofructokinase type C	PFKP	20.5	0.8824
P56211;O43768	cAMP-regulated phosphoprotein 19;Alpha-endosulfine	ARPP19;ENSA	22.3	0.8828
P06756	Integrin alpha-V;Integrin alpha-V heavy chain;Integrin alpha-V light chain	ITGAV	11.3	0.8834
O75436	Vacuolar protein sorting-associated protein 26A	VPS26A	9.5	0.8853
O60841	Eukaryotic translation initiation factor 5B	EIF5B	2	0.8859
Q15181	Inorganic pyrophosphatase	PPA1	47.1	0.8861
P04075;P09972	Fructose-bisphosphate aldolase A	ALDOA	51.1	0.8864
Q9BTT0	Acidic leucine-rich nuclear phosphoprotein 32 family member E	ANP32E	18.3	0.8865
Q9BT78	COP9 signalosome complex subunit 4	COPS4	13.3	0.8866
Q01105	Protein SET	SET	12.8	0.8869
P22234	Multifunctional protein ADE2;Phosphoribosylaminoimidazole-succinocarboxamide synthase;Phosphoribosylaminoimidazole carboxylase	PAICS	15.5	0.8872
Q7L576;Q	Cytoplasmic FMR1-interacting protein 1;Cytoplasmic	CYFIP1;C	2.1	0.8881

96F07	FMR1-interacting protein 2	YFIP2		
P08133	Annexin A6	ANXA6	31.1	0.8893
Q08257	Quinone oxidoreductase	CRYZ	11.9	0.8898
P62714;P67775	Serine/threonine-protein phosphatase 2A catalytic subunit beta isoform;Serine/threonine-protein phosphatase 2A catalytic subunit alpha isoform	PPP2CB;PPP2CA	9.4	0.8899
P15144	Aminopeptidase N	ANPEP	23.8	0.8906
P04083	Annexin A1	ANXA1	54.9	0.8913
O75347	Tubulin-specific chaperone A	TBCA	18.5	0.8917
P09486	SPARC	SPARC	25.1	0.8918
P38606	V-type proton ATPase catalytic subunit A	ATP6V1A	18.3	0.8922
Q96AC1	Fermitin family homolog 2	FERMT2	20	0.8954
P48637	Glutathione synthetase	GSS	8.9	0.8969
P19623	Spermidine synthase	SRM	7	0.8973
Q16543	Hsp90 co-chaperone Cdc37	CDC37	21.2	0.8979
P49588	Alanine--tRNA ligase, cytoplasmic	AARS	19.6	0.8983
O95373;O15397	Importin-7	IPO7	13.8	0.8986
Q9Y3F4	Serine-threonine kinase receptor-associated protein	STRAP	30.6	0.8994
P12081	Histidine--tRNA ligase, cytoplasmic	HARS	11	0.9003
P46459	Vesicle-fusing ATPase	NSF	5.5	0.9003
P63167;Q96FJ2	Dynein light chain 1, cytoplasmic;Dynein light chain 2, cytoplasmic	DYNLL1;DYNLL2	37.1	0.9010
P63241;Q9GZV4;Q6IS14	Eukaryotic translation initiation factor 5A-1;Eukaryotic translation initiation factor 5A-2;Eukaryotic translation initiation factor 5A-1-like	EIF5A;EIF5A2;EIF5AL1	29.2	0.9011
P41567;O60739	Eukaryotic translation initiation factor 1;Eukaryotic translation initiation factor 1b	EIF1;EIF1B	23.9	0.9022
Q92688	Acidic leucine-rich nuclear phosphoprotein 32 family member B	ANP32B	7.6	0.9022
P46976	Glycogenin-1	GYG1	7.7	0.9023
P07737;CON__P02584	Profilin-1	PFN1	81.4	0.9032
Q562R1	Beta-actin-like protein 2	ACTBL2	14.1	0.9038
P08572	Collagen alpha-2(IV) chain;Canstatin	COL4A2	1.5	0.9049

P83731	60S ribosomal protein L24	RPL24	21	0.9050
P11586	C-1-tetrahydrofolate synthase, cytoplasmic;Methylenetetrahydrofolate dehydrogenase;Methenyltetrahydrofolate cyclohydrolase;Formyltetrahydrofolate synthetase	MTHFD1	24.8	0.9056
P63151;Q66LE6;Q00005	Serine/threonine-protein phosphatase 2A 55 kDa regulatory subunit B alpha isoform	PPP2R2A	15	0.9064
Q9NVA2; Q14141;Q92599	Septin-11	Sep-11	23.1	0.9067
P68104;Q5VTE0;Q05639	Elongation factor 1-alpha 1;Putative elongation factor 1-alpha-like 3;Elongation factor 1-alpha 2	EEF1A1; EEF1A1P5;EEF1A2	52.6	0.9073
Q9C0H2	Protein tweety homolog 3	TTYH3	2.7	0.9075
P42677	40S ribosomal protein S27	RPS27	29.8	0.9076
Q9H4A6;Q9H4A5	Golgi phosphoprotein 3;Golgi phosphoprotein 3-like	GOLPH3; GOLPH3L	9.7	0.9077
P52566	Rho GDP-dissociation inhibitor 2	ARHGDIB	20.9	0.9081
Q02809	Procollagen-lysine,2-oxoglutarate 5-dioxygenase 1	PLOD1	10	0.9083
O60565	Gremlin-1	GREM1	20.1	0.9086
Q7L1Q6;Q9Y6E2	Basic leucine zipper and W2 domain-containing protein 1	BZW1	5.5	0.9103
O15511;Q9BPX5	Actin-related protein 2/3 complex subunit 5	ARPC5	26.5	0.9109
P35637;Q92804	RNA-binding protein FUS;TATA-binding protein-associated factor 2N	FUS;TAF15	4.8	0.9114
P78527	DNA-dependent protein kinase catalytic subunit	PRKDC	3.5	0.9117
P50552	Vasodilator-stimulated phosphoprotein	VASP	12.4	0.9121
O15145	Actin-related protein 2/3 complex subunit 3	ARPC3	39.3	0.9122
P61981	14-3-3 protein gamma;14-3-3 protein gamma, N-terminally processed	YWHAG	55.9	0.9126
P30153	Serine/threonine-protein phosphatase 2A 65 kDa regulatory subunit A alpha isoform	PPP2R1A	18.8	0.9134
O75533	Splicing factor 3B subunit 1	SF3B1	8.4	0.9142
P50990	T-complex protein 1 subunit theta	CCT8	32.1	0.9145

Q99873;Q9NR22	Protein arginine N-methyltransferase 1	PRMT1	22.7	0.9153
P19338	Nucleolin	NCL	23.9	0.9154
O95881	Thioredoxin domain-containing protein 12	TXNDC12	22.1	0.9164
Q03135	Caveolin-1	CAV1	21.3	0.9178
P22392;O60361	Nucleoside diphosphate kinase B;Putative nucleoside diphosphate kinase	NME2;NME2P1	40.1	0.9179
P53992	Protein transport protein Sec24C	SEC24C	3.2	0.9184
O94905;O75477	Erlin-2	ERLIN2	21.5	0.9190
O00154	Cytosolic acyl coenzyme A thioester hydrolase	ACOT7	11.3	0.9194
Q02818	Nucleobindin-1	NUCB1	4.6	0.9200
Q9UNZ2	NSFL1 cofactor p47	NSFL1C	14.6	0.9207
P41252	Isoleucine--tRNA ligase, cytoplasmic	IARS	13.7	0.9208
P83111	Serine beta-lactamase-like protein LACTB, mitochondrial	LACTB	6.6	0.9211
O43707	Alpha-actinin-4	ACTN4	40.6	0.9216
Q9P000	COMM domain-containing protein 9	COMMD9	18.7	0.9233
P62258	14-3-3 protein epsilon	YWHAE	45.5	0.9235
Q9Y617	Phosphoserine aminotransferase	PSAT1	22.2	0.9244
Q01518	Adenylyl cyclase-associated protein 1	CAP1	49.7	0.9246
P07108	Acyl-CoA-binding protein	DBI	29.9	0.9249
P40222	Alpha-taxilin	TXLNA	5.1	0.9249
O15372	Eukaryotic translation initiation factor 3 subunit H	EIF3H	30.1	0.9262
P05023;P50993;P13637;P54707;Q13733	Sodium/potassium-transporting ATPase subunit alpha-1	ATP1A1	11.2	0.9271
P63104	14-3-3 protein zeta/delta	YWHAZ	47.3	0.9271
Q7KZF4	Staphylococcal nuclease domain-containing protein 1	SND1	17.8	0.9282
P54920	Alpha-soluble NSF attachment protein	NAPA	15.9	0.9284
P00492	Hypoxanthine-guanine phosphoribosyltransferase	HPRT1	23.9	0.9286
P04843	Dolichyl-diphosphooligosaccharide--protein glycosyltransferase subunit 1	RPN1	22.1	0.9286
Q99832	T-complex protein 1 subunit eta	CCT7	37	0.9289

Q92973	Transportin-1	TNPO1	8.1	0.9294
P04080	Cystatin-B	CSTB	23.5	0.9296
Q09666	Neuroblast differentiation-associated protein AHNAK	AHNAK	47.9	0.9307
P08758	Annexin A5	ANXA5	24.7	0.9317
Q9UBQ7	Glyoxylate reductase/hydroxypyruvate reductase	GRHPR	15.2	0.9341
P09110	3-ketoacyl-CoA thiolase, peroxisomal	ACAA1	4	0.9343
P13639	Elongation factor 2	EEF2	36.2	0.9345
P60228	Eukaryotic translation initiation factor 3 subunit E	EIF3E	11.5	0.9355
P23528	Cofilin-1	CFL1	66.3	0.9358
P31946	14-3-3 protein beta/alpha;14-3-3 protein beta/alpha, N-terminally processed	YWHAB	42.3	0.9363
P07954	Fumarate hydratase, mitochondrial	FH	27.1	0.9368
P25398	40S ribosomal protein S12	RPS12	48.5	0.9369
P23526	Adenosylhomocysteinase	AHCY	15.5	0.9370
Q13347	Eukaryotic translation initiation factor 3 subunit I	EIF3I	28	0.9371
P24752	Acetyl-CoA acetyltransferase, mitochondrial	ACAT1	22.7	0.9381
Q13885;Q9BVA1;A6NNZ2	Tubulin beta-2A chain;Tubulin beta-2B chain	TUBB2A;TUBB2B	34.2	0.9392
O00186	Syntaxin-binding protein 3	STXBP3	2.9	0.9397
P06748	Nucleophosmin	NPM1	24.8	0.9399
Q16181;Q6ZU15	Septin-7	Sep-07	24.7	0.9402
P36578	60S ribosomal protein L4	RPL4	19.4	0.9403
Q14019	Coactosin-like protein	COTL1	33.1	0.9403
P68402	Platelet-activating factor acetylhydrolase IB subunit beta	PAFAH1B2	7.4	0.9404
Q9Y4E8	Ubiquitin carboxyl-terminal hydrolase 15	USP15	3.5	0.9407
P05556	Integrin beta-1	ITGB1	21.7	0.9420
Q9UJU6	Drebrin-like protein	DBNL	17.7	0.9423
Q13838	Spliceosome RNA helicase DDX39B	DDX39B	32	0.9424
Q9BSJ8	Extended synaptotagmin-1	ESYT1	11.8	0.9424
O43615	Mitochondrial import inner membrane translocase subunit TIM44	TIMM44	5.8	0.9427
Q04917	14-3-3 protein eta	YWHAH	34.1	0.9434
Q5VYK3	Proteasome-associated protein ECM29 homolog	ECM29	3.4	0.9439
P51572	B-cell receptor-associated protein 31	BCAP31	17.5	0.9444

P23921	Ribonucleoside-diphosphate reductase large subunit	RRM1	6.8	0.9449
P49591	Serine--tRNA ligase, cytoplasmic	SARS	19.1	0.9459
P17980	26S protease regulatory subunit 6A	PSMC3	21.4	0.9460
P68366	Tubulin alpha-4A chain	TUBA4A	25.4	0.9461
P35222;P14923	Catenin beta-1	CTNNB1	6.4	0.9469
P47755	F-actin-capping protein subunit alpha-2	CAPZA2	35	0.9471
Q13492;O60641	Phosphatidylinositol-binding clathrin assembly protein	PICALM	10	0.9471
P12814;P35609;Q08043	Alpha-actinin-1	ACTN1	37.6	0.9474
Q14974	Importin subunit beta-1	KPNB1	19.4	0.9474
P02786	Transferrin receptor protein 1;Transferrin receptor protein 1, serum form	TFRC	17.2	0.9483
Q07960	Rho GTPase-activating protein 1	ARHGAP1	10.7	0.9484
P02452;CON__Q862S4	Collagen alpha-1(I) chain	COL1A1	9.2	0.9485
P40227;Q92526	T-complex protein 1 subunit zeta	CCT6A	40.7	0.9492
P07814	Bifunctional glutamate/proline--tRNA ligase;Glutamate--tRNA ligase;Proline--tRNA ligase	EPRS	13	0.9502
P28838	Cytosol aminopeptidase	LAP3	12.9	0.9507
P12004	Proliferating cell nuclear antigen	PCNA	40.6	0.9511
P60900	Proteasome subunit alpha type-6	PSMA6	27.6	0.9511
P62495	Eukaryotic peptide chain release factor subunit 1	ETF1	14.9	0.9515
O95202	LETM1 and EF-hand domain-containing protein 1, mitochondrial	LETM1	4.9	0.9520
P62899	60S ribosomal protein L31	RPL31	30.4	0.9526
P49411	Elongation factor Tu, mitochondrial	TUFM	20.8	0.9527
Q15293	Reticulocalbin-1	RCN1	19.9	0.9535
Q16539	Mitogen-activated protein kinase 14	MAPK14	12.2	0.9535
P27105	Erythrocyte band 7 integral membrane protein	STOM	23.3	0.9537
Q13151	Heterogeneous nuclear ribonucleoprotein A0	HNRNPA0	8.9	0.9543
P18621	60S ribosomal protein L17	RPL17	32.1	0.9547

P35232	Prohibitin	PHB	22.4	0.9548
Q9Y4K0	Lysyl oxidase homolog 2	LOXL2	11.4	0.9564
P17858	6-phosphofructokinase, liver type	PFKL	9.9	0.9565
Q9UHX1	Poly(U)-binding-splicing factor PUF60	PUF60	11.6	0.9565
Q8NC51	Plasminogen activator inhibitor 1 RNA-binding protein	SERBP1	9.1	0.9566
O75822	Eukaryotic translation initiation factor 3 subunit J	EIF3J	9.3	0.9569
P62910	60S ribosomal protein L32	RPL32	32.6	0.9571
Q92734	Protein TFG	TFG	6.8	0.9572
P49720	Proteasome subunit beta type-3	PSMB3	14.1	0.9579
O60506	Heterogeneous nuclear ribonucleoprotein Q	SYNCRIP	12.2	0.9591
P41250	Glycine--tRNA ligase	GARS	17.6	0.9595
Q14152	Eukaryotic translation initiation factor 3 subunit A	EIF3A	11.3	0.9595
P55786;A6NEC2	Puromycin-sensitive aminopeptidase	NPEPPS	16.6	0.9596
Q14696	LDLR chaperone MESD	MESDC2	9	0.9596
P11142	Heat shock cognate 71 kDa protein	HSPA8	37.5	0.9597
Q13765	Nascent polypeptide-associated complex subunit alpha	NACA	20.5	0.9597
Q7Z4H8	KDEL motif-containing protein 2	KDELC2	4.7	0.9597
P29966	Myristoylated alanine-rich C-kinase substrate	MARCKS	34.6	0.9599
O43852	Calumenin	CALU	45.7	0.9602
Q15121	Astrocytic phosphoprotein PEA-15	PEA15	16.9	0.9602
O00231	26S proteasome non-ATPase regulatory subunit 11	PSMD11	17.8	0.9602
P39687;O43423;O95626	Acidic leucine-rich nuclear phosphoprotein 32 family member A;Acidic leucine-rich nuclear phosphoprotein 32 family member C;Acidic leucine-rich nuclear phosphoprotein 32 family member D	ANP32A; ANP32C; ANP32D	12	0.9605
Q9NR12	PDZ and LIM domain protein 7	PDLIM7	27.6	0.9605
P25787	Proteasome subunit alpha type-2	PSMA2	23.9	0.9609
P56537	Eukaryotic translation initiation factor 6	EIF6	15.5	0.9612
P00568	Adenylate kinase isoenzyme 1	AK1	17.5	0.9613
Q15691	Microtubule-associated protein RP/EB family member 1	MAPRE1	38.8	0.9613
O00629	Importin subunit alpha-4	KPNA4	17.3	0.9614
P55072	Transitional endoplasmic reticulum ATPase	VCP	32.1	0.9614
P61160	Actin-related protein 2	ACTR2	26.4	0.9614
P63000;P60763	Ras-related C3 botulinum toxin substrate 1;Ras-related C3 botulinum toxin substrate 3	RAC1;RAC3	25.5	0.9623

Q16658	Fascin	FSCN1	19.5	0.9623
P26639	Threonine--tRNA ligase, cytoplasmic	TARS	9.7	0.9630
O95782	AP-2 complex subunit alpha-1	AP2A1	12.7	0.9634
P62913	60S ribosomal protein L11	RPL11	9	0.9640
Q15746	Myosin light chain kinase, smooth muscle;Myosin light chain kinase, smooth muscle, deglutamylated form	MYLK	10.8	0.9648
P17987	T-complex protein 1 subunit alpha	TCP1	33.8	0.9653
Q9UJ70	N-acetyl-D-glucosamine kinase	NAGK	14.2	0.9655
Q86UP2	Kinectin	KTN1	6.3	0.9657
O15143	Actin-related protein 2/3 complex subunit 1B	ARPC1B	36	0.9659
P68371	Tubulin beta-4B chain	TUBB4B	27.9	0.9670
Q5T4S7	E3 ubiquitin-protein ligase UBR4	UBR4	2.5	0.9671
P06733;P13929	Alpha-enolase	ENO1	43.3	0.9673
P62241	40S ribosomal protein S8	RPS8	20.7	0.9675
Q15185	Prostaglandin E synthase 3	PTGES3	31.2	0.9680
P78371	T-complex protein 1 subunit beta	CCT2	29.3	0.9689
Q8N766	Uncharacterized protein KIAA0090	KIAA0090	5.1	0.9689
Q02878	60S ribosomal protein L6	RPL6	21.5	0.9692
P06730;A6NMX2	Eukaryotic translation initiation factor 4E	EIF4E	11.5	0.9701
P37802	Transgelin-2	TAGLN2	59.3	0.9703
P61247	40S ribosomal protein S3a	RPS3A	29.9	0.9706
P62877	E3 ubiquitin-protein ligase RBX1	RBX1	17.6	0.9710
P67870	Casein kinase II subunit beta	CSNK2B	18.1	0.9710
Q16576	Histone-binding protein RBBP7	RBBP7	18.8	0.9714
P61158;Q9P1U1;Q9C0K3	Actin-related protein 3	ACTR3	42.8	0.9720
P62424	60S ribosomal protein L7a	RPL7A	38.3	0.9727
P00338	L-lactate dehydrogenase A chain	LDHA	59.9	0.9737
P62753	40S ribosomal protein S6	RPS6	25.7	0.9738
O43396	Thioredoxin-like protein 1	TXNL1	20.4	0.9745
P11940;Q9H361;Q4VXU2;Q96D	Polyadenylate-binding protein 1	PABPC1	13.1	0.9745

U9				
P10620	Microsomal glutathione S-transferase 1	MGST1	19.4	0.9748
Q9Y490	Talin-1	TLN1	27	0.9755
Q96T76	MMS19 nucleotide excision repair protein homolog	MMS19	3.4	0.9767
Q9NZM1; O75923	Myoferlin	MYOF	17	0.9768
P62826	GTP-binding nuclear protein Ran	RAN	29.2	0.9773
P46781	40S ribosomal protein S9	RPS9	13.4	0.9774
Q15942	Zyxin	ZYX	13.8	0.9775
Q4L180	Filamin A-interacting protein 1-like	FILIP1L	2.6	0.9777
P30038	Delta-1-pyrroline-5-carboxylate dehydrogenase, mitochondrial	ALDH4A1	3.4	0.9784
Q9BQE3	Tubulin alpha-1C chain	TUBA1C	26.3	0.9788
Q15233	Non-POU domain-containing octamer-binding protein	NONO	11.3	0.9791
P12109	Collagen alpha-1(VI) chain	COL6A1	4.3	0.9800
O15144	Actin-related protein 2/3 complex subunit 2	ARPC2	35.3	0.9802
O43504	Hepatitis B virus X-interacting protein	HBXIP	26.4	0.9813
P22314	Ubiquitin-like modifier-activating enzyme 1	UBA1	28.8	0.9814
Q99471	Prefoldin subunit 5	PFDN5	23.4	0.9814
P62701;P22090;Q8TD47	40S ribosomal protein S4, X isoform;40S ribosomal protein S4, Y isoform 1	RPS4X;RPS4Y1	16	0.9818
P60903	Protein S100-A10	S100A10	24.7	0.9820
P49915	GMP synthase [glutamine-hydrolyzing]	GMPS	8.9	0.9823
Q9Y5S9	RNA-binding protein 8A	RBM8A	21.3	0.9827
P35237	Serpin B6	SERPINB6	40.2	0.9836
P08865	40S ribosomal protein SA	RPSA	26.4	0.9840
P08708;P0CW22	40S ribosomal protein S17;40S ribosomal protein S17-like	RPS17;RPS17L	39.3	0.9848
O43175	D-3-phosphoglycerate dehydrogenase	PHGDH	23.3	0.9849
Q96AE4	Far upstream element-binding protein 1	FUBP1	10.4	0.9849
Q15019	Septin-2	Sep-02	16.3	0.9854
Q5GLZ8	Probable E3 ubiquitin-protein ligase HERC4	HERC4	6.5	0.9855
P46778	60S ribosomal protein L21	RPL21	25	0.9856
P45973	Chromobox protein homolog 5	CBX5	18.8	0.9862
P09525	Annexin A4	ANXA4	16	0.9867

Q9NW15	Anoctamin-10	ANO10	9.4	0.9874
Q9Y6E0;O00506;Q9P289	Serine/threonine-protein kinase 24;Serine/threonine-protein kinase 24 36 kDa subunit;Serine/threonine-protein kinase 24 12 kDa subunit;Serine/threonine-protein kinase 25;Serine/threonine-protein kinase MST4	STK24;STK25;MST4	5	0.9877
P10768	S-formylglutathione hydrolase	ESD	17.7	0.9878
Q9ULC4	Malignant T-cell-amplified sequence 1	MCTS1	40.9	0.9884
P46777	60S ribosomal protein L5	RPL5	24.6	0.9889
P17812;Q9NRF8	CTP synthase 1	CTPS	14	0.9890
P62191	26S protease regulatory subunit 4	PSMC1	20.7	0.9892
P63244	Guanine nucleotide-binding protein subunit beta-2-like 1	GNB2L1	46.1	0.9895
P30084	Enoyl-CoA hydratase, mitochondrial	ECHS1	25.5	0.9906
P39023;Q92901	60S ribosomal protein L3	RPL3	32	0.9908
P60842;P38919	Eukaryotic initiation factor 4A-I	EIF4A1	25.9	0.9909
Q9Y5B9	FACT complex subunit SPT16	SUPT16H	4.1	0.9911
O14964	Hepatocyte growth factor-regulated tyrosine kinase substrate	HGS	3.9	0.9921
Q9NP72	Ras-related protein Rab-18	RAB18	32	0.9923
P62269	40S ribosomal protein S18	RPS18	27.6	0.9927
P09936	Ubiquitin carboxyl-terminal hydrolase isozyme L1	UCHL1	32.3	0.9933
Q92616	Translational activator GCN1	GCN1L1	4.6	0.9937
Q99613	Eukaryotic translation initiation factor 3 subunit C	EIF3C	3.9	0.9942
O00244	Copper transport protein ATOX1	ATOX1	38.2	0.9943
P24534	Elongation factor 1-beta	EEF1B2	20	0.9949
Q12797	Aspartyl/asparaginyl beta-hydroxylase	ASPH	8.3	0.9953
Q9H4A4	Aminopeptidase B	RNPEP	12.2	0.9955
Q12765	Secernin-1	SCRN1	12.1	0.9958
P30050	60S ribosomal protein L12	RPL12	28.5	0.9975
P00387	NADH-cytochrome b5 reductase 3;NADH-cytochrome b5 reductase 3 membrane-bound form;NADH-cytochrome b5 reductase 3 soluble form	CYB5R3	18.3	0.9979
O75821	Eukaryotic translation initiation factor 3 subunit G	EIF3G	15.6	0.9981

P20340;Q9NRW1	Ras-related protein Rab-6A;Ras-related protein Rab-6B	RAB6A;RAB6B	11.5	0.9981
O43143	Putative pre-mRNA-splicing factor ATP-dependent RNA helicase DHX15	DHX15	3.4	0.9984
P52907	F-actin-capping protein subunit alpha-1	CAPZA1	38.8	0.9985
P35754	Glutaredoxin-1	GLRX	17.9	0.9988
P25445	Tumor necrosis factor receptor superfamily member 6	FAS	12.8	0.9994
P63220	40S ribosomal protein S21	RPS21	30.1	0.9996
O00469	Procollagen-lysine,2-oxoglutarate 5-dioxygenase 2	PLOD2	9.4	0.9998
P14618;P30613	Pyruvate kinase isozymes M1/M2	PKM2	44.1	1.0002
Q32MZ4	Leucine-rich repeat flightless-interacting protein 1	LRRFIP1	5.1	1.0004
Q6PKG0	La-related protein 1	LARP1	3.3	1.0005
Q9NR45	Sialic acid synthase	NANS	18.1	1.0005
Q9NQR4	Omega-amidase NIT2	NIT2	11.6	1.0005
O94979	Protein transport protein Sec31A	SEC31A	13.9	1.0006
Q13200	26S proteasome non-ATPase regulatory subunit 2	PSMD2	21.9	1.0007
P60953;P17081;Q9H4E5;P15153;P84095	Cell division control protein 42 homolog	CDC42	37.2	1.0010
P22061	Protein-L-isoaspartate(D-aspartate) O-methyltransferase	PCMT1	15	1.0014
P62851	40S ribosomal protein S25	RPS25	14.4	1.0020
Q9BQA1	Methylosome protein 50	WDR77	9.9	1.0021
P62888	60S ribosomal protein L30	RPL30	26.1	1.0024
P50991	T-complex protein 1 subunit delta	CCT4	36.5	1.0025
Q16630	Cleavage and polyadenylation specificity factor subunit 6	CPSF6	6.2	1.0025
P21589	5-nucleotidase	NT5E	27.9	1.0027
P42167;P42166	Lamina-associated polypeptide 2, isoforms beta/gamma;Thymopoietin;Thymopentin;Lamina-associated polypeptide 2, isoform alpha;Thymopoietin;Thymopentin	TMPO	6.2	1.0029
P14866;Q8WVV9	Heterogeneous nuclear ribonucleoprotein L	HNRNPL	33.4	1.0033
P62633	Cellular nucleic acid-binding protein	CNBP	14.1	1.0035
P22695	Cytochrome b-c1 complex subunit 2, mitochondrial	UQCRC2	10.4	1.0035

Q13162	Peroxiredoxin-4	PRDX4	31.4	1.0037
Q9UJZ1	Stomatin-like protein 2	STOML2	12.9	1.0037
P55735	Protein SEC13 homolog	SEC13	23.3	1.0037
P43686	26S protease regulatory subunit 6B	PSMC4	25.4	1.0042
P10809	60 kDa heat shock protein, mitochondrial	HSPD1	43.8	1.0043
P34932	Heat shock 70 kDa protein 4	HSPA4	28.2	1.0045
O14980	Exportin-1	XPO1	6.3	1.0049
P62158;P27482	Calmodulin	CALM1	20.1	1.0049
Q15149;P58107	Plectin	PLEC	14.8	1.0050
Q99460	26S proteasome non-ATPase regulatory subunit 1	PSMD1	22.2	1.0052
P26368	Splicing factor U2AF 65 kDa subunit	U2AF2	9.3	1.0059
Q9P2E9	Ribosome-binding protein 1	RRBP1	14.6	1.0066
P52597	Heterogeneous nuclear ribonucleoprotein F;Heterogeneous nuclear ribonucleoprotein F, N-terminally processed	HNRNPF	11.1	1.0069
P59998	Actin-related protein 2/3 complex subunit 4	ARPC4	18.5	1.0070
Q15819	Ubiquitin-conjugating enzyme E2 variant 2	UBE2V2	20.7	1.0070
P09496	Clathrin light chain A	CLTA	9.7	1.0080
P52306	Rap1 GTPase-GDP dissociation stimulator 1	RAP1GDS1	10.5	1.0084
P62906	60S ribosomal protein L10a	RPL10A	34.6	1.0087
O15460	Prolyl 4-hydroxylase subunit alpha-2	P4HA2	25	1.0089
P08107	Heat shock 70 kDa protein 1A/1B	HSPA1A	25.9	1.0091
Q9Y2V2	Calcium-regulated heat stable protein 1	CARHSP1	42.9	1.0092
P10599	Thioredoxin	TXN	63.8	1.0097
P06744;CON__Q3ZBD7	Glucose-6-phosphate isomerase	GPI	22.4	1.0099
O60888	Protein CutA	CUTA	23.5	1.0116
Q9P0K7	Ankycorbin	RAI14	4.5	1.0122
Q92945	Far upstream element-binding protein 2	KHSRP	8	1.0122
Q96CV9	Optineurin	OPTN	13.3	1.0130
Q9NYL9	Tropomodulin-3	TMOD3	21.9	1.0134
P49368	T-complex protein 1 subunit gamma	CCT3	25.3	1.0135

P28482	Mitogen-activated protein kinase 1	MAPK1	10.3	1.0149
P55209	Nucleosome assembly protein 1-like 1	NAP1L1	13	1.0153
P51991	Heterogeneous nuclear ribonucleoprotein A3	HNRNPA3	14.3	1.0153
O00303	Eukaryotic translation initiation factor 3 subunit F	EIF3F	21.6	1.0155
P62917	60S ribosomal protein L8	RPL8	28.4	1.0159
Q92499	ATP-dependent RNA helicase DDX1	DDX1	18.8	1.0169
Q13418	Integrin-linked protein kinase	ILK	7.5	1.0170
P27816	Microtubule-associated protein 4	MAP4	28.2	1.0181
Q99439	Calponin-2	CNN2	28.5	1.0181
O14818;Q8TAA3	Proteasome subunit alpha type-7;Proteasome subunit alpha type-7-like	PSMA7;PSMA8	16.1	1.0182
Q00341	Vigilin	HDLBP	14	1.0190
P04406	Glyceraldehyde-3-phosphate dehydrogenase	GAPDH	51.3	1.0194
P62841	40S ribosomal protein S15	RPS15	21.4	1.0207
P61970	Nuclear transport factor 2	NUTF2	45.7	1.0207
Q99829	Copine-1	CPNE1	11.7	1.0211
P54578	Ubiquitin carboxyl-terminal hydrolase 14	USP14	8.3	1.0215
Q9HDC9	Adipocyte plasma membrane-associated protein	APMAP	9.6	1.0217
Q9NTK5	Obg-like ATPase 1	OLA1	13.6	1.0217
Q99497	Protein DJ-1	PARK7	48.1	1.0220
P51148	Ras-related protein Rab-5C	RAB5C	18.1	1.0222
P48739	Phosphatidylinositol transfer protein beta isoform	PITPNB	13.3	1.0222
P04350	Tubulin beta-4A chain	TUBB4A	18.2	1.0230
P62136;P62140;P36873	Serine/threonine-protein phosphatase PP1-alpha catalytic subunit;Serine/threonine-protein phosphatase PP1-beta catalytic subunit;Serine/threonine-protein phosphatase PP1-gamma catalytic subunit	PPP1CA; PPP1CB; PPP1CC	18.2	1.0230
P12955	Xaa-Pro dipeptidase	PEPD	4.1	1.0230
P62847	40S ribosomal protein S24	RPS24	19.5	1.0239
P43487	Ran-specific GTPase-activating protein	RANBP1	10.9	1.0251
P48681	Nestin	NES	6.2	1.0256
P62750	60S ribosomal protein L23a	RPL23A	35.9	1.0257
Q99584	Protein S100-A13	S100A13	32.7	1.0257
P54652	Heat shock-related 70 kDa protein 2	HSPA2	15.6	1.0260
Q13185;P83916	Chromobox protein homolog 3	CBX3	23	1.0262

P10515	Dihydrolipoyllysine-residue acetyltransferase component of pyruvate dehydrogenase complex, mitochondrial	DLAT	8.2	1.0266
P42704	Leucine-rich PPR motif-containing protein, mitochondrial	LRPPRC	10.3	1.0273
Q14103	Heterogeneous nuclear ribonucleoprotein D0	HNRNPD	14.6	1.0275
P62829	60S ribosomal protein L23	RPL23	38.6	1.0279
Q9UNM6	26S proteasome non-ATPase regulatory subunit 13	PSMD13	16.2	1.0288
Q32P28	Prolyl 3-hydroxylase 1	LEPRE1	4.9	1.0294
P02545	Prelamin-A/C;Lamin-A/C	LMNA	21.5	1.0294
P27348	14-3-3 protein theta	YWHAQ	28.6	1.0295
P84090	Enhancer of rudimentary homolog	ERH	37.5	1.0300
P50395	Rab GDP dissociation inhibitor beta	GDI2	40.7	1.0302
O14979	Heterogeneous nuclear ribonucleoprotein D-like	HNRPDL	16.7	1.0306
O75643	U5 small nuclear ribonucleoprotein 200 kDa helicase	SNRNP200	2.2	1.0309
P43243	Matrin-3	MATR3	8.3	1.0309
Q9P1F3	Costars family protein ABRACL	ABRACL	16	1.0313
P67936	Tropomyosin alpha-4 chain	TPM4	43.5	1.0339
P47756	F-actin-capping protein subunit beta	CAPZB	16.2	1.0339
P31939	Bifunctional purine biosynthesis protein PURH;Phosphoribosylaminoimidazolecarboxamide formyltransferase;IMP cyclohydrolase	ATIC	26	1.0340
P61513;A6NKH3	60S ribosomal protein L37a	RPL37A	41.3	1.0342
P62195	26S protease regulatory subunit 8	PSMC5	21.2	1.0346
P54136	Arginine--tRNA ligase, cytoplasmic	RARS	14.7	1.0354
Q5JRX3	Presequence protease, mitochondrial	PITRM1	6	1.0355
Q96QD8	Sodium-coupled neutral amino acid transporter 2	SLC38A2	7.3	1.0357
Q86W92	Liprin-beta-1	PPFIBP1	2.9	1.0358
Q9Y266	Nuclear migration protein nudC	NUDC	26.9	1.0362
P22102	Trifunctional purine biosynthetic protein adenosine-3;Phosphoribosylamine--glycine ligase;Phosphoribosylformylglycinamidine cyclo-ligase;Phosphoribosylglycinamide formyltransferase	GART	12.7	1.0364
P26641	Elongation factor 1-gamma	EEF1G	27.9	1.0367

P02461;CON__P04258	Collagen alpha-1(III) chain	COL3A1	2.6	1.0370
Q9UHD8	Septin-9	Sep-09	14.8	1.0372
P00558;P07205	Phosphoglycerate kinase 1	PGK1	53	1.0377
P29692	Elongation factor 1-delta	EEF1D	25.3	1.0377
P25788	Proteasome subunit alpha type-3	PSMA3	14.1	1.0386
Q15907;P62491	Ras-related protein Rab-11B;Ras-related protein Rab-11A	RAB11B;RAB11A	42.7	1.0388
P22626	Heterogeneous nuclear ribonucleoproteins A2/B1	HNRNPA2B1	13	1.0399
P07437	Tubulin beta chain	TUBB	27	1.0401
A6NIZ1;P61224	Ras-related protein Rap-1b-like protein;Ras-related protein Rap-1b	RAP1B	28.8	1.0402
O00299	Chloride intracellular channel protein 1	CLIC1	49.8	1.0406
P61978	Heterogeneous nuclear ribonucleoprotein K	HNRNPK	28.3	1.0410
P55884	Eukaryotic translation initiation factor 3 subunit B	EIF3B	10	1.0411
P52209	6-phosphogluconate dehydrogenase, decarboxylating	PGD	18.2	1.0416
P17302	Gap junction alpha-1 protein	GJA1	10.7	1.0416
P32969	60S ribosomal protein L9	RPL9	35.9	1.0417
P68036	Ubiquitin-conjugating enzyme E2 L3	UBE2L3	27.3	1.0419
P40926	Malate dehydrogenase, mitochondrial	MDH2	38.2	1.0424
O43776	Asparagine--tRNA ligase, cytoplasmic	NARS	12.6	1.0431
Q99714	3-hydroxyacyl-CoA dehydrogenase type-2	HSD17B10	34.1	1.0434
Q04446	1,4-alpha-glucan-branching enzyme	GBE1	14.4	1.0437
Q9NZN4	EH domain-containing protein 2	EHD2	16.6	1.0438
P07910;O60812	Heterogeneous nuclear ribonucleoproteins C1/C2;Heterogeneous nuclear ribonucleoprotein C-like 1	HNRNPC;HNRNPCL1	16.3	1.0438
P05387	60S acidic ribosomal protein P2	RPLP2	53.9	1.0440
P62937;A2BFH1;Q9Y536	Peptidyl-prolyl cis-trans isomerase A	PPIA	43.6	1.0447
Q58FF8	Putative heat shock protein HSP 90-beta 2	HSP90AB2P	8.9	1.0449
P46939	Utrophin	UTRN	1.3	1.0452

P62266	40S ribosomal protein S23	RPS23	39.9	1.0456
Q15717	ELAV-like protein 1	ELAVL1	17.2	1.0462
P55263	Adenosine kinase	ADK	21.8	1.0462
P55036	26S proteasome non-ATPase regulatory subunit 4	PSMD4	8.8	1.0464
P39019	40S ribosomal protein S19	RPS19	24.1	1.0464
P26885	Peptidyl-prolyl cis-trans isomerase FKBP2	FKBP2	29.6	1.0465
O00487	26S proteasome non-ATPase regulatory subunit 14	PSMD14	23.5	1.0470
O00560	Syntenin-1	SDCBP	14.8	1.0472
P05388;Q8NHW5	60S acidic ribosomal protein P0;60S acidic ribosomal protein P0-like	RPLP0;RPLP0P6	51.1	1.0474
Q9UMS4	Pre-mRNA-processing factor 19	PRPF19	23.2	1.0478
P11413	Glucose-6-phosphate 1-dehydrogenase	G6PD	29.3	1.0482
Q14764	Major vault protein	MVP	27	1.0482
P53597	Succinyl-CoA ligase [ADP/GDP-forming] subunit alpha, mitochondrial	SUCLG1	11.3	1.0495
Q7L2H7	Eukaryotic translation initiation factor 3 subunit M	EIF3M	20.9	1.0495
O95336	6-phosphogluconolactonase	PGLS	13.2	1.0499
Q9UMX0	Ubiquilin-1	UBQLN1	5.1	1.0502
Q9UN86	Ras GTPase-activating protein-binding protein 2	G3BP2	7.7	1.0505
Q14257	Reticulocalbin-2	RCN2	12.9	1.0511
Q16531	DNA damage-binding protein 1	DDB1	8.4	1.0517
P61106	Ras-related protein Rab-14	RAB14	28.4	1.0519
Q13155	Aminoacyl tRNA synthase complex-interacting multifunctional protein 2	AIMP2	6.9	1.0523
P00505	Aspartate aminotransferase, mitochondrial	GOT2	18.8	1.0530
Q9Y265	RuvB-like 1	RUVBL1	11.2	1.0531
P50914	60S ribosomal protein L14	RPL14	10.2	1.0543
P50995	Annexin A11	ANXA11	6.5	1.0547
P49748	Very long-chain specific acyl-CoA dehydrogenase, mitochondrial	ACADVL	14.5	1.0547
Q01970	1-phosphatidylinositol 4,5-bisphosphate phosphodiesterase beta-3	PLCB3	3.7	1.0550
P04899;P63096	Guanine nucleotide-binding protein G(i) subunit alpha-2;Guanine nucleotide-binding protein G(i) subunit alpha-1	GNAI2;GNAI1	33.5	1.0552
Q08AM6	Protein VAC14 homolog	VAC14	5.4	1.0556
P62280	40S ribosomal protein S11	RPS11	27.8	1.0558

P07900;Q14568;Q58FG0;Q58FG1	Heat shock protein HSP 90-alpha	HSP90AA1	28.1	1.0563
P16949;Q93045	Stathmin	STMN1	22.1	1.0570
Q13325	Interferon-induced protein with tetratricopeptide repeats 5	IFIT5	5.6	1.0574
O75947	ATP synthase subunit d, mitochondrial	ATP5H	32.9	1.0584
Q06210;O94808	Glucosamine--fructose-6-phosphate aminotransferase [isomerizing] 1	GFPT1	12.4	1.0601
P62333	26S protease regulatory subunit 10B	PSMC6	28.5	1.0602
Q00610;P53675	Clathrin heavy chain 1	CLTC	31.2	1.0603
Q9NRV9	Heme-binding protein 1	HEBP1	16.9	1.0609
P61163	Alpha-centractin	ACTR1A	23.7	1.0612
Q9Y394	Dehydrogenase/reductase SDR family member 7	DHRS7	15.9	1.0620
Q96QK1	Vacuolar protein sorting-associated protein 35	VPS35	9.7	1.0624
P48556	26S proteasome non-ATPase regulatory subunit 8	PSMD8	6.6	1.0624
P29144	Tripeptidyl-peptidase 2	TPP2	3.5	1.0629
Q14203	Dynactin subunit 1	DCTN1	4.5	1.0634
P62081	40S ribosomal protein S7	RPS7	52.1	1.0634
Q8NBS9	Thioredoxin domain-containing protein 5	TXNDC5	15.5	1.0641
P15880	40S ribosomal protein S2	RPS2	38.9	1.0643
P23246	Splicing factor, proline- and glutamine-rich	SFPQ	7.6	1.0647
Q86V81	THO complex subunit 4	ALYREF	12.1	1.0652
O60716	Catenin delta-1	CTNND1	6.2	1.0653
P51659	Peroxisomal multifunctional enzyme type 2;(3R)-hydroxyacyl-CoA dehydrogenase;Enoyl-CoA hydratase 2	HSD17B4	16.8	1.0654
P07195	L-lactate dehydrogenase B chain	LDHB	41.6	1.0669
Q96C19	EF-hand domain-containing protein D2	EFHD2	8.8	1.0673
P18206	Vinculin	VCL	27	1.0674
P62314	Small nuclear ribonucleoprotein Sm D1	SNRPD1	26.1	1.0676
P20290	Transcription factor BTF3	BTF3	56.3	1.0684
O75828	Carbonyl reductase [NADPH] 3	CBR3	28.2	1.0686
P11766	Alcohol dehydrogenase class-3	ADH5	41.2	1.0687

O75531	Barrier-to-autointegration factor	BANF1	42.7	1.0689
Q92900	Regulator of nonsense transcripts 1	UPF1	4.7	1.0693
Q9H488	GDP-fucose protein O-fucosyltransferase 1	POFUT1	10.8	1.0696
Q14697	Neutral alpha-glucosidase AB	GANAB	10.3	1.0700
Q01081;Q8WU68	Splicing factor U2AF 35 kDa subunit;Splicing factor U2AF 26 kDa subunit	U2AF1;U2AF1L4	14.2	1.0705
Q8WUM4	Programmed cell death 6-interacting protein	PDCD6IP	11.2	1.0707
O43390	Heterogeneous nuclear ribonucleoprotein R	HNRNPR	21.2	1.0710
P40123	Adenylyl cyclase-associated protein 2	CAP2	17.4	1.0712
P25786	Proteasome subunit alpha type-1	PSMA1	18.3	1.0714
P28066	Proteasome subunit alpha type-5	PSMA5	37.3	1.0718
Q14247	Src substrate cortactin	CTTN	22.2	1.0720
P51858	Hepatoma-derived growth factor	HDGF	22.1	1.0722
Q07020	60S ribosomal protein L18	RPL18	19.1	1.0723
O75368	SH3 domain-binding glutamic acid-rich-like protein	SH3BGRL	38.6	1.0725
P78559	Microtubule-associated protein 1A;MAP1 light chain LC2	MAP1A	3.7	1.0728
P13804	Electron transfer flavoprotein subunit alpha, mitochondrial	ETFA	46.5	1.0731
Q9BY44	Eukaryotic translation initiation factor 2A	EIF2A	5.8	1.0733
Q9H1E3	Nuclear ubiquitous casein and cyclin-dependent kinase substrate 1	NUCKS1	10.7	1.0740
Q9Y230	RuvB-like 2	RUVBL2	6	1.0740
P15170;Q8IYD1	Eukaryotic peptide chain release factor GTP-binding subunit ERF3A	GSPT1	12.6	1.0742
O14579	Coatomer subunit epsilon	COPE	20.5	1.0764
P63208	S-phase kinase-associated protein 1	SKP1	30.7	1.0784
Q02218;Q9ULD0	2-oxoglutarate dehydrogenase, mitochondrial	OGDH	12.6	1.0788
O60763	General vesicular transport factor p115	USO1	6.4	1.0792
P62873;Q9HAV0;P16520	Guanine nucleotide-binding protein G(I)/G(S)/G(T) subunit beta-1	GNB1	34.4	1.0797
P30048	Thioredoxin-dependent peroxide reductase, mitochondrial	PRDX3	30.1	1.0798
P0C7P4;P4	Putative cytochrome b-c1 complex subunit Rieske-like	UQCRFS	16.6	1.0800

7985	protein 1;Cytochrome b-c1 complex subunit Rieske, mitochondrial;Cytochrome b-c1 complex subunit 11	1P1;UQCRFS1		
Q15436;Q15437	Protein transport protein Sec23A	SEC23A	14.6	1.0811
P27695	DNA-(apurinic or apyrimidinic site) lyase;DNA-(apurinic or apyrimidinic site) lyase, mitochondrial	APEX1	9.4	1.0813
P14868	Aspartate--tRNA ligase, cytoplasmic	DARS	13.4	1.0817
Q96FW1	Ubiquitin thioesterase OTUB1	OTUB1	14.4	1.0819
P78344	Eukaryotic translation initiation factor 4 gamma 2	EIF4G2	3.7	1.0829
P30086	Phosphatidylethanolamine-binding protein 1;Hippocampal cholinergic neurostimulating peptide	PEBP1	49.7	1.0831
P30040	Endoplasmic reticulum resident protein 29	ERP29	23.8	1.0831
Q92896	Golgi apparatus protein 1	GLG1	3.5	1.0834
Q9H9B4	Sideroflexin-1	SFXN1	16.8	1.0838
P63010	AP-2 complex subunit beta	AP2B1	20.5	1.0844
P12956	X-ray repair cross-complementing protein 6	XRCC6	17.9	1.0857
Q03252	Lamin-B2	LMNB2	16.8	1.0858
P50502;Q8IZP2;Q8NFI4	Hsc70-interacting protein;Putative protein FAM10A4;Putative protein FAM10A5	ST13;ST13P4;ST13P5	17.9	1.0872
P31948	Stress-induced-phosphoprotein 1	STIP1	33.1	1.0873
P04792	Heat shock protein beta-1	HSPB1	31.7	1.0873
O43324	Eukaryotic translation elongation factor 1 epsilon-1	EEF1E1	20.7	1.0889
Q9NVD7;Q9HBI1	Alpha-parvin	PARVA	13.4	1.0893
P33176;Q12840;O60282	Kinesin-1 heavy chain	KIF5B	13.9	1.0905
Q16270	Insulin-like growth factor-binding protein 7	IGFBP7	11	1.0910
P36957	Dihydrolipoyllysine-residue succinyltransferase component of 2-oxoglutarate dehydrogenase complex, mitochondrial	DLST	9.5	1.0918
P50570;Q9UQ16;Q05193	Dynamin-2;Dynamin-3;Dynamin-1	DNM2;DNM3;DNM1	3.9	1.0918
O00571;O15523	ATP-dependent RNA helicase DDX3X;ATP-dependent RNA helicase DDX3Y	DDX3X;DDX3Y	15.3	1.0922

O00429	Dynamin-1-like protein	DNM1L	9.4	1.0923
Q9H0U4;Q92928	Ras-related protein Rab-1B	RAB1B	51.2	1.0926
P99999	Cytochrome c	CYCS	22.9	1.0940
P52272	Heterogeneous nuclear ribonucleoprotein M	HNRNPM	8.1	1.0944
P14314	Glucosidase 2 subunit beta	PRKCSH	22	1.0950
P00441	Superoxide dismutase [Cu-Zn]	SOD1	63	1.0951
O43399	Tumor protein D54	TPD52L2	15.5	1.0952
Q9BVK6	Transmembrane emp24 domain-containing protein 9	TMED9	13.2	1.0954
P31150	Rab GDP dissociation inhibitor alpha	GDI1	23.3	1.0954
P08238;Q58FF7	Heat shock protein HSP 90-beta	HSP90AB1	23.5	1.0956
P61604	10 kDa heat shock protein, mitochondrial	HSPE1	23.5	1.0960
Q12907	Vesicular integral-membrane protein VIP36	LMAN2	7.9	1.0985
P06753	Tropomyosin alpha-3 chain	TPM3	20.8	1.0985
P09622	Dihydrolipoyl dehydrogenase, mitochondrial	DLD	8.1	1.0987
P49773	Histidine triad nucleotide-binding protein 1	HINT1	27.8	1.0991
Q13148	TAR DNA-binding protein 43	TARDBP	8.7	1.1012
Q13423	NAD(P) transhydrogenase, mitochondrial	NNT	2.8	1.1020
P45880	Voltage-dependent anion-selective channel protein 2	VDAC2	42.9	1.1020
Q6NYC8	Phostensin	PPP1R18	9.1	1.1022
P18084	Integrin beta-5	ITGB5	3	1.1026
Q07954	Prolow-density lipoprotein receptor-related protein 1;Low-density lipoprotein receptor-related protein 1 85 kDa subunit;Low-density lipoprotein receptor-related protein 1 515 kDa subunit;Low-density lipoprotein receptor-related protein 1 intracellular domain	LRP1	1.8	1.1030
P67809;Q9Y2T7	Nuclease-sensitive element-binding protein 1	YBX1	45.4	1.1031
P13010	X-ray repair cross-complementing protein 5	XRCC5	30.9	1.1038
O00232	26S proteasome non-ATPase regulatory subunit 12	PSMD12	10.5	1.1039
P23284	Peptidyl-prolyl cis-trans isomerase B	PPIB	38.9	1.1050
Q9NY33	Dipeptidyl peptidase 3	DPP3	4.5	1.1053
Q6P2Q9	Pre-mRNA-processing-splicing factor 8	PRPF8	1.7	1.1054
Q13561	Dynactin subunit 2	DCTN2	32.4	1.1057
Q9Y678;Q9UBF2	Coatomer subunit gamma-1	COPG1	31.1	1.1060

Q9Y262	Eukaryotic translation initiation factor 3 subunit L	EIF3L	16.5	1.1065
P25789	Proteasome subunit alpha type-4	PSMA4	24.9	1.1066
P62249	40S ribosomal protein S16	RPS16	12.3	1.1067
P61088;Q5JXB2	Ubiquitin-conjugating enzyme E2 N	UBE2N	34.2	1.1069
O43681	ATPase ASNA1	ASNA1	11.2	1.1085
P61421	V-type proton ATPase subunit d 1	ATP6V0D1	6.6	1.1099
P08123	Collagen alpha-2(I) chain	COL1A2	7	1.1101
P20700	Lamin-B1	LMNB1	8.9	1.1111
O15173	Membrane-associated progesterone receptor component 2	PGRMC2	18.4	1.1134
P26599;O95758	Polypyrimidine tract-binding protein 1	PTBP1	23.9	1.1136
P60468	Protein transport protein Sec61 subunit beta	SEC61B	21.9	1.1139
Q14240	Eukaryotic initiation factor 4A-II	EIF4A2	15	1.1145
Q9Y305	Acyl-coenzyme A thioesterase 9, mitochondrial	ACOT9	5.2	1.1147
P42224	Signal transducer and activator of transcription 1-alpha/beta	STAT1	2.8	1.1149
Q14444	Caprin-1	CAPRIN1	17.8	1.1156
Q99729	Heterogeneous nuclear ribonucleoprotein A/B	HNRNPAB	6.3	1.1164
Q96KP4	Cytosolic non-specific dipeptidase	CNDP2	27.2	1.1165
P41091;Q2VIR3	Eukaryotic translation initiation factor 2 subunit 3;Putative eukaryotic translation initiation factor 2 subunit 3-like protein	EIF2S3;EIF2S3L	17.8	1.1167
Q00839	Heterogeneous nuclear ribonucleoprotein U	HNRNPU	21.1	1.1177
Q9NRX4	14 kDa phosphohistidine phosphatase	PHPT1	41.6	1.1187
O76094	Signal recognition particle 72 kDa protein	SRP72	7.5	1.1191
P16401	Histone H1.5	HIST1H1B	17.3	1.1203
Q9UBQ0	Vacuolar protein sorting-associated protein 29	VPS29	14.3	1.1209
Q9Y2X3	Nucleolar protein 58	NOP58	6.8	1.1214
P49419	Alpha-aminoadipic semialdehyde dehydrogenase	ALDH7A1	7.6	1.1221
P42025	Beta-centractin	ACTR1B	17.8	1.1228
P09382	Galectin-1	LGALS1	51.1	1.1229

P51149	Ras-related protein Rab-7a	RAB7A	36.7	1.1233
P07237	Protein disulfide-isomerase	P4HB	40.4	1.1240
Q9UBG0	C-type mannose receptor 2	MRC2	5.1	1.1241
Q9BRF8	Calcineurin-like phosphoesterase domain-containing protein 1	CPPED1	7	1.1253
Q08211	ATP-dependent RNA helicase A	DHX9	14.4	1.1264
Q15435	Protein phosphatase 1 regulatory subunit 7	PPP1R7	15.8	1.1268
Q96A49	Synapse-associated protein 1	SYAP1	12.5	1.1273
P78417	Glutathione S-transferase omega-1	GSTO1	51.9	1.1276
P00367;P49448	Glutamate dehydrogenase 1, mitochondrial;Glutamate dehydrogenase 2, mitochondrial	GLUD1;GLUD2	16.5	1.1276
P61289	Proteasome activator complex subunit 3	PSME3	13.8	1.1289
Q9ULA0	Aspartyl aminopeptidase	DNPEP	10.9	1.1300
P61009	Signal peptidase complex subunit 3	SPCS3	12.8	1.1301
P18669;Q8N0Y7;P15259	Phosphoglycerate mutase 1	PGAM1	31.1	1.1303
Q9Y6G9	Cytoplasmic dynein 1 light intermediate chain 1	DYNC1LI1	6.3	1.1305
P46940;Q86VI3;Q13576	Ras GTPase-activating-like protein IQGAP1	IQGAP1	34	1.1309
O14974	Protein phosphatase 1 regulatory subunit 12A	PPP1R12A	9	1.1313
P39656	Dolichyl-diphosphooligosaccharide--protein glycosyltransferase 48 kDa subunit	DDOST	21.7	1.1314
P35998	26S protease regulatory subunit 7	PSMC2	17.8	1.1327
Q9UBT2	SUMO-activating enzyme subunit 2	UBA2	5.2	1.1330
Q9UNH7	Sorting nexin-6	SNX6	5.4	1.1335
P08670;P14136;Q16352;P17661;P12036	Vimentin	VIM	51.3	1.1350
O43237	Cytoplasmic dynein 1 light intermediate chain 2	DYNC1LI2	15	1.1354
P27824	Calnexin	CANX	12.3	1.1357
Q9Y3I0	tRNA-splicing ligase RtcB homolog	C22orf28	13.3	1.1365
Q93052	Lipoma-preferred partner	LPP	13.2	1.1372

P02462	Collagen alpha-1(IV) chain;Arresten	COL4A1	0.9	1.1378
Q86VP6	Cullin-associated NEDD8-dissociated protein 1	CAND1	6.6	1.1387
P49189	4-trimethylaminobutyraldehyde dehydrogenase	ALDH9A1	19.8	1.1395
Q16851	UTP--glucose-1-phosphate uridylyltransferase	UGP2	13.4	1.1405
P36871	Phosphoglucomutase-1	PGM1	4.8	1.1423
Q14192	Four and a half LIM domains protein 2	FHL2	39.4	1.1423
Q92841	Probable ATP-dependent RNA helicase DDX17	DDX17	12.9	1.1452
Q14204	Cytoplasmic dynein 1 heavy chain 1	DYNC1H1	11.1	1.1456
A0AVT1	Ubiquitin-like modifier-activating enzyme 6	UBA6	2.9	1.1465
Q9UL46	Proteasome activator complex subunit 2	PSME2	23.8	1.1468
P62854;Q5JNZ5	40S ribosomal protein S26	RPS26	31.3	1.1481
P12236	ADP/ATP translocase 3	SLC25A6	31.5	1.1490
Q9NQ88	Probable fructose-2,6-bisphosphatase TIGAR	TIGAR	13	1.1491
Q99733	Nucleosome assembly protein 1-like 4	NAP1L4	19.5	1.1495
P60174	Triosephosphate isomerase	TPI1	51.7	1.1495
Q92598	Heat shock protein 105 kDa	HSPH1	25.6	1.1498
P09211	Glutathione S-transferase P	GSTP1	47.6	1.1502
P48444	Coatomer subunit delta	ARCN1	14.3	1.1502
Q13283	Ras GTPase-activating protein-binding protein 1	G3BP1	19.7	1.1502
P62979;P0CG48;P0CG47;P62987	Ubiquitin-40S ribosomal protein S27a;Ubiquitin;40S ribosomal protein S27a;Polyubiquitin-C;Ubiquitin;Polyubiquitin-B;Ubiquitin;Ubiquitin-60S ribosomal protein L40;Ubiquitin;60S ribosomal protein L40	RPS27A;UBC;UBB;UBA52	35.9	1.1514
P40121	Macrophage-capping protein	CAPG	20.7	1.1515
P25705	ATP synthase subunit alpha, mitochondrial	ATP5A1	16.8	1.1526
Q04637;O43432	Eukaryotic translation initiation factor 4 gamma 1	EIF4G1	6.9	1.1526
O14617	AP-3 complex subunit delta-1	AP3D1	4.2	1.1536
P52815	39S ribosomal protein L12, mitochondrial	MRPL12	23.2	1.1540
P35606	Coatomer subunit beta	COPB2	13.9	1.1560
P35579;P35749	Myosin-9	MYH9	34.6	1.1567
Q8IWE2	Protein NOXP20	FAM114	6.4	1.1609

		A1		
P31943;P55795	Heterogeneous nuclear ribonucleoprotein H;Heterogeneous nuclear ribonucleoprotein H, N-terminally processed	HNRNPH1	12.9	1.1613
P13489	Ribonuclease inhibitor	RNH1	13.7	1.1626
P20042	Eukaryotic translation initiation factor 2 subunit 2	EIF2S2	9.9	1.1628
P61960	Ubiquitin-fold modifier 1	UFM1	41.2	1.1634
Q13045	Protein flightless-1 homolog	FLII	3.5	1.1641
P14550	Alcohol dehydrogenase [NADP(+)]	AKR1A1	22.2	1.1647
Q9Y224	UPF0568 protein C14orf166	C14orf166	26.2	1.1657
Q00688	Peptidyl-prolyl cis-trans isomerase FKBP3	FKBP3	17.9	1.1677
P19367	Hexokinase-1	HK1	7.4	1.1680
Q1KMD3	Heterogeneous nuclear ribonucleoprotein U-like protein 2	HNRNPUL2	8	1.1686
P49902	Cytosolic purine 5-nucleotidase	NT5C2	10.7	1.1700
O14950;P19105;P24844	Myosin regulatory light chain 12B;Myosin regulatory light chain 12A;Myosin regulatory light polypeptide 9	MYL12B; MYL12A; MYL9	38.4	1.1711
P38646	Stress-70 protein, mitochondrial	HSPA9	22.7	1.1715
P40939	Trifunctional enzyme subunit alpha, mitochondrial;Long-chain enoyl-CoA hydratase;Long chain 3-hydroxyacyl-CoA dehydrogenase	HADHA	24.2	1.1750
P31949	Protein S100-A11	S100A11	49.5	1.1754
P32119	Peroxiredoxin-2	PRDX2	49	1.1785
Q9P2J5	Leucine--tRNA ligase, cytoplasmic	LARS	2.6	1.1789
P48735	Isocitrate dehydrogenase [NADP], mitochondrial	IDH2	11.7	1.1792
P30419;O60551	Glycylpeptide N-tetradecanoyltransferase 1;Glycylpeptide N-tetradecanoyltransferase 2	NMT1;NMT2	5.4	1.1797
P36405	ADP-ribosylation factor-like protein 3	ARL3	16.5	1.1863
P40925	Malate dehydrogenase, cytoplasmic	MDH1	16.8	1.1864
Q9BXJ9	N-alpha-acetyltransferase 15, NatA auxiliary subunit	NAA15	1.8	1.1875
P55060	Exportin-2	CSE1L	10.4	1.1882
Q15366;P57721	Poly(rC)-binding protein 2	PCBP2	21.9	1.1899
O76003	Glutaredoxin-3	GLRX3	14	1.1908
P53621	Coatomer subunit alpha;Xenin;Proxenin	COPA	11	1.1910

P62244	40S ribosomal protein S15a	RPS15A	18.5	1.1927
Q12905	Interleukin enhancer-binding factor 2	ILF2	26.2	1.1933
Q13596	Sorting nexin-1	SNX1	4.8	1.1939
P62879	Guanine nucleotide-binding protein G(I)/G(S)/G(T) subunit beta-2	GNB2	21.8	1.1987
Q04837	Single-stranded DNA-binding protein, mitochondrial	SSBP1	22.3	1.1988
Q9Y277	Voltage-dependent anion-selective channel protein 3	VDAC3	29.3	1.2001
Q9BS26	Endoplasmic reticulum resident protein 44	ERP44	13.5	1.2003
P31930	Cytochrome b-c1 complex subunit 1, mitochondrial	UQCRC1	10	1.2012
Q6NUK1	Calcium-binding mitochondrial carrier protein SCaMC-1	SLC25A24	6.9	1.2019
Q93008	Probable ubiquitin carboxyl-terminal hydrolase FAF-X	USP9X	1.4	1.2036
Q8NHP1;O43488	Aflatoxin B1 aldehyde reductase member 4;Aflatoxin B1 aldehyde reductase member 2	AKR7L;AKR7A2	9.7	1.2047
P05198	Eukaryotic translation initiation factor 2 subunit 1	EIF2S1	6	1.2062
O75390	Citrate synthase, mitochondrial	CS	11.2	1.2070
P06576	ATP synthase subunit beta, mitochondrial	ATP5B	36.3	1.2080
Q8WUP2	Filamin-binding LIM protein 1	FBLIM1	18.2	1.2083
Q12906;Q96SI9	Interleukin enhancer-binding factor 3	ILF3	13.5	1.2093
Q9BTV4	Transmembrane protein 43	TMEM43	9.5	1.2098
P60660;P14649	Myosin light polypeptide 6	MYL6	33.1	1.2102
Q14108	Lysosome membrane protein 2	SCARB2	11.5	1.2108
P13073	Cytochrome c oxidase subunit 4 isoform 1, mitochondrial	COX4I1	13	1.2117
Q13177;O75914;Q13153	Serine/threonine-protein kinase PAK 2;PAK-2p27;PAK-2p34;Serine/threonine-protein kinase PAK 3	PAK2;PAK3	9.7	1.2126
P53618	Coatomer subunit beta	COPB1	11.8	1.2126
O14737	Programmed cell death protein 5	PDCD5	20	1.2145
P49458	Signal recognition particle 9 kDa protein	SRP9	22.1	1.2146
Q13310	Polyadenylate-binding protein 4	PABPC4	9.9	1.2178
O60488	Long-chain-fatty-acid--CoA ligase 4	ACSL4	9.4	1.2199
P55084	Trifunctional enzyme subunit beta, mitochondrial;3-ketoacyl-CoA thiolase	HADHB	21.1	1.2208
P20810	Calpastatin	CAST	8.5	1.2215

Q27J81	Inverted formin-2	INF2	8.2	1.2220
Q13620	Cullin-4B	CUL4B	3.5	1.2224
Q16698	2,4-dienoyl-CoA reductase, mitochondrial	DECR1	11.6	1.2224
Q14498	RNA-binding protein 39	RBM39	4.9	1.2239
Q16555	Dihydropyrimidinase-related protein 2	DPYSL2	36.5	1.2244
P28074	Proteasome subunit beta type-5	PSMB5	22.8	1.2269
P52565	Rho GDP-dissociation inhibitor 1	ARHGDI A	31.4	1.2274
Q9H444	Charged multivesicular body protein 4b	CHMP4B	29.9	1.2276
Q9HC38	Glyoxalase domain-containing protein 4	GLOD4	11.5	1.2281
Q96TA1	Niban-like protein 1	FAM129 B	18.2	1.2281
O60664	Perilipin-3	PLIN3	24.9	1.2350
P07384	Calpain-1 catalytic subunit	CAPN1	8	1.2362
P50583	Bis(5-nucleosyl)-tetraphosphatase [asymmetrical]	NUDT2	40.1	1.2406
Q12792	Twinfilin-1	TWF1	5.7	1.2408
P02751	Fibronectin;Anastellin;Ugl-Y1;Ugl-Y2;Ugl-Y3	FN1	10.1	1.2414
P61923	Coatomer subunit zeta-1	COPZ1	13.6	1.2418
P20073	Annexin A7	ANXA7	6.1	1.2419
P30044	Peroxiredoxin-5, mitochondrial	PRDX5	30.4	1.2428
Q96HC4	PDZ and LIM domain protein 5	PDLIM5	20.8	1.2432
P61586;P0 8134	Transforming protein RhoA;Rho-related GTP-binding protein RhoC	RHOA;R HOC	20.2	1.2434
Q96M27	Protein PRRC1	PRRC1	11.2	1.2475
P09429;B2 RPK0	High mobility group protein B1	HMGB1	26	1.2478
Q15363	Transmembrane emp24 domain-containing protein 2	TMED2	19.9	1.2494
P08473	Neprilysin	MME	7.1	1.2504
Q16891	Mitochondrial inner membrane protein	IMMT	12.8	1.2520
Q07866;Q 9H0B6	Kinesin light chain 1	KLC1	15.5	1.2527
Q96CW1	AP-2 complex subunit mu	AP2M1	8.3	1.2615
P06396;C ON__Q3S X14	Gelsolin	GSN	21.6	1.2627
P48059	LIM and senescent cell antigen-like-containing domain protein 1	LIMS1	13.2	1.2674

Q14195;Q14194	Dihydropyrimidinase-related protein 3	DPYSL3	27.7	1.2676
P47897	Glutamine--tRNA ligase	QARS	7.4	1.2699
P26447	Protein S100-A4	S100A4	27.7	1.2711
O75439	Mitochondrial-processing peptidase subunit beta	PMPCB	8.6	1.2756
P27797	Calreticulin	CALR	52.5	1.2759
O00151	PDZ and LIM domain protein 1	PDLIM1	37.7	1.2779
P11216	Glycogen phosphorylase, brain form	PYGB	7.9	1.2808
P17174	Aspartate aminotransferase, cytoplasmic	GOT1	16.5	1.2810
P07858	Cathepsin B;Cathepsin B light chain;Cathepsin B heavy chain	CTSB	25.7	1.2843
P56192	Methionine--tRNA ligase, cytoplasmic	MARS	4.1	1.2846
P18085;P84085	ADP-ribosylation factor 4;ADP-ribosylation factor 5	ARF4;ARF5	31.7	1.2848
P23396	40S ribosomal protein S3	RPS3	28	1.2864
Q9Y2Z0	Suppressor of G2 allele of SKP1 homolog	SUGT1	12.9	1.2866
Q15365	Poly(rC)-binding protein 1	PCBP1	27	1.2890
P17655	Calpain-2 catalytic subunit	CAPN2	28.6	1.2900
P61769	Beta-2-microglobulin;Beta-2-microglobulin form pI 5.3	B2M	46.2	1.2946
Q9NYU2	UDP-glucose:glycoprotein glucosyltransferase 1	UGGT1	8.5	1.3078
P40616	ADP-ribosylation factor-like protein 1	ARL1	13.8	1.3114
P62328	Thymosin beta-4;Hematopoietic system regulatory peptide	TMSB4X	61.4	1.3177
P00491	Purine nucleoside phosphorylase	PNP	26.6	1.3185
P27361	Mitogen-activated protein kinase 3	MAPK3	15	1.3193
P62318	Small nuclear ribonucleoprotein Sm D3	SNRPD3	20.6	1.3200
Q8NI22	Multiple coagulation factor deficiency protein 2	MCFD2	44.5	1.3206
Q99798	Aconitate hydratase, mitochondrial	ACO2	9.6	1.3217
Q14008	Cytoskeleton-associated protein 5	CKAP5	2.4	1.3237
P49023	Paxillin	PXN	8.1	1.3254
P18859	ATP synthase-coupling factor 6, mitochondrial	ATP5J	26.9	1.3268
P61011	Signal recognition particle 54 kDa protein	SRP54	6.3	1.3300
P21796	Voltage-dependent anion-selective channel protein 1	VDAC1	52.3	1.3320
Q13409	Cytoplasmic dynein 1 intermediate chain 2	DYNC1I2	14.9	1.3360
Q13435	Splicing factor 3B subunit 2	SF3B2	6.9	1.3372
P61019;Q8WUD1	Ras-related protein Rab-2A	RAB2A	18.4	1.3395

P18124	60S ribosomal protein L7	RPL7	23.8	1.3426
Q9Y333	U6 snRNA-associated Sm-like protein LSm2	LSM2	28.4	1.3429
O75489	NADH dehydrogenase [ubiquinone] iron-sulfur protein 3, mitochondrial	NDUFS3	11	1.3497
P39748	Flap endonuclease 1	FEN1	9.5	1.3572
P80303	Nucleobindin-2	NUCB2	11.7	1.3579
P38159;O75526;Q96E39	RNA-binding motif protein, X chromosome;RNA-binding motif protein, X chromosome, N-terminally processed;RNA-binding motif protein, X-linked-like-2;RNA binding motif protein, X-linked-like-1;RNA binding motif protein, X-linked-like-1, N-terminally processed	RBMX;RBMXL2;RBMXL1	9.5	1.3582
O75874	Isocitrate dehydrogenase [NADP] cytoplasmic	IDH1	20.5	1.3631
Q06323	Proteasome activator complex subunit 1	PSME1	23.7	1.3685
P19404	NADH dehydrogenase [ubiquinone] flavoprotein 2, mitochondrial	NDUFV2	9.2	1.3749
O00203;Q13367	AP-3 complex subunit beta-1	AP3B1	3	1.3794
P24539	ATP synthase subunit b, mitochondrial	ATP5F1	23.8	1.3819
Q14157	Ubiquitin-associated protein 2-like	UBAP2L	9.2	1.3905
P17301	Integrin alpha-2	ITGA2	13.2	1.3907
O60814;P57053;P58876;P62807;Q5QNW6;Q93079;Q99877;Q99879;Q99880;Q96A08	Histone H2B type 1-K;Histone H2B type F-S;Histone H2B type 1-D;Histone H2B type 1-C/E/F/G/I;Histone H2B type 2-F;Histone H2B type 1-H;Histone H2B type 1-N;Histone H2B type 1-M;Histone H2B type 1-L	HIST1H2BK;H2BFS;HIST1H2BD;HIST1H2BC;HIST2H2BF;HIST1H2BH;HIST1H2BN;HIST1H2BM;HIST1H2BL	28.6	1.3954
P12111	Collagen alpha-3(VI) chain	COL6A3	10.1	1.3965
Q15084	Protein disulfide-isomerase A6	PDIA6	17.3	1.3979
Q9HCN8	Stromal cell-derived factor 2-like protein 1	SDF2L1	14.9	1.4072
P15586	N-acetylglucosamine-6-sulfatase	GNS	7.2	1.4073
P07942	Laminin subunit beta-1	LAMB1	4.6	1.4127

P07602	Proactivator polypeptide;Saposin-A;Saposin-B-Val;Saposin-B;Saposin-C;Saposin-D	PSAP	17	1.4146
P06703	Protein S100-A6	S100A6	55.6	1.4150
P55145	Mesencephalic astrocyte-derived neurotrophic factor	MANF	25.3	1.4178
P20020;Q01814;Q16720	Plasma membrane calcium-transporting ATPase 1	ATP2B1	8.6	1.4194
P21980	Protein-glutamine gamma-glutamyltransferase 2	TGM2	28.4	1.4200
P01891;P01892;P05534;P10316;P30447;P04439;P10314;P13746;P16188;P16189;P16190;P18462;P30443;P30450;P30453;P30455;P30456;P30457;P30459;P30512;Q09160;P01889;P03989;P10319;P18463;P18465;P30461;P30462;P30464;P30466;P30475;P30479;P30480;P30481;P30483;P30484;P30485;P30486;P304	HLA class I histocompatibility antigen, A-68 alpha chain;HLA class I histocompatibility antigen, A-2 alpha chain;HLA class I histocompatibility antigen, A-24 alpha chain;HLA class I histocompatibility antigen, A-69 alpha chain;HLA class I histocompatibility antigen, A-23 alpha chain;HLA class I histocompatibility antigen, A-3 alpha chain;HLA class I histocompatibility antigen, A-32 alpha chain;HLA class I histocompatibility antigen, A-11 alpha chain;HLA class I histocompatibility antigen, A-30 alpha chain;HLA class I histocompatibility antigen, A-31 alpha chain;HLA class I histocompatibility antigen, A-33 alpha chain;HLA class I histocompatibility antigen, A-25 alpha chain;HLA class I histocompatibility antigen, A-1 alpha chain;HLA class I histocompatibility antigen, A-26 alpha chain;HLA class I histocompatibility antigen, A-34 alpha chain;HLA class I histocompatibility antigen, A-36 alpha chain;HLA class I histocompatibility antigen, A-43 alpha chain;HLA class I histocompatibility antigen, A-66 alpha chain;HLA class I histocompatibility antigen, A-74 alpha chain;HLA class I histocompatibility antigen, A-29 alpha chain;HLA class I histocompatibility antigen, A-80 alpha chain;HLA class I histocompatibility antigen, B-7 alpha chain;HLA class I histocompatibility antigen, B-27 alpha chain;HLA class I histocompatibility antigen, B-58 alpha chain;HLA class I histocompatibility antigen, B-37 alpha chain;HLA	HLA-A;HLA-B	10.4	1.4281

87;P30488;P30490;P30492;P30493;P30495;Q04826;Q29718;Q29836;Q31610;Q95365	class I histocompatibility antigen, B-57 alpha chain;HLA class I histocompatibility antigen, B-13 alpha chain;HLA class I histocompatibility antigen, B-14 alpha chain;HLA class I histocompatibility antigen, B-15 alpha chain;HLA class I histocompatibility antigen, B-18 alpha chain;HLA class I histocompatibility antigen, B-39 alpha chain;HLA class I histocompatibility antigen, B-41 alpha chain;HLA class I histocompatibility antigen, B-42 alpha chain;HLA class I histocompatibility antigen, B-44 alpha chain;HLA class I histocompatibility antigen, B-45 alpha chain;HLA class I histocompatibility antigen, B-46 alpha chain;HLA class I histocompatibility antigen, B-47 alpha chain;HLA class I histocompatibility antigen, B-48 alpha chain;HLA class I histocompatibility antigen, B-49 alpha chain;HLA class I histocompatibility antigen, B-50 alpha chain;HLA class I histocompatibility antigen, B-52 alpha chain;HLA class I histocompatibility antigen, B-54 alpha chain;HLA class I histocompatibility antigen, B-55 alpha chain;HLA class I histocompatibility antigen, B-56 alpha chain;HLA class I histocompatibility antigen, B-40 alpha chain;HLA class I histocompatibility antigen, B-82 alpha chain;HLA class I histocompatibility antigen, B-67 alpha chain;HLA class I histocompatibility antigen, B-81 alpha chain;HLA class I histocompatibility antigen, B-38 alpha chain			
P04062	Glucosylceramidase	GBA	10.4	1.4292
P46821	Microtubule-associated protein 1B;MAP1 light chain LC1	MAP1B	3.6	1.4312
P30101	Protein disulfide-isomerase A3	PDIA3	27.5	1.4319
O60443	Non-syndromic hearing impairment protein 5	DFNA5	8.1	1.4395
P10412;P16402;Q02539;P22492	Histone H1.4;Histone H1.3;Histone H1.1;Histone H1t	HIST1H1E;HIST1H1D;HIST1H1A;HIST1H1T	12.3	1.4499

P21399	Cytoplasmic aconitate hydratase	ACO1	12.8	1.4556
P11047	Laminin subunit gamma-1	LAMC1	1.5	1.4571
P61204;P84077	ADP-ribosylation factor 3;ADP-ribosylation factor 1	ARF3;ARF1	35.4	1.4574
O95831	Apoptosis-inducing factor 1, mitochondrial	AIFM1	7.5	1.4590
P62805	Histone H4	HIST1H4A	30.1	1.4606
P04632;Q96L46	Calpain small subunit 1	CAPNS1	10.8	1.4648
Q96HE7	ERO1-like protein alpha	ERO1L	4.9	1.4660
P04908;P0C0S8;P20671;Q6FI13;Q7L7L0;Q93077;Q16777;Q9BTM1;Q96KK5;Q99878	Histone H2A type 1-B/E;Histone H2A type 1;Histone H2A type 1-D;Histone H2A type 2-A;Histone H2A type 3;Histone H2A type 1-C;Histone H2A type 2-C;Histone H2A.J;Histone H2A type 1-H;Histone H2A type 1-J	HIST1H2AB;HIST1H2AG;HIST1H2AD;HIST2H2AA3;HIST3H2A;HIST1H2AC;HIST2H2AC;H2AFJ;HIST1H2AH;HIST1H2AJ	14.6	1.4667
Q8WX93	Palladin	PALLD	11.9	1.4757
P23381	Tryptophan--tRNA ligase, cytoplasmic;T1-TrpRS;T2-TrpRS	WARS	34.4	1.4790
P15121	Aldose reductase	AKR1B1	36.4	1.5010
P50148;P29992;O95837	Guanine nucleotide-binding protein G(q) subunit alpha;Guanine nucleotide-binding protein subunit alpha-11;Guanine nucleotide-binding protein subunit alpha-14	GNAQ;GNA11;GNA14	10	1.5013
P62316	Small nuclear ribonucleoprotein Sm D2	SNRPD2	17.8	1.5174
P45974	Ubiquitin carboxyl-terminal hydrolase 5	USP5	8	1.5207
Q13509;CON__ENSEMBL:ENSBTAP000	Tubulin beta-3 chain	TUBB3	21.1	1.5216

00025008;<br>Q9H4B7;A<br>6NKZ8				
P07686	Beta-hexosaminidase subunit beta;Beta-hexosaminidase subunit beta chain B;Beta-hexosaminidase subunit beta chain A	HEXB	13.3	1.5249
Q99536	Synaptic vesicle membrane protein VAT-1 homolog	VAT1	26.2	1.5284
Q9Y3B3	Transmembrane emp24 domain-containing protein 7	TMED7	12.9	1.5387
P61916	Epididymal secretory protein E1	NPC2	61.6	1.5708
P16104;Q96QV6;Q8IUE6	Histone H2A.x;Histone H2A type 1-A;Histone H2A type 2-B	H2AFX;HIST1H2AA;HIST2H2AB	16.8	1.5798
P35580	Myosin-10	MYH10	12.4	1.6129
P17096	High mobility group protein HMG-I/HMG-Y	HMGA1	23.4	1.6148
P07339	Cathepsin D;Cathepsin D light chain;Cathepsin D heavy chain	CTSD	24.5	1.6252
P11021	78 kDa glucose-regulated protein	HSPA5	27.2	1.6583
Q9HD45	Transmembrane 9 superfamily member 3	TM9SF3	3.4	1.6701
P63098;Q96LZ3	Calcineurin subunit B type 1	PPP3R1	34.7	1.6969
P20618	Proteasome subunit beta type-1	PSMB1	14.1	1.7039
Q9Y4L1	Hypoxia up-regulated protein 1	HYOU1	14.6	1.7330
Q9Y5X1	Sorting nexin-9	SNX9	7.2	1.7413
P61201	COP9 signalosome complex subunit 2	COPS2	6.5	1.7612
Q6UXH1	Cysteine-rich with EGF-like domain protein 2	CRELD2	8.2	1.7720
P42126	Enoyl-CoA delta isomerase 1, mitochondrial	ECI1	12.3	1.8019
Q9H2U2	Inorganic pyrophosphatase 2, mitochondrial	PPA2	8.7	1.8262
P14625;Q58FF3	Endoplasmin	HSP90B1	33.3	1.8484
P43235	Cathepsin K	CTSK	12.2	1.8618
P55039	Developmentally-regulated GTP-binding protein 2	DRG2	5.5	1.8643
Q99715	Collagen alpha-1(XII) chain	COL12A1	1.5	1.8735
P07305	Histone H1.0	H1F0	11.9	1.8854
P27658	Collagen alpha-1(VIII) chain;Vastatin	COL8A1	3.2	1.9158
P61081	NEDD8-conjugating enzyme Ubc12	UBE2M	13.1	1.9296
Q13510	Acid ceramidase;Acid ceramidase subunit alpha;Acid	ASAH1	10.1	1.9855

	ceramidase subunit beta			
P13667	Protein disulfide-isomerase A4	PDIA4	27.1	2.0283
Q63ZY3	KN motif and ankyrin repeat domain-containing protein 2	KANK2	3.1	2.0810
Q14956	Transmembrane glycoprotein NMB	GPNMB	3.1	2.1011
Q6P2E9	Enhancer of mRNA-decapping protein 4	EDC4	3.4	2.1197
P43490	Nicotinamide phosphoribosyltransferase	NAMPT	22.4	2.2273
Q5EG05	Caspase recruitment domain-containing protein 16	CARD16	15.7	2.2439
P33993	DNA replication licensing factor MCM7	MCM7	4.6	2.3628
Q8NF91	Nesprin-1	SYNE1	0.4	2.5356
O75955	Flotillin-1	FLOT1	4.4	2.5398
P37235;P61601;P84074	Hippocalcin-like protein 1;Neurocalcin-delta;Neuron-specific calcium-binding protein hippocalcin	HPCAL1; NCALD; HPCA	10.4	2.6682
P21283	V-type proton ATPase subunit C 1	ATP6V1C1	7.3	2.8186
Q02978	Mitochondrial 2-oxoglutarate/malate carrier protein	SLC25A11	7	2.9796
O00505	Importin subunit alpha-3	KPNA3	6	3.1300
P20674	Cytochrome c oxidase subunit 5A, mitochondrial	COX5A	16.7	3.1870
Q9UNS2	COP9 signalosome complex subunit 3	COPS3	13.5	3.7121
Q9UKK9	ADP-sugar pyrophosphatase	NUDT5	15.1	3.7268
Q9NP97	Dynein light chain roadblock-type 1	DYNLRB1	27.1	4.4992

Supplementary Table S3

Protein IDs	Protein names	Gene names	Sequence coverage [%]	Ratio OCHA^L: YCHA^H
P00352	Retinal dehydrogenase 1	ALDH1A1	25.9	0.2200
P02452;CON__Q862S4	Collagen alpha-1(I) chain	COL1A1	13.8	0.3001
P17302	Gap junction alpha-1 protein	GJA1	14.4	0.3566
P49721	Proteasome subunit beta type-2	PSMB2	13.4	0.3834
P08195	4F2 cell-surface antigen heavy chain	SLC3A2	5.1	0.4233
Q13501	Sequestosome-1	SQSTM1	17.3	0.4261
Q8N3C0	Activating signal cointegrator 1 complex subunit 3	ASCC3	1.3	0.4373
P29966	Myristoylated alanine-rich C-kinase substrate	MARCKS	44.6	0.4451
P08123	Collagen alpha-2(I) chain	COL1A2	7.8	0.4687
Q99715	Collagen alpha-1(XII) chain	COL12A1	9.3	0.4788
Q15758	Neutral amino acid transporter B(0)	SLC1A5	6.5	0.5064
P26583	High mobility group protein B2	HMGB2	22.5	0.5072
P20700	Lamin-B1	LMNB1	7.3	0.5203
P50454	Serpin H1	SERPINH1	30.1	0.5379
P25325	3-mercaptopyruvate sulfurtransferase	MPST	14.5	0.5433
P09486	SPARC	SPARC	15.5	0.5516
O43175	D-3-phosphoglycerate dehydrogenase	PHGDH	19.7	0.5583
P16401	Histone H1.5	HIST1H1B	16.8	0.5656
P29279	Connective tissue growth factor	CTGF	5.4	0.5661
Q08945	FACT complex subunit SSRP1	SSRP1	5.1	0.5721
Q03252	Lamin-B2	LMNB2	14.7	0.5742
P10412;P16402;Q02539;P22492	Histone H1.4;Histone H1.3	HIST1H1E;HIST1H1D	20.1	0.5880
P09429;B2RPK0;P23497	High mobility group protein B1	HMGB1	26	0.5885
Q9Y680	Peptidyl-prolyl cis-trans isomerase FKBP7	FKBP7	13.1	0.5940
P34897	Serine hydroxymethyltransferase, mitochondrial	SHMT2	12.3	0.5956
Q13740	CD166 antigen	ALCAM	17.7	0.5984

O00148	ATP-dependent RNA helicase DDX39A	DDX39A	22.7	0.5991
P16403	Histone H1.2	HIST1H1C	18.3	0.6050
P21980	Protein-glutamine gamma-glutamyltransferase 2	TGM2	36.5	0.6050
O60814;P57053;P58876;P62807;Q5QNW6;Q93079;Q99877;Q99879;Q99880;P06899;P23527;P33778;Q16778;Q96A08;Q8N257	Histone H2B type 1-K;Histone H2B type F-S;Histone H2B type 1-D;Histone H2B type 1-C/E/F/G/I;Histone H2B type 2-F;Histone H2B type 1-H;Histone H2B type 1-N;Histone H2B type 1-M;Histone H2B type 1-L;Histone H2B type 1-J;Histone H2B type 1-O;Histone H2B type 1-B;Histone H2B type 2-E	HIST1H2BK;H2BFS;HIST1H2BD;HIST1H2BC;HIST2H2BF;HIST1H2BH;HIST1H2BN;HIST1H2BM;HIST1H2BL;HIST1H2BJ;HIST1H2BO;HIST1H2BB;HIST2H2BE	34.1	0.6225
P15559	NAD(P)H dehydrogenase [quinone] 1	NQO1	22.3	0.6251
P78344	Eukaryotic translation initiation factor 4 gamma 2	EIF4G2	5.4	0.6298
Q9NR30;Q9BQ39	Nucleolar RNA helicase 2	DDX21	6.4	0.6319
P39748	Flap endonuclease 1	FEN1	15	0.6322
P12004	Proliferating cell nuclear antigen	PCNA	29.5	0.6332
P04908;P0C0S8;P20671;Q6FI13;Q7L7L0;Q93077;Q16777;Q9BTM1;Q96KK5;Q99878	Histone H2A type 1-B/E;Histone H2A type 1;Histone H2A type 1-D;Histone H2A type 2-A;Histone H2A type 3;Histone H2A type 1-C;Histone H2A type 2-C;Histone H2A.J;Histone H2A type 1-H;Histone H2A type 1-J	HIST1H2AB;HIST1H2AG;HIST1H2AD;HIST2H2AA3;HIST3H2A;HIST1H2AC;HIST2H2AC;H	22.3	0.6371

		2AFJ;HIST1H2AH;HIST1H2AJ		
Q16576	Histone-binding protein RBBP7	RBBP7	18.8	0.6380
Q13185	Chromobox protein homolog 3	CBX3	30.1	0.6414
Q9UHB6	LIM domain and actin-binding protein 1	LIMA1	7.6	0.6461
P35659	Protein DEK	DEK	6.7	0.6518
P16104;Q96QV6;Q8IUE6	Histone H2A.x;Histone H2A type 1-A;Histone H2A type 2-B	H2AFX;HIST1H2AA;HIST2H2AB	23.1	0.6520
Q9Y570	Protein phosphatase methylesterase 1	PPME1	22	0.6521
P15311	Ezrin	EZR	26.6	0.6542
P45973	Chromobox protein homolog 5	CBX5	18.8	0.6542
P08648	Integrin alpha-5;Integrin alpha-5 heavy chain;Integrin alpha-5 light chain	ITGA5	5.6	0.6579
Q9UKM9	RNA-binding protein Raly	RALY	9.8	0.6648
P46063	ATP-dependent DNA helicase Q1	RECQL	5.7	0.6659
Q02790	Peptidyl-prolyl cis-trans isomerase FKBP4;Peptidyl-prolyl cis-trans isomerase FKBP4, N-terminally processed	FKBP4	33.6	0.6661
P22307	Non-specific lipid-transfer protein	SCP2	8	0.6665
Q99729	Heterogeneous nuclear ribonucleoprotein A/B	HNRNPAB	6.3	0.6712
Q9NZL4	Hsp70-binding protein 1	HSPBP1	4.7	0.6751
P62805	Histone H4	HIST1H4A	30.1	0.6766
O95340;O43252	Bifunctional 3-phosphoadenosine 5-phosphosulfate synthase 2;Sulfate adenylyltransferase;Adenylyl-sulfate kinase	PAPSS2	14.7	0.6821
P23219	Prostaglandin G/H synthase 1	PTGS1	5.5	0.6849
Q09028	Histone-binding protein RBBP4	RBBP4	20.2	0.6926
P35637;Q92804	RNA-binding protein FUS;TATA-binding protein-associated factor 2N	FUS;TAF15	4.8	0.6949
Q15427	Splicing factor 3B subunit 4	SF3B4	9.4	0.6957
Q07666	KH domain-containing, RNA-binding, signal transduction-associated protein 1	KHDRBS1	8.6	0.6962

P09651;Q32P51	Heterogeneous nuclear ribonucleoprotein A1;Heterogeneous nuclear ribonucleoprotein A1-like 2	HNRNPA1;HNRNPA1L2	14.2	0.6971
Q9HB71	Calcyclin-binding protein	CACYBP	32	0.7000
O15372	Eukaryotic translation initiation factor 3 subunit H	EIF3H	14.2	0.7038
P55060	Exportin-2	CSE1L	12.9	0.7042
Q01469;A8MUU1	Fatty acid-binding protein, epidermal;Putative fatty acid-binding protein 5-like protein 3	FABP5;FABP5P3	23.7	0.7050
P31943	Heterogeneous nuclear ribonucleoprotein H;Heterogeneous nuclear ribonucleoprotein H, N-terminally processed	HNRNPH1	9.4	0.7072
Q9BTT0	Acidic leucine-rich nuclear phosphoprotein 32 family member E	ANP32E	17.9	0.7090
P19338	Nucleolin	NCL	23.4	0.7092
Q86V81	THO complex subunit 4	ALYREF	12.1	0.7094
P49321	Nuclear autoantigenic sperm protein	NASP	8.9	0.7149
P06748	Nucleophosmin	NPM1	19.7	0.7169
P11233;P11234	Ras-related protein Ral-A;Ras-related protein Ral-B	RALA;RALB	11.7	0.7208
P20290	Transcription factor BTF3	BTF3	38.8	0.7217
P07910;O60812	Heterogeneous nuclear ribonucleoproteins C1/C2;Heterogeneous nuclear ribonucleoprotein C-like 1	HNRNPC;HNRNPCL1	19.9	0.7221
Q9Y617	Phosphoserine aminotransferase	PSAT1	16.5	0.7229
P55010	Eukaryotic translation initiation factor 5	EIF5	7.9	0.7261
O43670	Zinc finger protein 207	ZNF207	4.2	0.7303
P62316	Small nuclear ribonucleoprotein Sm D2	SNRPD2	26.3	0.7315
O14979	Heterogeneous nuclear ribonucleoprotein D-like	HNRPDL	16.9	0.7326
Q15029	116 kDa U5 small nuclear ribonucleoprotein component	EFTUD2	4.3	0.7327
P62280	40S ribosomal protein S11	RPS11	9.5	0.7330
Q9UMS4	Pre-mRNA-processing factor 19	PRPF19	18.7	0.7364
Q01081;Q8WU68	Splicing factor U2AF 35 kDa subunit;Splicing factor U2AF 26 kDa subunit	U2AF1;U2AF1L4	18.3	0.7369
Q00839	Heterogeneous nuclear ribonucleoprotein U	HNRNPU	19.4	0.7372
Q13151	Heterogeneous nuclear ribonucleoprotein A0	HNRNPA0	19.3	0.7401
Q07021	Complement component 1 Q	C1QBP	13.8	0.7410

	subcomponent-binding protein, mitochondrial			
Q15459	Splicing factor 3A subunit 1	SF3A1	2	0.7416
Q8NHP1;O43488	Aflatoxin B1 aldehyde reductase member 4;Aflatoxin B1 aldehyde reductase member 2	AKR7L;AKR7A2	9.7	0.7416
P42765	3-ketoacyl-CoA thiolase, mitochondrial	ACAA2	11.6	0.7426
P52597	Heterogeneous nuclear ribonucleoprotein F;Heterogeneous nuclear ribonucleoprotein F, N-terminally processed	HNRNPF	8	0.7428
P48681	Nestin	NES	5.7	0.7468
P23246	Splicing factor, proline- and glutamine-rich	SFPQ	9.9	0.7470
P62917	60S ribosomal protein L8	RPL8	17.1	0.7481
O00560	Syntenin-1	SDCBP	21.5	0.7487
P13674	Prolyl 4-hydroxylase subunit alpha-1	P4HA1	12.4	0.7517
O76021	Ribosomal L1 domain-containing protein 1	RSL1D1	8	0.7524
Q32P28	Prolyl 3-hydroxylase 1	LEPRE1	5.3	0.7527
P11586	C-1-tetrahydrofolate synthase, cytoplasmic;Methylenetetrahydrofolate dehydrogenase;Methenyltetrahydrofolate cyclohydrolase;Formyltetrahydrofolate synthetase	MTHFD1	13.2	0.7529
P17844	Probable ATP-dependent RNA helicase DDX5	DDX5	10.9	0.7533
Q13347	Eukaryotic translation initiation factor 3 subunit I	EIF3I	16.6	0.7541
P49458	Signal recognition particle 9 kDa protein	SRP9	47.7	0.7541
Q13162	Peroxiredoxin-4	PRDX4	19.6	0.7563
Q96AE4	Far upstream element-binding protein 1	FUBP1	12.6	0.7565
P54886	Delta-1-pyrroline-5-carboxylate synthase;Glutamate 5-kinase;Gamma-glutamyl phosphate reductase	ALDH18A1	3.9	0.7612
Q16527	Cysteine and glycine-rich protein 2	CSRP2	14	0.7629
O95881	Thioredoxin domain-containing protein 12	TXNDC12	22.1	0.7634
P38919	Eukaryotic initiation factor 4A-III	EIF4A3	11.4	0.7638
P10809	60 kDa heat shock protein, mitochondrial	HSPD1	34.7	0.7650
Q92945	Far upstream element-binding protein 2	KHSRP	8.6	0.7672
O00505	Importin subunit alpha-3	KPNA3	8.1	0.7680
A6NIZ1;P61224;P62834	Ras-related protein Rap-1b-like protein;Ras-related protein Rap-1b	RAP1B	22.3	0.7704
O95433	Activator of 90 kDa heat shock protein ATPase	AHSA1	7.7	0.7733

	homolog 1			
Q00341	Vigilin	HDLBP	15.6	0.7769
P49915	GMP synthase [glutamine-hydrolyzing]	GMPS	6.1	0.7769
P43487	Ran-specific GTPase-activating protein	RANBP1	15.4	0.7779
O75533	Splicing factor 3B subunit 1	SF3B1	6.1	0.7785
Q9UHX1	Poly(U)-binding-splicing factor PUF60	PUF60	7.7	0.7795
P50281	Matrix metalloproteinase-14	MMP14	7.4	0.7798
P14866;Q8WVV9	Heterogeneous nuclear ribonucleoprotein L	HNRNPL	26.1	0.7816
P60842;Q14240	Eukaryotic initiation factor 4A-I	EIF4A1	24.9	0.7816
P50914	60S ribosomal protein L14	RPL14	10.2	0.7819
P49588	Alanine--tRNA ligase, cytoplasmic	AARS	13.1	0.7820
Q96AG4	Leucine-rich repeat-containing protein 59	LRRC59	23.8	0.7820
P47914	60S ribosomal protein L29	RPL29	10.1	0.7831
P43243	Matrin-3	MATR3	9.8	0.7839
P52815	39S ribosomal protein L12, mitochondrial	MRPL12	23.2	0.7847
P05023;P50993;P13637;P54707;P20648;Q13733	Sodium/potassium-transporting ATPase subunit alpha-1	ATP1A1	9.3	0.7850
P12956	X-ray repair cross-complementing protein 6	XRCC6	21	0.7868
Q8IVL6	Prolyl 3-hydroxylase 3	LEPREL2	2.7	0.7892
Q8NBS9	Thioredoxin domain-containing protein 5	TXNDC5	13.2	0.7897
P13010	X-ray repair cross-complementing protein 5	XRCC5	18.4	0.7903
P10644	cAMP-dependent protein kinase type I-alpha regulatory subunit	PRKAR1A	8.9	0.7908
Q13838	Spliceosome RNA helicase DDX39B	DDX39B	22.4	0.7908
Q14103	Heterogeneous nuclear ribonucleoprotein D0	HNRNPD	18.6	0.7918
P84090	Enhancer of rudimentary homolog	ERH	37.5	0.7920
P63279	SUMO-conjugating enzyme UBC9	UBE2I	19	0.7924
P84103	Serine/arginine-rich splicing factor 3	SRSF3	12.2	0.7935
P63244	Guanine nucleotide-binding protein subunit beta-2-like 1	GNB2L1	37.9	0.7938
P63173	60S ribosomal protein L38	RPL38	24.3	0.7939
Q13263	Transcription intermediary factor 1-beta	TRIM28	11.1	0.7966
O75718	Cartilage-associated protein	CRTAP	10	0.7968

P52272	Heterogeneous nuclear ribonucleoprotein M	HNRNPM	18.1	0.7969
P61604	10 kDa heat shock protein, mitochondrial	HSPE1	31.4	0.7971
Q15233	Non-POU domain-containing octamer-binding protein	NONO	8.7	0.7972
Q9BUJ2	Heterogeneous nuclear ribonucleoprotein U-like protein 1	HNRNPU L1	3.5	0.7976
P36578	60S ribosomal protein L4	RPL4	12.4	0.7981
Q15717	ELAV-like protein 1	ELAVL1	19.3	0.7992
Q63ZY3	KN motif and ankyrin repeat domain-containing protein 2	KANK2	4.5	0.8010
P38159;O75526;Q96E39	RNA-binding motif protein, X chromosome;RNA-binding motif protein, X chromosome, N-terminally processed	RBMX	7.4	0.8010
Q969H8	UPF0556 protein C19orf10	C19orf10	16.8	0.8034
P26373	60S ribosomal protein L13	RPL13	13.3	0.8035
Q14696	LDLR chaperone MESD	MESDC2	8.5	0.8038
P02545	Prelamin-A/C;Lamin-A/C	LMNA	23.5	0.8057
P61978	Heterogeneous nuclear ribonucleoprotein K	HNRNPK	28.3	0.8066
Q13765;Q9BZK3	Nascent polypeptide-associated complex subunit alpha	NACA	26.5	0.8068
P83731	60S ribosomal protein L24	RPL24	17.8	0.8071
P02786	Transferrin receptor protein 1;Transferrin receptor protein 1, serum form	TFRC	18.2	0.8074
P42677	40S ribosomal protein S27	RPS27	25	0.8085
P60033	CD81 antigen	CD81	25	0.8092
Q15366;P57721	Poly(rC)-binding protein 2;Poly(rC)-binding protein 3	PCBP2;PCBP3	8.2	0.8113
O94855	Protein transport protein Sec24D	SEC24D	2.3	0.8118
P62979;P0CG48;P0CG47;P62987	Ubiquitin-40S ribosomal protein S27a;Ubiquitin;40S ribosomal protein S27a;Polyubiquitin-C;Ubiquitin;Polyubiquitin-B;Ubiquitin;Ubiquitin-60S ribosomal protein L40;Ubiquitin;60S ribosomal protein L40	RPS27A;UBC;UBB;UBA52	24.4	0.8123
P05386	60S acidic ribosomal protein P1	RPLP1	60.5	0.8141
P08865	40S ribosomal protein SA	RPSA	20.3	0.8144
P00568	Adenylate kinase isoenzyme 1	AK1	30.4	0.8152
Q01844	RNA-binding protein EWS	EWSR1	3.5	0.8165
O75534	Cold shock domain-containing protein E1	CSDE1	2.8	0.8173

P36405	ADP-ribosylation factor-like protein 3	ARL3	12.6	0.8181
P25398	40S ribosomal protein S12	RPS12	48.5	0.8185
P11940;Q9H361;Q4VXU2;Q96DU9	Polyadenylate-binding protein 1;Polyadenylate-binding protein 3	PABPC1; PABPC3	17.8	0.8195
Q14152	Eukaryotic translation initiation factor 3 subunit A	EIF3A	7.2	0.8195
P47813;O14602	Eukaryotic translation initiation factor 1A, X-chromosomal;Eukaryotic translation initiation factor 1A, Y-chromosomal	EIF1AX; EIF1AY	18.1	0.8224
Q9UBE0	SUMO-activating enzyme subunit 1	SAE1	10.1	0.8224
Q9Y5S9	RNA-binding protein 8A	RBM8A	21.3	0.8224
P62269	40S ribosomal protein S18	RPS18	18.4	0.8228
Q01082;Q9H254;O15020; P11277	Spectrin beta chain, brain 1	SPTBN1	8	0.8228
O75955	Flotillin-1	FLOT1	8.7	0.8239
P62701;P22090;Q8TD47	40S ribosomal protein S4, X isoform;40S ribosomal protein S4, Y isoform 1	RPS4X;RPS4Y1	19.8	0.8239
P20337	Ras-related protein Rab-3B	RAB3B	15.5	0.8244
P62847	40S ribosomal protein S24	RPS24	25.6	0.8248
P05455	Lupus La protein	SSB	27.7	0.8248
P46109	Crk-like protein	CRKL	9.6	0.8249
P67809;P16989;Q9Y2T7	Nuclease-sensitive element-binding protein 1	YBX1	48.8	0.8280
P26599;O95758	Polypyrimidine tract-binding protein 1	PTBP1	20.5	0.8281
Q92973	Transportin-1	TNPO1	5	0.8283
P63220	40S ribosomal protein S21	RPS21	30.1	0.8290
P62328	Thymosin beta-4;Hematopoietic system regulatory peptide	TMSB4X	31.8	0.8298
Q12905	Interleukin enhancer-binding factor 2	ILF2	19	0.8301
P62854;Q5JNZ5	40S ribosomal protein S26;Putative 40S ribosomal protein S26-like 1	RPS26;RPS26P11	23.5	0.8306
P55209	Nucleosome assembly protein 1-like 1	NAP1L1	10.2	0.8308
P22695	Cytochrome b-c1 complex subunit 2, mitochondrial	UQCRC2	10.4	0.8319
Q15185	Prostaglandin E synthase 3	PTGES3	31.2	0.8344
P62851	40S ribosomal protein S25	RPS25	21.6	0.8356

P51114;P51116;Q06787	Fragile X mental retardation syndrome-related protein 1;Fragile X mental retardation syndrome-related protein 2;Fragile X mental retardation protein 1	FXR1;FXR2;FMR1	4.7	0.8362
P39023	60S ribosomal protein L3	RPL3	26.3	0.8362
P56192	Methionine--tRNA ligase, cytoplasmic	MARS	3.9	0.8363
Q08211	ATP-dependent RNA helicase A	DHX9	13	0.8364
P62424	60S ribosomal protein L7a	RPL7A	27.8	0.8368
P40227;Q92526	T-complex protein 1 subunit zeta	CCT6A	42.2	0.8386
P49591	Serine--tRNA ligase, cytoplasmic	SARS	13	0.8386
P80723	Brain acid soluble protein 1	BASP1	51.1	0.8405
P22626	Heterogeneous nuclear ribonucleoproteins A2/B1	HNRNPA2B1	13.9	0.8412
P62633	Cellular nucleic acid-binding protein	CNBP	11.3	0.8413
P41567;O60739	Eukaryotic translation initiation factor 1;Eukaryotic translation initiation factor 1b	EIF1;EIF1B	23.9	0.8415
P62910	60S ribosomal protein L32	RPL32	22.2	0.8425
Q01813	6-phosphofructokinase type C	PFKP	11.5	0.8434
O43390	Heterogeneous nuclear ribonucleoprotein R	HNRNPR	19.6	0.8435
O00303	Eukaryotic translation initiation factor 3 subunit F	EIF3F	14.8	0.8463
P26639	Threonine--tRNA ligase, cytoplasmic	TARS	9.4	0.8466
Q13283	Ras GTPase-activating protein-binding protein 1	G3BP1	18.7	0.8466
P78371	T-complex protein 1 subunit beta	CCT2	31	0.8471
P62888	60S ribosomal protein L30	RPL30	26.1	0.8482
Q02878	60S ribosomal protein L6	RPL6	19.1	0.8484
P04844	Dolichyl-diphosphooligosaccharide--protein glycosyltransferase subunit 2	RPN2	14.4	0.8494
P55145	Mesencephalic astrocyte-derived neurotrophic factor	MANF	23.6	0.8501
Q9UJZ1	Stomatin-like protein 2	STOML2	12.9	0.8510
P30040	Endoplasmic reticulum resident protein 29	ERP29	23.4	0.8511
P46060	Ran GTPase-activating protein 1	RANGAP1	23.5	0.8511
P50991	T-complex protein 1 subunit delta	CCT4	34.9	0.8521
P26368	Splicing factor U2AF 65 kDa subunit	U2AF2	12.8	0.8524
Q16658	Fascin	FSCN1	18.5	0.8533

O00571;O15523	ATP-dependent RNA helicase DDX3X;ATP-dependent RNA helicase DDX3Y	DDX3X; DDX3Y	10	0.8544
P15531	Nucleoside diphosphate kinase A	NME1	15.8	0.8544
P43307	Translocon-associated protein subunit alpha	SSR1	6.6	0.8547
P49207	60S ribosomal protein L34	RPL34	13.7	0.8548
Q9Y224	UPF0568 protein C14orf166	C14orf166	20.9	0.8562
P20042	Eukaryotic translation initiation factor 2 subunit 2	EIF2S2	11.4	0.8565
Q9UQ80	Proliferation-associated protein 2G4	PA2G4	31.7	0.8568
Q15046	Lysine--tRNA ligase	KARS	3.5	0.8586
P31948	Stress-induced-phosphoprotein 1	STIP1	25.4	0.8589
P55735	Protein SEC13 homolog	SEC13	9.9	0.8595
P04181	Ornithine aminotransferase, mitochondrial;Ornithine aminotransferase, hepatic form;Ornithine aminotransferase, renal form	OAT	13.9	0.8597
Q15417	Calponin-3	CNN3	25.5	0.8606
P51858	Hepatoma-derived growth factor	HDGF	22.1	0.8610
P61254;Q9UNX3	60S ribosomal protein L26;60S ribosomal protein L26-like 1	RPL26;RPL26L1	11	0.8611
P35613	Basigin	BSG	7.8	0.8613
Q14974	Importin subunit beta-1	KPNB1	9.8	0.8633
P61247	40S ribosomal protein S3a	RPS3A	26.5	0.8641
P49368	T-complex protein 1 subunit gamma	CCT3	19.3	0.8645
Q99832	T-complex protein 1 subunit eta	CCT7	30.9	0.8651
Q92900	Regulator of nonsense transcripts 1	UPF1	2.7	0.8657
P25685;Q9UDY4	DnaJ homolog subfamily B member 1	DNAJB1	11.5	0.8664
Q02543	60S ribosomal protein L18a	RPL18A	18.2	0.8664
P39656	Dolichyl-diphosphooligosaccharide--protein glycosyltransferase 48 kDa subunit	DDOST	15.4	0.8665
Q92841	Probable ATP-dependent RNA helicase DDX17	DDX17	6.7	0.8687
P46781	40S ribosomal protein S9	RPS9	18.6	0.8688
O75531	Barrier-to-autointegration factor	BANF1	29.2	0.8690
P61916	Epididymal secretory protein E1	NPC2	50.3	0.8699
Q13443	Disintegrin and metalloproteinase domain-containing protein 9	ADAM9	3.2	0.8704
Q99439	Calponin-2	CNN2	27.5	0.8705

Q8NC51	Plasminogen activator inhibitor 1 RNA-binding protein	SERBP1	12.3	0.8709
P68104;Q5VTE0;Q05639	Elongation factor 1-alpha 1;Putative elongation factor 1-alpha-like 3;Elongation factor 1-alpha 2	EEF1A1; EEF1A1P5;EEF1A2	31.8	0.8715
P62906	60S ribosomal protein L10a	RPL10A	23	0.8718
P12236;P12235	ADP/ATP translocase 3	SLC25A6	31.9	0.8722
P46777	60S ribosomal protein L5	RPL5	33.3	0.8723
Q58FF8	Putative heat shock protein HSP 90-beta 2	HSP90AB2P	13.9	0.8723
P55036;A2A3N6	26S proteasome non-ATPase regulatory subunit 4	PSMD4	14.1	0.8730
Q13907	Isopentenyl-diphosphate Delta-isomerase 1	IDI1	9.3	0.8731
P49419	Alpha-aminoadipic semialdehyde dehydrogenase	ALDH7A1	7.6	0.8732
P62750	60S ribosomal protein L23a	RPL23A	35.9	0.8735
P56537	Eukaryotic translation initiation factor 6	EIF6	9.8	0.8738
P67936	Tropomyosin alpha-4 chain	TPM4	32.3	0.8744
P62753	40S ribosomal protein S6	RPS6	22.9	0.8754
P61513;A6NKH3	60S ribosomal protein L37a	RPL37A	41.3	0.8760
Q02952	A-kinase anchor protein 12	AKAP12	5.9	0.8763
Q9UN86	Ras GTPase-activating protein-binding protein 2	G3BP2	10.4	0.8767
Q7L2H7	Eukaryotic translation initiation factor 3 subunit M	EIF3M	15.5	0.8767
P00492	Hypoxanthine-guanine phosphoribosyltransferase	HPRT1	16.5	0.8772
P05387	60S acidic ribosomal protein P2	RPLP2	55.7	0.8772
Q07020	60S ribosomal protein L18	RPL18	18.6	0.8783
Q9UNM6	26S proteasome non-ATPase regulatory subunit 13	PSMD13	13.6	0.8787
Q96AY3	Peptidyl-prolyl cis-trans isomerase FKBP10	FKBP10	12.2	0.8789
P18621	60S ribosomal protein L17	RPL17	19.6	0.8800
P61313	60S ribosomal protein L15	RPL15	12.7	0.8801
P15880	40S ribosomal protein S2	RPS2	42	0.8813
P46779	60S ribosomal protein L28	RPL28	21.2	0.8820
P35221	Catenin alpha-1	CTNNA1	14.2	0.8825
P04899	Guanine nucleotide-binding protein G(i) subunit alpha-2	GNAI2	27	0.8852

P48960	CD97 antigen;CD97 antigen subunit alpha;CD97 antigen subunit beta	CD97	4	0.8856
P62913	60S ribosomal protein L11	RPL11	16.9	0.8857
P62081	40S ribosomal protein S7	RPS7	41.8	0.8864
P19525	Interferon-induced, double-stranded RNA-activated protein kinase	EIF2AK2	4.7	0.8868
Q14444	Caprin-1	CAPRIN1	13.8	0.8875
P41091;Q2VIR3	Eukaryotic translation initiation factor 2 subunit 3;Putative eukaryotic translation initiation factor 2 subunit 3-like protein	EIF2S3;EIF2S3L	14.6	0.8879
P17987	T-complex protein 1 subunit alpha	TCP1	35.6	0.8890
O43237	Cytoplasmic dynein 1 light intermediate chain 2	DYNC1LI2	6.5	0.8893
P11142	Heat shock cognate 71 kDa protein	HSPA8	34.2	0.8893
P42704	Leucine-rich PPR motif-containing protein, mitochondrial	LRPPRC	13.1	0.8901
P29692	Elongation factor 1-delta	EEF1D	21	0.8909
P30050	60S ribosomal protein L12	RPL12	24.2	0.8909
O60506	Heterogeneous nuclear ribonucleoprotein Q	SYNCRIP	15.2	0.8914
P04843	Dolichyl-diphosphooligosaccharide--protein glycosyltransferase subunit 1	RPN1	21.3	0.8914
Q14697	Neutral alpha-glucosidase AB	GANAB	10.3	0.8916
P52292	Importin subunit alpha-2	KPNA2	8.1	0.8916
P63241;Q9GZV4;Q6IS14	Eukaryotic translation initiation factor 5A-1;Eukaryotic translation initiation factor 5A-2;Eukaryotic translation initiation factor 5A-1-like	EIF5A;EIF5A2;EIF5AL1	29.2	0.8921
P24534	Elongation factor 1-beta	EEF1B2	20	0.8938
Q96QD8	Sodium-coupled neutral amino acid transporter 2	SLC38A2	7.3	0.8939
P08238;Q58FF7	Heat shock protein HSP 90-beta	HSP90AB1	15.3	0.8940
Q9BXJ9	N-alpha-acetyltransferase 15, NatA auxiliary subunit	NAA15	1.8	0.8944
Q13813	Spectrin alpha chain, brain	SPTAN1	7	0.8946
Q3ZCM7	Tubulin beta-8 chain	TUBB8	9.2	0.8953
P61019;Q8WUD1	Ras-related protein Rab-2A;Ras-related protein Rab-2B	RAB2A;RAB2B	26.4	0.8961
Q16186	Proteasomal ubiquitin receptor ADRM1	ADRM1	8.1	0.8962

P62249	40S ribosomal protein S16	RPS16	12.3	0.8964
O14980	Exportin-1	XPO1	3.7	0.8969
P13693;Q56UQ5	Translationally-controlled tumor protein;TPT1-like protein	TPT1	23.8	0.8977
O75821	Eukaryotic translation initiation factor 3 subunit G	EIF3G	18.1	0.8986
P27695	DNA-(apurinic or apyrimidinic site) lyase;DNA-(apurinic or apyrimidinic site) lyase, mitochondrial	APEX1	12.3	0.8999
Q15365	Poly(rC)-binding protein 1	PCBP1	32.3	0.9002
Q9Y512	Sorting and assembly machinery component 50 homolog	SAMM50	3.2	0.9009
P50990	T-complex protein 1 subunit theta	CCT8	35.6	0.9019
P26196	Probable ATP-dependent RNA helicase DDX6	DDX6	7.5	0.9019
O76003	Glutaredoxin-3	GLRX3	14.3	0.9024
O95373;O15397	Importin-7	IPO7	6.2	0.9037
Q16698	2,4-dienoyl-CoA reductase, mitochondrial	DECR1	7.8	0.9040
Q70UQ0	Inhibitor of nuclear factor kappa-B kinase-interacting protein	IKBIP	9.4	0.9059
P18124	60S ribosomal protein L7	RPL7	27.8	0.9069
Q13310;P0CB38	Polyadenylate-binding protein 4	PABPC4	11.2	0.9074
P07900;Q58FG0;Q14568	Heat shock protein HSP 90-alpha	HSP90AA1	27	0.9079
P46778	60S ribosomal protein L21	RPL21	25	0.9081
Q92896	Golgi apparatus protein 1	GLG1	2.7	0.9081
O43615	Mitochondrial import inner membrane translocase subunit TIM44	TIMM44	4.9	0.9091
P13639	Elongation factor 2	EEF2	38.8	0.9092
Q9H488	GDP-fucose protein O-fucosyltransferase 1	POFUT1	6.7	0.9103
Q99613	Eukaryotic translation initiation factor 3 subunit C	EIF3C	3.9	0.9109
P23528	Cofilin-1	CFL1	68.1	0.9114
P08107;P48741	Heat shock 70 kDa protein 1A/1B	HSPA1A	28.4	0.9116
P48643	T-complex protein 1 subunit epsilon	CCT5	27	0.9119
Q9BT78	COP9 signalosome complex subunit 4	COPS4	12.6	0.9128
P05388;Q8N	60S acidic ribosomal protein P0;60S acidic	RPLP0;R	45.4	0.9132

HW5	ribosomal protein P0-like	PLP0P6		
Q9Y490	Talin-1	TLN1	24.4	0.9133
P54577	Tyrosine--tRNA ligase, cytoplasmic	YARS	10.8	0.9135
Q12906;Q96SI9	Interleukin enhancer-binding factor 3	ILF3	5.9	0.9137
P60228	Eukaryotic translation initiation factor 3 subunit E	EIF3E	13.7	0.9147
Q7Z2W4	Zinc finger CCCH-type antiviral protein 1	ZC3HAV1	3.5	0.9155
P08708;P0CW22	40S ribosomal protein S17;40S ribosomal protein S17-like	RPS17;RPS17L	39.3	0.9157
Q99873	Protein arginine N-methyltransferase 1	PRMT1	9.1	0.9163
P31930	Cytochrome b-c1 complex subunit 1, mitochondrial	UQCRC1	9.2	0.9163
P07437	Tubulin beta chain	TUBB	20.5	0.9170
P14314	Glucosidase 2 subunit beta	PRKCSH	26.3	0.9179
O75822	Eukaryotic translation initiation factor 3 subunit J	EIF3J	13.6	0.9204
P62195	26S protease regulatory subunit 8	PSMC5	13.8	0.9206
Q9H1E3	Nuclear ubiquitous casein and cyclin-dependent kinase substrate 1	NUCKS1	10.7	0.9221
O00232	26S proteasome non-ATPase regulatory subunit 12	PSMD12	5.5	0.9228
P17980	26S protease regulatory subunit 6A	PSMC3	21.4	0.9245
P25788	Proteasome subunit alpha type-3	PSMA3	11	0.9256
P62841	40S ribosomal protein S15	RPS15	40	0.9257
P46821	Microtubule-associated protein 1B;MAP1 light chain LC1	MAP1B	6	0.9276
P22102	Trifunctional purine biosynthetic protein adenosine-3;Phosphoribosylamine--glycine ligase;Phosphoribosylformylglycinamidine cyclo-ligase;Phosphoribosylglycinamide formyltransferase	GART	6.5	0.9280
Q99471	Prefoldin subunit 5	PFDN5	42.9	0.9281
Q15293	Reticulocalbin-1	RCN1	7.3	0.9282
P32969	60S ribosomal protein L9	RPL9	45.8	0.9291
P35998	26S protease regulatory subunit 7	PSMC2	11.8	0.9291
P62314	Small nuclear ribonucleoprotein Sm D1	SNRPD1	27.7	0.9291
P17812	CTP synthase 1	CTPS	5.1	0.9294
Q9Y262	Eukaryotic translation initiation factor 3 subunit L	EIF3L	8.3	0.9295
Q9UHD8	Septin-9	Sep-09	9.9	0.9302

P56199	Integrin alpha-1	ITGA1	6.3	0.9311
P49327	Fatty acid synthase;[Acyl-carrier-protein] S-acetyltransferase;[Acyl-carrier-protein] S-malonyltransferase;3-oxoacyl-[acyl-carrier-protein] synthase;3-oxoacyl-[acyl-carrier-protein] reductase;3-hydroxypalmitoyl-[acyl-carrier-protein] dehydratase;Enoyl-[acyl-carrier-protein] reductase;Oleoyl-[acyl-carrier-protein] hydrolase	FASN	7.2	0.9318
O14579	Coatomer subunit epsilon	COPE	26	0.9325
Q9Y266	Nuclear migration protein nudC	NUDC	19	0.9354
Q7L1Q6;Q9Y6E2	Basic leucine zipper and W2 domain-containing protein 1	BZW1	5.5	0.9356
O00629	Importin subunit alpha-4	KPNA4	13.8	0.9357
P54578	Ubiquitin carboxyl-terminal hydrolase 14	USP14	4.5	0.9372
P78527	DNA-dependent protein kinase catalytic subunit	PRKDC	2.1	0.9375
Q04917	14-3-3 protein eta	YWHAH	33.3	0.9376
P48059;Q7Z4I7	LIM and senescent cell antigen-like-containing domain protein 1	LIMS1	20.9	0.9381
Q8IWE2	Protein NOXP20	FAM114A1	6.2	0.9384
Q9UBQ5	Eukaryotic translation initiation factor 3 subunit K	EIF3K	12.8	0.9393
O00410;O60518	Importin-5	IPO5	20	0.9399
P62826	GTP-binding nuclear protein Ran	RAN	33.3	0.9402
P17096	High mobility group protein HMG-I/HMG-Y	HMGA1	23.4	0.9407
P25787	Proteasome subunit alpha type-2	PSMA2	19.7	0.9415
Q32MZ4	Leucine-rich repeat flightless-interacting protein 1	LRRFIP1	9.7	0.9422
P46783	40S ribosomal protein S10	RPS10	6.7	0.9425
Q9Y281	Cofilin-2	CFL2	48.8	0.9428
P63261	Actin, cytoplasmic 2;Actin, cytoplasmic 2, N-terminally processed	ACTG1	54.4	0.9433
P50502;Q8NFI4;Q8IZP2	Hsc70-interacting protein;Putative protein FAM10A5;Putative protein FAM10A4	ST13;ST13P5;ST13P4	13.3	0.9451
O14818;Q8TAA3	Proteasome subunit alpha type-7;Proteasome subunit alpha type-7-like	PSMA7;PSMA8	19.4	0.9452
P48444	Coatomer subunit delta	ARCN1	14.3	0.9464
Q14019	Coactosin-like protein	COTL1	19.7	0.9464

P61769	Beta-2-microglobulin;Beta-2-microglobulin form pI 5.3	B2M	35.3	0.9470
P04406	Glyceraldehyde-3-phosphate dehydrogenase	GAPDH	50.1	0.9487
P00505	Aspartate aminotransferase, mitochondrial	GOT2	19.1	0.9501
Q02818	Nucleobindin-1	NUCB1	4.6	0.9504
P62136;P62140;P36873	Serine/threonine-protein phosphatase PP1-alpha catalytic subunit;Serine/threonine-protein phosphatase PP1-beta catalytic subunit;Serine/threonine-protein phosphatase PP1-gamma catalytic subunit	PPP1CA; PPP1CB; PPP1CC	27.3	0.9505
P28066	Proteasome subunit alpha type-5	PSMA5	34.4	0.9513
P43686	26S protease regulatory subunit 6B	PSMC4	20.8	0.9526
Q92598	Heat shock protein 105 kDa	HSPH1	28.1	0.9537
P61960	Ubiquitin-fold modifier 1	UFM1	58.8	0.9542
Q9Y2Z0	Suppressor of G2 allele of SKP1 homolog	SUGT1	11.8	0.9565
P26885	Peptidyl-prolyl cis-trans isomerase FKBP2	FKBP2	28.9	0.9568
P16949;Q93045	Stathmin	STMN1	22.1	0.9568
P57088	Transmembrane protein 33	TMEM33	13	0.9570
Q07866;Q9H0B6;Q9NSK0	Kinesin light chain 1;Kinesin light chain 2;Kinesin light chain 4	KLC1;KLC2;KLC4	9.1	0.9576
O00159	Unconventional myosin-Ic	MYO1C	5.8	0.9581
P62714;P67775	Serine/threonine-protein phosphatase 2A catalytic subunit beta isoform;Serine/threonine-protein phosphatase 2A catalytic subunit alpha isoform	PPP2CB; PPP2CA	9.1	0.9604
Q9Y3F4	Serine-threonine kinase receptor-associated protein	STRAP	36.3	0.9608
Q09666	Neuroblast differentiation-associated protein AHNAK	AHNAK	46.6	0.9611
P23396	40S ribosomal protein S3	RPS3	28	0.9614
Q7KZF4	Staphylococcal nuclease domain-containing protein 1	SND1	18.2	0.9615
Q08623	Pseudouridine-5-monophosphatase	HDHD1	7.9	0.9619
P62258	14-3-3 protein epsilon	YWHAE	31.8	0.9630
Q14157	Ubiquitin-associated protein 2-like	UBAP2L	10.6	0.9631
P62829	60S ribosomal protein L23	RPL23	25.7	0.9650
P25789	Proteasome subunit alpha type-4	PSMA4	28.7	0.9653
P06756	Integrin alpha-V;Integrin alpha-V heavy chain;Integrin alpha-V light chain	ITGAV	9	0.9654

Q9Y3I0	tRNA-splicing ligase RtcB homolog	C22orf28	23.6	0.9657
P68036	Ubiquitin-conjugating enzyme E2 L3	UBE2L3	17.5	0.9685
Q9UKY7	Protein CDV3 homolog	CDV3	21.3	0.9708
P62879	Guanine nucleotide-binding protein G(I)/G(S)/G(T) subunit beta-2	GNB2	21.8	0.9739
P26641	Elongation factor 1-gamma	EEF1G	21.1	0.9753
Q99460	26S proteasome non-ATPase regulatory subunit 1	PSMD1	15.4	0.9756
O60701	UDP-glucose 6-dehydrogenase	UGDH	19.8	0.9757
P13073	Cytochrome c oxidase subunit 4 isoform 1, mitochondrial	COX4I1	13	0.9763
Q15435	Protein phosphatase 1 regulatory subunit 7	PPP1R7	10.3	0.9768
P62241	40S ribosomal protein S8	RPS8	10.6	0.9778
P49720	Proteasome subunit beta type-3	PSMB3	26.3	0.9781
Q9NR12	PDZ and LIM domain protein 7	PDLIM7	19	0.9787
Q9BQE3	Tubulin alpha-1C chain	TUBA1C	35.4	0.9794
P49411	Elongation factor Tu, mitochondrial	TUFM	26.3	0.9797
P07737;CON__P02584	Profilin-1	PFN1	71.4	0.9809
O00231	26S proteasome non-ATPase regulatory subunit 11	PSMD11	11.8	0.9815
P07814	Bifunctional glutamate/proline--tRNA ligase;Glutamate--tRNA ligase;Proline--tRNA ligase	EPRS	3.7	0.9830
Q92499	ATP-dependent RNA helicase DDX1	DDX1	10.7	0.9837
Q99714	3-hydroxyacyl-CoA dehydrogenase type-2	HSD17B10	16.5	0.9851
P23284	Peptidyl-prolyl cis-trans isomerase B	PPIB	38	0.9853
Q92905	COP9 signalosome complex subunit 5	COPS5	6.9	0.9864
P15170;Q8IYD1	Eukaryotic peptide chain release factor GTP-binding subunit ERF3A	GSPT1	13	0.9867
O95793	Double-stranded RNA-binding protein Staufen homolog 1	STAU1	10.4	0.9872
Q9Y696	Chloride intracellular channel protein 4	CLIC4	46.2	0.9874
P40222	Alpha-taxilin	TXLNA	3.3	0.9876
Q13418	Integrin-linked protein kinase	ILK	12.2	0.9878
P28331	NADH-ubiquinone oxidoreductase 75 kDa subunit, mitochondrial	NDUFS1	12.1	0.9883
P13797;Q146	Plastin-3	PLS3	29.5	0.9906

51;P13796				
P07954	Fumarate hydratase, mitochondrial	FH	26.7	0.9911
P51149	Ras-related protein Rab-7a	RAB7A	31.4	0.9911
P80303	Nucleobindin-2	NUCB2	9.3	0.9915
Q16543	Hsp90 co-chaperone Cdc37	CDC37	13.8	0.9917
P08621	U1 small nuclear ribonucleoprotein 70 kDa	SNRNP70	5.7	0.9918
O00299	Chloride intracellular channel protein 1	CLIC1	41.5	0.9918
P37837	Transaldolase	TALDO1	31.8	0.9921
Q15181	Inorganic pyrophosphatase	PPA1	27.7	0.9929
Q562R1	Beta-actin-like protein 2	ACTBL2	14.1	0.9930
P61586	Transforming protein RhoA	RHOA	24.9	0.9933
P21333	Filamin-A	FLNA	33.4	0.9934
Q9Y277	Voltage-dependent anion-selective channel protein 3	VDAC3	27.9	0.9943
Q96JJ7	Protein disulfide-isomerase TMX3	TMX3	5.3	0.9947
P30153;P30154	Serine/threonine-protein phosphatase 2A 65 kDa regulatory subunit A alpha isoform	PPP2R1A	22.2	0.9949
Q9Y678;Q9UBF2	Coatomer subunit gamma-1	COPG1	23.1	0.9951
Q9P2E9	Ribosome-binding protein 1	RRBP1	14.6	0.9978
P54136	Arginine--tRNA ligase, cytoplasmic	RARS	9.5	0.9982
O43396	Thioredoxin-like protein 1	TXNL1	20.4	0.9984
O94979	Protein transport protein Sec31A	SEC31A	10.7	0.9984
Q15691;Q9UPY8	Microtubule-associated protein RP/EB family member 1	MAPRE1	22.8	0.9985
P28838	Cytosol aminopeptidase	LAP3	17.1	0.9998
Q96FW1	Ubiquitin thioesterase OTUB1	OTUB1	14.4	1.0000
P22314	Ubiquitin-like modifier-activating enzyme 1	UBA1	19.9	1.0019
Q01995	Transgelin	TAGLN	51.7	1.0023
P22061	Protein-L-isoaspartate(D-aspartate) O-methyltransferase	PCMT1	15	1.0025
P62873;Q9HAV0;P16520	Guanine nucleotide-binding protein G(I)/G(S)/G(T) subunit beta-1	GNB1	28.8	1.0033
P06730;A6NMX2	Eukaryotic translation initiation factor 4E;Eukaryotic translation initiation factor 4E type 1B	EIF4E;EIF4E1B	8.3	1.0050
Q15404	Ras suppressor protein 1	RSU1	20.2	1.0057

Q13200	26S proteasome non-ATPase regulatory subunit 2	PSMD2	12.7	1.0058
Q92734	Protein TFG	TFG	6.8	1.0059
P06733;P13929	Alpha-enolase	ENO1	47.7	1.0062
Q9NVD7;Q9HBI1	Alpha-parvin	PARVA	9.7	1.0063
Q9H4M9	EH domain-containing protein 1	EHD1	7.3	1.0065
P22234	Multifunctional protein ADE2;Phosphoribosylaminoimidazole-succinocarboxamide synthase;Phosphoribosylaminoimidazole carboxylase	PAICS	14.4	1.0065
Q8WX93;Q86TC9	Palladin	PALLD	14.2	1.0066
P06753	Tropomyosin alpha-3 chain	TPM3	12.7	1.0076
Q9Y265	RuvB-like 1	RUVBL1	14.3	1.0089
O94905	Erlin-2	ERLIN2	10	1.0103
P20020;Q16720;Q01814	Plasma membrane calcium-transporting ATPase 1;Plasma membrane calcium-transporting ATPase 3	ATP2B1; ATP2B3	8.3	1.0103
P16615;O14983;Q93084	Sarcoplasmic/endoplasmic reticulum calcium ATPase 2	ATP2A2	8	1.0110
P36957	Dihydrolipoyllysine-residue succinyltransferase component of 2-oxoglutarate dehydrogenase complex, mitochondrial	DLST	6.4	1.0145
P62333	26S protease regulatory subunit 10B	PSMC6	6.7	1.0145
Q6NUK1	Calcium-binding mitochondrial carrier protein SCaMC-1	SLC25A24	4.2	1.0161
Q16270	Insulin-like growth factor-binding protein 7	IGFBP7	18.4	1.0172
Q13155	Aminoacyl tRNA synthase complex-interacting multifunctional protein 2	AIMP2	6.9	1.0173
P35232	Prohibitin	PHB	11.4	1.0179
Q6DD88	Atlastin-3	ATL3	25.9	1.0182
P05556	Integrin beta-1	ITGB1	22.9	1.0187
Q8WUM4	Programmed cell death 6-interacting protein	PDCD6IP	14.1	1.0193
Q9UL46	Proteasome activator complex subunit 2	PSME2	25.1	1.0194
Q9Y3B3	Transmembrane emp24 domain-containing protein 7	TMED7	16.1	1.0198
Q9UMX0	Ubiquilin-1	UBQLN1	6.8	1.0201

P41252	Isoleucine--tRNA ligase, cytoplasmic	IARS	5.4	1.0223
Q96AC1	Fermitin family homolog 2	FERMT2	12.1	1.0225
Q9NP72	Ras-related protein Rab-18	RAB18	17.5	1.0229
O60763	General vesicular transport factor p115	USO1	7.4	1.0234
P07355;A6NMY6	Annexin A2;Putative annexin A2-like protein	ANXA2; ANXA2P2	43.1	1.0248
Q96G03	Phosphoglucomutase-2	PGM2	10	1.0258
Q9Y2X3	Nucleolar protein 58	NOP58	6.8	1.0258
P60983;O60234	Glia maturation factor beta;Glia maturation factor gamma	GMFB;GMFG	17.6	1.0259
Q9Y4F1	FERM, RhoGEF and pleckstrin domain-containing protein 1	FARP1	3	1.0270
Q9UJ70	N-acetyl-D-glucosamine kinase	NAGK	13.1	1.0282
P50395	Rab GDP dissociation inhibitor beta	GDI2	42	1.0290
P0C7P4;P47985	Putative cytochrome b-c1 complex subunit Rieske-like protein 1;Cytochrome b-c1 complex subunit Rieske, mitochondrial;Cytochrome b-c1 complex subunit 11	UQCRFS1P1;UQCRFS1	7.4	1.0310
Q9NZN4	EH domain-containing protein 2	EHD2	16.4	1.0315
P35606	Coatomer subunit beta	COPB2	11.7	1.0316
P68371	Tubulin beta-4B chain	TUBB4B	20.4	1.0319
P51148	Ras-related protein Rab-5C	RAB5C	18.1	1.0321
O60493;Q9UMY4	Sorting nexin-3	SNX3	15.4	1.0321
Q04637;O43432	Eukaryotic translation initiation factor 4 gamma 1	EIF4G1	4.1	1.0356
P09622	Dihydrolipoyl dehydrogenase, mitochondrial	DLD	4.7	1.0366
O00487	26S proteasome non-ATPase regulatory subunit 14	PSMD14	17.7	1.0368
P24539	ATP synthase subunit b, mitochondrial	ATP5F1	7.8	1.0383
P60900	Proteasome subunit alpha type-6	PSMA6	25.6	1.0384
Q07065	Cytoskeleton-associated protein 4	CKAP4	22.8	1.0385
Q00325	Phosphate carrier protein, mitochondrial	SLC25A3	7.2	1.0389
P27348	14-3-3 protein theta	YWHAQ	35.1	1.0400
Q02809	Procollagen-lysine,2-oxoglutarate 5-dioxygenase 1	PLOD1	10.5	1.0403
O43399	Tumor protein D54	TPD52L2	18.4	1.0430
P33176;O602	Kinesin-1 heavy chain	KIF5B	15.7	1.0440

82;Q12840				
P61981	14-3-3 protein gamma;14-3-3 protein gamma, N-terminally processed	YWHAG	32.4	1.0441
O75347	Tubulin-specific chaperone A	TBCA	27.8	1.0451
Q9H0U4;Q92928	Ras-related protein Rab-1B;Putative Ras-related protein Rab-1C	RAB1B;RAB1C	30.8	1.0454
Q9HDC9	Adipocyte plasma membrane-associated protein	APMAP	8.4	1.0459
P61923	Coatomer subunit zeta-1	COPZ1	13.6	1.0461
P63104	14-3-3 protein zeta/delta	YWHAZ	49	1.0476
Q16891	Mitochondrial inner membrane protein	IMMT	11.2	1.0489
P24752	Acetyl-CoA acetyltransferase, mitochondrial	ACAT1	21.1	1.0496
Q99733	Nucleosome assembly protein 1-like 4	NAP1L4	19.5	1.0496
P38646	Stress-70 protein, mitochondrial	HSPA9	21.2	1.0497
Q9HCN8	Stromal cell-derived factor 2-like protein 1	SDF2L1	14.9	1.0504
P62191	26S protease regulatory subunit 4	PSMC1	11.4	1.0523
Q9ULV4	Coronin-1C	CORO1C	18.4	1.0546
Q9Y6Y8	SEC23-interacting protein	SEC23IP	3.8	1.0550
Q99584	Protein S100-A13	S100A13	32.7	1.0555
P35237	Serpin B6	SERPINB6	32.2	1.0564
P61204;P84077	ADP-ribosylation factor 3;ADP-ribosylation factor 1	ARF3;ARF1	49.2	1.0565
Q96HC4	PDZ and LIM domain protein 5	PDLIM5	24.7	1.0570
P09382	Galectin-1	LGALS1	63	1.0572
P54727;P54725	UV excision repair protein RAD23 homolog B	RAD23B	9.3	1.0581
O75947	ATP synthase subunit d, mitochondrial	ATP5H	38.5	1.0582
Q13409	Cytoplasmic dynein 1 intermediate chain 2	DYNC1I2	8.3	1.0583
P40616	ADP-ribosylation factor-like protein 1	ARL1	13.8	1.0595
P21589	5-nucleotidase	NT5E	17.6	1.0606
O75506	Heat shock factor-binding protein 1	HSBP1	25	1.0609
P14618;P30613	Pyruvate kinase isozymes M1/M2	PKM2	46.3	1.0612
Q5JPE7;P69849;Q15155	Nodal modulator 2;Nodal modulator 3;Nodal modulator 1	NOMO2;NOMO3;NOMO1	2	1.0621
O94925	Glutaminase kidney isoform, mitochondrial	GLS	16.3	1.0625

Q12907	Vesicular integral-membrane protein VIP36	LMAN2	7.9	1.0626
P27797	Calreticulin	CALR	36	1.0636
P29401	Transketolase	TKT	26.5	1.0638
P30084	Enoyl-CoA hydratase, mitochondrial	ECHS1	20	1.0641
P16070	CD44 antigen	CD44	6.5	1.0644
Q01518	Adenylyl cyclase-associated protein 1	CAP1	45.7	1.0663
P47897	Glutamine--tRNA ligase	QARS	4.4	1.0670
P07305	Histone H1.0	H1F0	11.9	1.0671
O75390	Citrate synthase, mitochondrial	CS	9.7	1.0674
P16152	Carbonyl reductase [NADPH] 1	CBR1	26	1.0676
Q96KP4	Cytosolic non-specific dipeptidase	CNDP2	7.4	1.0688
P40926	Malate dehydrogenase, mitochondrial	MDH2	32.2	1.0690
P00367;P49448	Glutamate dehydrogenase 1, mitochondrial;Glutamate dehydrogenase 2, mitochondrial	GLUD1;GLUD2	15.9	1.0696
P41250	Glycine--tRNA ligase	GARS	14.6	1.0697
O75367	Core histone macro-H2A.1	H2AFY	11.3	1.0704
Q9H444	Charged multivesicular body protein 4b	CHMP4B	16.5	1.0704
Q15436	Protein transport protein Sec23A	SEC23A	8.4	1.0708
P62820	Ras-related protein Rab-1A	RAB1A	48.3	1.0732
P28070	Proteasome subunit beta type-4	PSMB4	16.7	1.0744
P25786	Proteasome subunit alpha type-1	PSMA1	18.3	1.0750
P53618	Coatomer subunit beta	COPB1	14.8	1.0758
P26038	Moesin	MSN	32.8	1.0764
Q12792;Q6IBS0	Twinfilin-1	TWF1	9.4	1.0767
Q13423	NAD(P) transhydrogenase, mitochondrial	NNT	5.7	1.0768
Q9UBT2	SUMO-activating enzyme subunit 2	UBA2	7	1.0791
Q93052	Lipoma-preferred partner	LPP	13.2	1.0792
O75083	WD repeat-containing protein 1	WDR1	44.9	1.0793
P12955	Xaa-Pro dipeptidase	PEPD	4.1	1.0797
P34932;O95757	Heat shock 70 kDa protein 4	HSPA4	20.1	1.0807
P48556	26S proteasome non-ATPase regulatory subunit 8	PSMD8	6.6	1.0815
P99999	Cytochrome c	CYCS	43.8	1.0829
P55884	Eukaryotic translation initiation factor 3 subunit B	EIF3B	5.4	1.0836
Q14956	Transmembrane glycoprotein NMB	GPNMB	3.1	1.0843

Q7L576;Q96F07	Cytoplasmic FMR1-interacting protein 1;Cytoplasmic FMR1-interacting protein 2	CYFIP1;CYFIP2	2.1	1.0845
P63000;P60763	Ras-related C3 botulinum toxin substrate 1;Ras-related C3 botulinum toxin substrate 3	RAC1;RAC3	18.2	1.0847
Q9P0K7	Ankycorbin	RAI14	4.7	1.0851
Q99623	Prohibitin-2	PHB2	7.7	1.0862
P21796	Voltage-dependent anion-selective channel protein 1	VDAC1	55.1	1.0863
P55072	Transitional endoplasmic reticulum ATPase	VCP	25.4	1.0865
Q02218	2-oxoglutarate dehydrogenase, mitochondrial	OGDH	10.9	1.0868
P32119	Peroxiredoxin-2	PRDX2	26.8	1.0870
Q92616	Translational activator GCN1	GCN1L1	2.9	1.0877
Q96TA1	Niban-like protein 1	FAM129B	14.6	1.0879
P04083	Annexin A1	ANXA1	47.7	1.0887
P98082	Disabled homolog 2	DAB2	7.3	1.0888
P62495	Eukaryotic peptide chain release factor subunit 1	ETF1	15.6	1.0896
P40121	Macrophage-capping protein	CAPG	13.2	1.0912
P50995;P27216	Annexin A11	ANXA11	11.5	1.0920
Q12797	Aspartyl/asparaginyl beta-hydroxylase	ASPH	6.3	1.0928
Q96M27	Protein PRRC1	PRRC1	14.2	1.0941
O76094	Signal recognition particle 72 kDa protein	SRP72	4.6	1.0946
Q9NYL9	Tropomodulin-3	TMOD3	27.8	1.0957
P07108	Acyl-CoA-binding protein	DBI	29.9	1.0965
P61088;Q5JXB2	Ubiquitin-conjugating enzyme E2 N	UBE2N	21.1	1.0967
P12081	Histidine--tRNA ligase, cytoplasmic	HARS	8.4	1.0968
P06576	ATP synthase subunit beta, mitochondrial	ATP5B	26.3	1.0969
Q96C90	Protein phosphatase 1 regulatory subunit 14B	PPP1R14B	30.6	1.0969
P53634	Dipeptidyl peptidase 1;Dipeptidyl peptidase 1 exclusion domain chain;Dipeptidyl peptidase 1 heavy chain;Dipeptidyl peptidase 1 light chain	CTSC	9.7	1.0970
O15511;Q9BPX5	Actin-related protein 2/3 complex subunit 5	ARPC5	20.5	1.0977
P60953;P170	Cell division control protein 42 homolog	CDC42	31.4	1.0983

81;Q9H4E5;P 15153;P8409 5				
P48735	Isocitrate dehydrogenase [NADP], mitochondrial	IDH2	6	1.1006
Q06323	Proteasome activator complex subunit 1	PSME1	28.5	1.1007
P52907	F-actin-capping protein subunit alpha-1	CAPZA1	33.2	1.1011
P08758	Annexin A5	ANXA5	30.9	1.1018
Q5JRX3	Presequence protease, mitochondrial	PITRM1	6	1.1020
Q16181;Q6Z U15	Septin-7	Sep-07	13.3	1.1023
P25705	ATP synthase subunit alpha, mitochondrial	ATP5A1	21.5	1.1026
Q9UBB4	Ataxin-10	ATXN10	5.3	1.1028
Q5SSJ5	Heterochromatin protein 1-binding protein 3	HP1BP3	6	1.1042
P67870	Casein kinase II subunit beta	CSNK2B	23.3	1.1051
P53621	Coatomer subunit alpha;Xenin;Proxenin	COPA	12.7	1.1094
P60903	Protein S100-A10	S100A10	44.3	1.1112
O43324	Eukaryotic translation elongation factor 1 epsilon-1	EEF1E1	20.7	1.1113
P12109	Collagen alpha-1(VI) chain	COL6A1	2.5	1.1116
P62937;A2B FH1;Q9Y536	Peptidyl-prolyl cis-trans isomerase A	PPIA	34.5	1.1126
Q16531	DNA damage-binding protein 1	DDB1	3.9	1.1133
Q9NR45	Sialic acid synthase	NANS	15.3	1.1134
Q6YHK3	CD109 antigen	CD109	2.4	1.1141
P12111	Collagen alpha-3(VI) chain	COL6A3	9.1	1.1144
Q9BSJ8	Extended synaptotagmin-1	ESYT1	6.2	1.1144
Q06830	Peroxiredoxin-1	PRDX1	51.3	1.1152
Q14847	LIM and SH3 domain protein 1	LASP1	22.2	1.1173
P07237	Protein disulfide-isomerase	P4HB	48.4	1.1177
O75369	Filamin-B	FLNB	23.7	1.1180
P27824	Calnexin	CANX	16.7	1.1195
P47756	F-actin-capping protein subunit beta	CAPZB	15.9	1.1203
Q14247	Src substrate cortactin	CTTN	18.5	1.1207
Q96QK1	Vacuolar protein sorting-associated protein 35	VPS35	8.2	1.1211
Q9ULC4	Malignant T-cell-amplified sequence 1	MCTS1	47.5	1.1211
Q16851	UTP--glucose-1-phosphate uridylyltransferase	UGP2	7.5	1.1237
P42224	Signal transducer and activator of transcription 1-alpha/beta	STAT1	4.9	1.1245

Q16836	Hydroxyacyl-coenzyme A dehydrogenase, mitochondrial	HADH	22.3	1.1258
P30041	Peroxiredoxin-6	PRDX6	29	1.1263
P22392;O60361	Nucleoside diphosphate kinase B;Putative nucleoside diphosphate kinase	NME2;NME2P1	34.9	1.1293
P60174	Triosephosphate isomerase	TPI1	49.7	1.1299
P45880	Voltage-dependent anion-selective channel protein 2	VDAC2	40.5	1.1300
Q9P2J5	Leucine--tRNA ligase, cytoplasmic	LARS	2.6	1.1331
P61106	Ras-related protein Rab-14	RAB14	14.4	1.1339
Q9Y305	Acyl-coenzyme A thioesterase 9, mitochondrial	ACOT9	8.9	1.1351
P52788	Spermine synthase	SMS	15.8	1.1356
O14974	Protein phosphatase 1 regulatory subunit 12A	PPP1R12A	3.8	1.1358
P50552	Vasodilator-stimulated phosphoprotein	VASP	8.9	1.1363
Q9Y6C9	Mitochondrial carrier homolog 2	MTCH2	19.1	1.1363
O43707	Alpha-actinin-4	ACTN4	44.1	1.1366
Q07954	Prolow-density lipoprotein receptor-related protein 1;Low-density lipoprotein receptor-related protein 1 85 kDa subunit;Low-density lipoprotein receptor-related protein 1 515 kDa subunit;Low-density lipoprotein receptor-related protein 1 intracellular domain	LRP1	0.9	1.1384
P63010	AP-2 complex subunit beta	AP2B1	10.8	1.1404
P04792	Heat shock protein beta-1	HSPB1	27.3	1.1407
P62158;P27482	Calmodulin;Calmodulin-like protein 3	CALM1; CALML3	14.1	1.1430
P04350	Tubulin beta-4A chain	TUBB4A	18.9	1.1432
P08473	Neprilysin	MME	6.8	1.1433
Q00610;P53675	Clathrin heavy chain 1	CLTC	27.7	1.1437
Q9UBG0	C-type mannose receptor 2	MRC2	4	1.1440
Q14011	Cold-inducible RNA-binding protein	CIRBP	20.3	1.1445
O15144	Actin-related protein 2/3 complex subunit 2	ARPC2	33	1.1450
Q00688	Peptidyl-prolyl cis-trans isomerase FKBP3	FKBP3	17.9	1.1450
Q9HCJ6	Synaptic vesicle membrane protein VAT-1 homolog-like	VAT1L	6.7	1.1451
Q13177;O75	Serine/threonine-protein kinase PAK	PAK2	8.2	1.1470

914;Q13153	2;PAK-2p27;PAK-2p34			
Q9UBS4	DnaJ homolog subfamily B member 11	DNAJB11	10.9	1.1472
P08670;P14136;P07197;Q16352;P12036	Vimentin	VIM	41.6	1.1473
P20810	Calpastatin	CAST	15.5	1.1482
P52565	Rho GDP-dissociation inhibitor 1	ARHGDIA	31.4	1.1484
Q13561	Dynactin subunit 2	DCTN2	24.4	1.1491
O43852	Calumenin	CALU	46.3	1.1499
Q99497	Protein DJ-1	PARK7	27	1.1517
P61163	Alpha-centractin	ACTR1A	28.5	1.1519
Q15019	Septin-2	Sep-02	14.1	1.1525
P31946	14-3-3 protein beta/alpha;14-3-3 protein beta/alpha, N-terminally processed	YWHAB	31.3	1.1529
P31939	Bifunctional purine biosynthesis protein PURH;Phosphoribosylaminoimidazolecarboxamide formyltransferase;IMP cyclohydrolase	ATIC	22.3	1.1534
Q9NZ08	Endoplasmic reticulum aminopeptidase 1	ERAP1	10.8	1.1540
P05120	Plasminogen activator inhibitor 2	SERPINB2	8.4	1.1541
P13473	Lysosome-associated membrane glycoprotein 2	LAMP2	4.4	1.1571
P53396	ATP-citrate synthase	ACLY	18.9	1.1588
P08133	Annexin A6	ANXA6	25.6	1.1597
P58546	Myotrophin	MTPN	44.1	1.1601
P11413	Glucose-6-phosphate 1-dehydrogenase	G6PD	17.7	1.1606
Q5VYK3	Proteasome-associated protein ECM29 homolog	ECM29	2.2	1.1645
P54920	Alpha-soluble NSF attachment protein	NAPA	13.2	1.1652
P46459	Vesicle-fusing ATPase	NSF	2.4	1.1681
Q15746	Myosin light chain kinase, smooth muscle;Myosin light chain kinase, smooth muscle, deglutamylated form	MYLK	7.7	1.1692
Q9BUF5	Tubulin beta-6 chain	TUBB6	24	1.1699
Q9Y6E0	Serine/threonine-protein kinase 24;Serine/threonine-protein kinase 24 36 kDa subunit;Serine/threonine-protein kinase 24 12 kDa subunit	STK24	4.3	1.1700

Q9NTK5	Obg-like ATPase 1	OLA1	16.2	1.1721
Q13492	Phosphatidylinositol-binding clathrin assembly protein	PICALM	12	1.1722
Q9NP97	Dynein light chain roadblock-type 1	DYNLRB1	38.5	1.1736
O95782	AP-2 complex subunit alpha-1	AP2A1	11	1.1758
P83111	Serine beta-lactamase-like protein LACTB, mitochondrial	LACTB	6.6	1.1762
P20340;Q9NRW1	Ras-related protein Rab-6A;Ras-related protein Rab-6B	RAB6A;RAB6B	16.8	1.1767
P35580	Myosin-10	MYH10	15.3	1.1783
Q9H4A4	Aminopeptidase B	RNPEP	10.2	1.1785
Q9BRA2	Thioredoxin domain-containing protein 17	TXNDC17	21.1	1.1797
O43795;Q9UBC5	Unconventional myosin-Ib	MYO1B	5.3	1.1804
O15460	Prolyl 4-hydroxylase subunit alpha-2	P4HA2	12.5	1.1822
O15145	Actin-related protein 2/3 complex subunit 3	ARPC3	25.8	1.1832
P61158;Q9P1U1	Actin-related protein 3	ACTR3	45.7	1.1844
Q04760	Lactoylglutathione lyase	GLO1	28.8	1.1894
P00338	L-lactate dehydrogenase A chain	LDHA	58.4	1.1899
Q96B97	SH3 domain-containing kinase-binding protein 1	SH3KBP1	3.9	1.1900
P46940;Q86VI3	Ras GTPase-activating-like protein IQGAP1	IQGAP1	26	1.1901
Q5T4S7	E3 ubiquitin-protein ligase UBR4	UBR4	1.2	1.1915
P35241	Radixin	RDX	13.2	1.1927
P28074	Proteasome subunit beta type-5	PSMB5	8.4	1.1928
Q04837	Single-stranded DNA-binding protein, mitochondrial	SSBP1	32.4	1.1929
P13489	Ribonuclease inhibitor	RNH1	15.6	1.1932
Q15942	Zyxin	ZYX	13.6	1.1943
P00558;P07205	Phosphoglycerate kinase 1	PGK1	47.5	1.1958
P27816	Microtubule-associated protein 4	MAP4	23.6	1.1972
P10599	Thioredoxin	TXN	55.2	1.1979
P61011	Signal recognition particle 54 kDa protein	SRP54	9.9	1.1988

P07384	Calpain-1 catalytic subunit	CAPN1	4.5	1.1994
P18206	Vinculin	VCL	25.9	1.2009
Q86UP2	Kinectin	KTN1	8	1.2012
O95336	6-phosphogluconolactonase	PGLS	17.4	1.2016
P13804	Electron transfer flavoprotein subunit alpha, mitochondrial	ETFA	12.9	1.2042
P19367	Hexokinase-1	HK1	2.7	1.2046
P47755	F-actin-capping protein subunit alpha-2	CAPZA2	31.5	1.2050
P31150	Rab GDP dissociation inhibitor alpha	GDI1	27.3	1.2055
Q9NQC3	Reticulon-4	RTN4	3.7	1.2079
P40925	Malate dehydrogenase, cytoplasmic	MDH1	18	1.2079
P40261	Nicotinamide N-methyltransferase	NNMT	8.3	1.2080
Q8NBJ7	Sulfatase-modifying factor 2	SUMF2	8.3	1.2103
P07195	L-lactate dehydrogenase B chain	LDHB	40.7	1.2106
O00429	Dynamin-1-like protein	DNM1L	10.1	1.2107
Q9Y3A5	Ribosome maturation protein SBDS	SBDS	11.2	1.2134
P04075;P09972	Fructose-bisphosphate aldolase A	ALDOA	47	1.2152
P23526	Adenosylhomocysteinase	AHCY	21.1	1.2179
P50570;Q9UQ16	Dynamin-2	DNM2	7.2	1.2188
P10515	Dihydrolipoyllysine-residue acetyltransferase component of pyruvate dehydrogenase complex, mitochondrial	DLAT	4.8	1.2195
P78417	Glutathione S-transferase omega-1	GSTO1	29	1.2196
P61160	Actin-related protein 2	ACTR2	14.7	1.2198
P37802	Transgelin-2	TAGLN2	44.7	1.2201
P61106	Ras-related protein Rab-14	RAB14	14.4	1.1339
Q9Y305	Acyl-coenzyme A thioesterase 9, mitochondrial	ACOT9	8.9	1.1351
P52788	Spermine synthase	SMS	15.8	1.1356
O14974	Protein phosphatase 1 regulatory subunit 12A	PPP1R12A	3.8	1.1358
P50552	Vasodilator-stimulated phosphoprotein	VASP	8.9	1.1363
Q9Y6C9	Mitochondrial carrier homolog 2	MTCH2	19.1	1.1363
O43707	Alpha-actinin-4	ACTN4	44.1	1.1366
Q07954	Prolow-density lipoprotein receptor-related protein 1;Low-density lipoprotein receptor-related protein	LRP1	0.9	1.1384

	1 85 kDa subunit;Low-density lipoprotein receptor-related protein 1 515 kDa subunit;Low-density lipoprotein receptor-related protein 1 intracellular domain			
P63010	AP-2 complex subunit beta	AP2B1	10.8	1.1404
P04792	Heat shock protein beta-1	HSPB1	27.3	1.1407
P62158;P27482	Calmodulin;Calmodulin-like protein 3	CALM1; CALML3	14.1	1.1430
P04350	Tubulin beta-4A chain	TUBB4A	18.9	1.1432
P08473	Neprilysin	MME	6.8	1.1433
Q00610;P53675	Clathrin heavy chain 1	CLTC	27.7	1.1437
Q9UBG0	C-type mannose receptor 2	MRC2	4	1.1440
Q14011	Cold-inducible RNA-binding protein	CIRBP	20.3	1.1445
O15144	Actin-related protein 2/3 complex subunit 2	ARPC2	33	1.1450
Q00688	Peptidyl-prolyl cis-trans isomerase FKBP3	FKBP3	17.9	1.1450
Q9HCJ6	Synaptic vesicle membrane protein VAT-1 homolog-like	VAT1L	6.7	1.1451
Q13177;O75914;Q13153	Serine/threonine-protein kinase PAK 2;PAK-2p27;PAK-2p34	PAK2	8.2	1.1470
Q9UBS4	DnaJ homolog subfamily B member 11	DNAJB11	10.9	1.1472
P08670;P14136;P07197;Q16352;P12036	Vimentin	VIM	41.6	1.1473
P20810	Calpastatin	CAST	15.5	1.1482
P52565	Rho GDP-dissociation inhibitor 1	ARHGDIA	31.4	1.1484
Q13561	Dynactin subunit 2	DCTN2	24.4	1.1491
O43852	Calumenin	CALU	46.3	1.1499
Q99497	Protein DJ-1	PARK7	27	1.1517
P61163	Alpha-centractin	ACTR1A	28.5	1.1519
Q15019	Septin-2	Sep-02	14.1	1.1525
P31946	14-3-3 protein beta/alpha;14-3-3 protein beta/alpha, N-terminally processed	YWHAB	31.3	1.1529
P31939	Bifunctional purine biosynthesis protein PURH;Phosphoribosylaminoimidazolecarboxamide formyltransferase;IMP cyclohydrolase	ATIC	22.3	1.1534

Q9NZ08	Endoplasmic reticulum aminopeptidase 1	ERAP1	10.8	1.1540
P05120	Plasminogen activator inhibitor 2	SERPINB 2	8.4	1.1541
P13473	Lysosome-associated membrane glycoprotein 2	LAMP2	4.4	1.1571
P53396	ATP-citrate synthase	ACLY	18.9	1.1588
P08133	Annexin A6	ANXA6	25.6	1.1597
P58546	Myotrophin	MTPN	44.1	1.1601
P11413	Glucose-6-phosphate 1-dehydrogenase	G6PD	17.7	1.1606
Q5VYK3	Proteasome-associated protein ECM29 homolog	ECM29	2.2	1.1645
P54920	Alpha-soluble NSF attachment protein	NAPA	13.2	1.1652
P46459	Vesicle-fusing ATPase	NSF	2.4	1.1681
Q15746	Myosin light chain kinase, smooth muscle;Myosin light chain kinase, smooth muscle, deglutamylated form	MYLK	7.7	1.1692
Q9BUF5	Tubulin beta-6 chain	TUBB6	24	1.1699
Q9Y6E0	Serine/threonine-protein kinase 24;Serine/threonine-protein kinase 24 36 kDa subunit;Serine/threonine-protein kinase 24 12 kDa subunit	STK24	4.3	1.1700
Q9NTK5	Obg-like ATPase 1	OLA1	16.2	1.1721
Q13492	Phosphatidylinositol-binding clathrin assembly protein	PICALM	12	1.1722
Q9NP97	Dynein light chain roadblock-type 1	DYNLRB 1	38.5	1.1736
O95782	AP-2 complex subunit alpha-1	AP2A1	11	1.1758
P83111	Serine beta-lactamase-like protein LACTB, mitochondrial	LACTB	6.6	1.1762
P20340;Q9NRW1	Ras-related protein Rab-6A;Ras-related protein Rab-6B	RAB6A;RAB6B	16.8	1.1767
P35580	Myosin-10	MYH10	15.3	1.1783
Q9H4A4	Aminopeptidase B	RNPEP	10.2	1.1785
Q9BRA2	Thioredoxin domain-containing protein 17	TXNDC17	21.1	1.1797
O43795;Q9UBC5	Unconventional myosin-Ib	MYO1B	5.3	1.1804
O15460	Prolyl 4-hydroxylase subunit alpha-2	P4HA2	12.5	1.1822
O15145	Actin-related protein 2/3 complex subunit 3	ARPC3	25.8	1.1832
P61158;Q9P1	Actin-related protein 3	ACTR3	45.7	1.1844

U1				
Q04760	Lactoylglutathione lyase	GLO1	28.8	1.1894
P00338	L-lactate dehydrogenase A chain	LDHA	58.4	1.1899
Q96B97	SH3 domain-containing kinase-binding protein 1	SH3KBP1	3.9	1.1900
P46940;Q86VI3	Ras GTPase-activating-like protein IQGAP1	IQGAP1	26	1.1901
Q5T4S7	E3 ubiquitin-protein ligase UBR4	UBR4	1.2	1.1915
P35241	Radixin	RDX	13.2	1.1927
P28074	Proteasome subunit beta type-5	PSMB5	8.4	1.1928
Q04837	Single-stranded DNA-binding protein, mitochondrial	SSBP1	32.4	1.1929
P13489	Ribonuclease inhibitor	RNH1	15.6	1.1932
Q15942	Zyxin	ZYX	13.6	1.1943
P00558;P07205	Phosphoglycerate kinase 1	PGK1	47.5	1.1958
P27816	Microtubule-associated protein 4	MAP4	23.6	1.1972
P10599	Thioredoxin	TXN	55.2	1.1979
P61011	Signal recognition particle 54 kDa protein	SRP54	9.9	1.1988
P07384	Calpain-1 catalytic subunit	CAPN1	4.5	1.1994
P18206	Vinculin	VCL	25.9	1.2009
Q86UP2	Kinectin	KTN1	8	1.2012
O95336	6-phosphogluconolactonase	PGLS	17.4	1.2016
P13804	Electron transfer flavoprotein subunit alpha, mitochondrial	ETFA	12.9	1.2042
P19367	Hexokinase-1	HK1	2.7	1.2046
P47755	F-actin-capping protein subunit alpha-2	CAPZA2	31.5	1.2050
P31150	Rab GDP dissociation inhibitor alpha	GDI1	27.3	1.2055
Q9NQC3	Reticulon-4	RTN4	3.7	1.2079
P40925	Malate dehydrogenase, cytoplasmic	MDH1	18	1.2079
P40261	Nicotinamide N-methyltransferase	NNMT	8.3	1.2080
Q8NBJ7	Sulfatase-modifying factor 2	SUMF2	8.3	1.2103
P07195	L-lactate dehydrogenase B chain	LDHB	40.7	1.2106
O00429	Dynamin-1-like protein	DNM1L	10.1	1.2107
Q9Y3A5	Ribosome maturation protein SBDS	SBDS	11.2	1.2134
P04075;P09972	Fructose-bisphosphate aldolase A	ALDOA	47	1.2152
P23526	Adenosylhomocysteinase	AHCY	21.1	1.2179

P50570;Q9UQ16	Dynamin-2	DNM2	7.2	1.2188
P10515	Dihydrolipoyllysine-residue acetyltransferase component of pyruvate dehydrogenase complex, mitochondrial	DLAT	4.8	1.2195
P78417	Glutathione S-transferase omega-1	GSTO1	29	1.2196
P61160	Actin-related protein 2	ACTR2	14.7	1.2198
P37802	Transgelin-2	TAGLN2	44.7	1.2201
Q9NZM1;O75923	Myoferlin	MYOF	12.8	1.3216
Q13509	Tubulin beta-3 chain	TUBB3	27.3	1.3229
P18669;Q8N0Y7;P15259	Phosphoglycerate mutase 1;Probable phosphoglycerate mutase 4;Phosphoglycerate mutase 2	PGAM1;PGAM4;PGAM2	35	1.3231
Q9GZZ9	Ubiquitin-like modifier-activating enzyme 5	UBA5	12.6	1.3261
Q15363	Transmembrane emp24 domain-containing protein 2	TMED2	19.9	1.3266
O14950;P19105;P24844	Myosin regulatory light chain 12B;Myosin regulatory light chain 12A;Myosin regulatory light polypeptide 9	MYL12B;MYL12A;MYL9	32.6	1.3303
Q9NPH2	Inositol-3-phosphate synthase 1	ISYNA1	7.3	1.3337
P09525	Annexin A4	ANXA4	14.1	1.3349
P00441	Superoxide dismutase [Cu-Zn]	SOD1	32.5	1.3363
Q14192	Four and a half LIM domains protein 2	FHL2	26.2	1.3366
Q92783	Signal transducing adapter molecule 1	STAM	8	1.3395
P00491	Purine nucleoside phosphorylase	PNP	20.8	1.3424
P61970	Nuclear transport factor 2	NUTF2	45.7	1.3455
P55786;A6NEC2	Puromycin-sensitive aminopeptidase	NPEPPS	14.7	1.3473
P46926;Q8TDQ7	Glucosamine-6-phosphate isomerase 1;Glucosamine-6-phosphate isomerase 2	GNPDA1;GNPDA2	9	1.3475
P43490	Nicotinamide phosphoribosyltransferase	NAMPT	27.1	1.3492
P55263	Adenosine kinase	ADK	15.5	1.3515
P11047	Laminin subunit gamma-1	LAMC1	3.9	1.3528
P12814;Q08043;P35609	Alpha-actinin-1	ACTN1	38.3	1.3528
P35579;P35749;A7E2Y1;	Myosin-9	MYH9	36.5	1.3538

Q9UKX2;P11055;P12882;P13533;Q9Y623;Q9UKX3;P13535;P12883;REV__Q9UKV3				
P38606	V-type proton ATPase catalytic subunit A	ATP6V1A	11.5	1.3589
O95394	Phosphoacetylglucosamine mutase	PGM3	3.9	1.3589
P21291	Cysteine and glycine-rich protein 1	CSRP1	64.2	1.3667
Q9P000	COMM domain-containing protein 9	COMMD9	18.7	1.3721
P21964	Catechol O-methyltransferase	COMT	26.2	1.3726
P14550	Alcohol dehydrogenase [NADP(+)]	AKR1A1	28.9	1.3739
Q8NE71	ATP-binding cassette sub-family F member 1	ABCF1	3.2	1.3783
P51572	B-cell receptor-associated protein 31	BCAP31	12.2	1.3786
P50479	PDZ and LIM domain protein 4	PDLIM4	17.3	1.3828
Q15121	Astrocytic phosphoprotein PEA-15	PEA15	16.9	1.3875
P10768	S-formylglutathione hydrolase	ESD	17.7	1.3924
Q04446	1,4-alpha-glucan-branching enzyme	GBE1	16.1	1.3947
Q8WUP2	Filamin-binding LIM protein 1	FBLIM1	8.6	1.3966
P59998	Actin-related protein 2/3 complex subunit 4	ARPC4	13.1	1.3977
P49189	4-trimethylaminobutyraldehyde dehydrogenase	ALDH9A1	11.1	1.4008
P27105	Erythrocyte band 7 integral membrane protein	STOM	16.7	1.4031
P30086	Phosphatidylethanolamine-binding protein 1;Hippocampal cholinergic neurostimulating peptide	PEBP1	41.7	1.4035
P17301	Integrin alpha-2	ITGA2	13.4	1.4101
P31949	Protein S100-A11	S100A11	27.6	1.4128
P17655	Calpain-2 catalytic subunit	CAPN2	27.9	1.4181
P04080	Cystatin-B	CSTB	23.5	1.4212
Q9NQ88	Probable fructose-2,6-bisphosphatase TIGAR	TIGAR	13	1.4226
P14625;Q58FF3	Endoplasmin	HSP90B1	30	1.4263
P51659	Peroxisomal multifunctional enzyme type 2;(3R)-hydroxyacyl-CoA	HSD17B4	7.9	1.4309

	dehydrogenase;Enoyl-CoA hydratase 2			
Q9BWD1	Acetyl-CoA acetyltransferase, cytosolic	ACAT2	19.9	1.4313
O60664	Perilipin-3	PLIN3	37.3	1.4407
P04632;Q96L46	Calpain small subunit 1	CAPNS1	11.6	1.4411
Q9H2U2	Inorganic pyrophosphatase 2, mitochondrial	PPA2	12.9	1.4457
O43504	Hepatitis B virus X-interacting protein	HBXIP	37.4	1.4476
O75874	Isocitrate dehydrogenase [NADP] cytoplasmic	IDH1	31.6	1.4481
P52306	Rap1 GTPase-GDP dissociation stimulator 1	RAP1GDS1	11	1.4549
O00151	PDZ and LIM domain protein 1	PDLIM1	27.4	1.4622
Q14108	Lysosome membrane protein 2	SCARB2	10.3	1.4655
Q96HE7	ERO1-like protein alpha	ERO1L	2.4	1.4665
P13667	Protein disulfide-isomerase A4	PDIA4	29.1	1.4673
Q6NZI2	Polymerase I and transcript release factor	PTRF	11.5	1.4724
Q7Z3E5	LisH domain-containing protein ARMC9	ARMC9	4.4	1.4727
P21266	Glutathione S-transferase Mu 3	GSTM3	28.4	1.4796
P04062	Glucosylceramidase	GBA	9.3	1.4857
P52209	6-phosphogluconate dehydrogenase, decarboxylating	PGD	22.2	1.4870
P09936	Ubiquitin carboxyl-terminal hydrolase isozyme L1	UCHL1	32.3	1.4911
P23381	Tryptophan--tRNA ligase, cytoplasmic;T1-TrpRS;T2-TrpRS	WARS	26.8	1.4914
Q96IZ0	PRKC apoptosis WT1 regulator protein	PAWR	11.8	1.4920
Q9Y4I1;Q9ULV0	Unconventional myosin-Va;Unconventional myosin-Vb	MYO5A;MYO5B	1.5	1.4930
P14868	Aspartate--tRNA ligase, cytoplasmic	DARS	5.8	1.4931
Q13045	Protein flightless-1 homolog	FLII	2.1	1.4999
Q53GQ0	Estradiol 17-beta-dehydrogenase 12	HSD17B12	20.8	1.5002
Q12765	Secernin-1	SCRN1	4.1	1.5028
Q13557;Q13554;Q13555;Q9UQM7	Calcium/calmodulin-dependent protein kinase type II subunit delta	CAMK2D	15	1.5088
Q99536	Synaptic vesicle membrane protein VAT-1 homolog	VAT1	14	1.5142
Q07960	Rho GTPase-activating protein 1	ARHGAP	21.6	1.5174

		1		
P17931	Galectin-3	LGALS3	8.4	1.5285
Q13885;Q9BVA1;A6NNZ2	Tubulin beta-2A chain;Tubulin beta-2B chain	TUBB2A; TUBB2B	17.8	1.5298
O94973	AP-2 complex subunit alpha-2	AP2A2	11	1.5300
O00154	Cytosolic acyl coenzyme A thioester hydrolase	ACOT7	7.1	1.5316
Q6UXH1	Cysteine-rich with EGF-like domain protein 2	CRELD2	8.2	1.5329
P26447	Protein S100-A4	S100A4	27.7	1.5336
P02751	Fibronectin;Anastellin;Ugl-Y1;Ugl-Y2;Ugl-Y3	FN1	9.3	1.5361
P07339	Cathepsin D;Cathepsin D light chain;Cathepsin D heavy chain	CTSD	24.5	1.5369
P55084	Trifunctional enzyme subunit beta, mitochondrial;3-ketoacyl-CoA thiolase	HADHB	22.4	1.5452
P07942	Laminin subunit beta-1	LAMB1	5.5	1.5466
O75368	SH3 domain-binding glutamic acid-rich-like protein	SH3BGRL	38.6	1.5578
P40939	Trifunctional enzyme subunit alpha, mitochondrial;Long-chain enoyl-CoA hydratase;Long chain 3-hydroxyacyl-CoA dehydrogenase	HADHA	24.2	1.5612
P09104	Gamma-enolase	ENO2	14.5	1.5723
P39019	40S ribosomal protein S19	RPS19	20.7	1.5794
P09960	Leukotriene A-4 hydrolase	LTA4H	8.3	1.5918
Q969G5	Protein kinase C delta-binding protein	PRKCDBP	5.7	1.5985
Q9NRV9	Heme-binding protein 1	HEBP1	22.2	1.6045
P30044	Peroxiredoxin-5, mitochondrial	PRDX5	30.4	1.6093
P21399	Cytoplasmic aconitate hydratase	ACO1	17	1.6116
O00469	Procollagen-lysine,2-oxoglutarate 5-dioxygenase 2	PLOD2	14.2	1.6305
O95747	Serine/threonine-protein kinase OSR1	OXSR1	6.5	1.6339
Q9UJU6	Drebrin-like protein	DBNL	11.4	1.6405
P02462	Collagen alpha-1(IV) chain;Arresten	COL4A1	1.8	1.6471
P15144	Aminopeptidase N	ANPEP	12.4	1.6497
P53007	Tricarboxylate transport protein, mitochondrial	SLC25A1	11.3	1.6517
Q03135	Caveolin-1	CAV1	21.3	1.6536
P07858	Cathepsin B;Cathepsin B light chain;Cathepsin B	CTSB	14.7	1.6671

	heavy chain			
Q9Y4K0	Lysyl oxidase homolog 2	LOXL2	7.8	1.6704
P07686	Beta-hexosaminidase subunit beta;Beta-hexosaminidase subunit beta chain B;Beta-hexosaminidase subunit beta chain A	HEXB	15.1	1.6717
P30740;O75830	Leukocyte elastase inhibitor;Serpin I2	SERPINB1;SERPINI2	6.3	1.7331
P17174	Aspartate aminotransferase, cytoplasmic	GOT1	23	1.7818
Q92626	Peroxidasin homolog	PXDN	2.9	1.8152
Q08380	Galectin-3-binding protein	LGALS3BP	7	1.8612
P06703	Protein S100-A6	S100A6	55.6	1.8739
P36542	ATP synthase subunit gamma, mitochondrial	ATP5C1	7.4	1.9149
P07602	Proactivator polypeptide;Saposin-A;Saposin-B-Val;Saposin-B;Saposin-C;Saposin-D	PSAP	20	1.9302
Q9UIJ7	GTP:AMP phosphotransferase, mitochondrial	AK3	17.2	1.9706
Q9NRX4	14 kDa phosphohistidine phosphatase	PHPT1	16	1.9820
Q9Y6G9	Cytoplasmic dynein 1 light intermediate chain 1	DYNC1LI1	12.6	1.9919
Q9UKK9	ADP-sugar pyrophosphatase	NUDT5	15.1	1.9958
P11216	Glycogen phosphorylase, brain form	PYGB	9.7	1.9971
Q99685	Monoglyceride lipase	MGLL	10.2	2.0111
Q9NZN3	EH domain-containing protein 3	EHD3	11.8	2.0336
Q13459	Unconventional myosin-IXb	MYO9B	1.1	2.1214
P45974;Q92995	Ubiquitin carboxyl-terminal hydrolase 5	USP5	4.4	2.1350
P35754	Glutaredoxin-1	GLRX	19.8	2.2356
P06396;CON__Q3SX14	Gelsolin	GSN	26	2.2624
P08572	Collagen alpha-2(IV) chain;Canstatin	COL4A2	1.5	2.3270
Q13510	Acid ceramidase;Acid ceramidase subunit alpha;Acid ceramidase subunit beta	ASAH1	10.1	2.3585
Q9BZQ8	Protein Niban	FAM129A	4.6	2.4643
P51911	Calponin-1	CNN1	35.4	2.4823
P37235;P840	Hippocalcin-like protein 1;Neuron-specific	HPCAL1;	18.1	2.5190

74;P61601	calcium-binding protein hippocalcin	HPCA		
Q9BRX8	Redox-regulatory protein FAM213A	FAM213 A	10	2.8571
Q13620	Cullin-4B	CUL4B	3.2	3.0376
Q9BRF8	Calcineurin-like phosphoesterase domain-containing protein 1	CPPED1	10.5	3.0384
P15121;C9JR Z8	Aldose reductase	AKR1B1	50.9	3.0747
Q9UMS6	Synaptopodin-2	SYNPO2	1.6	3.1172
P42166;P421 67	Lamina-associated polypeptide 2, isoform alpha;Thymopoietin;Thymopentin;Lamina-associat ed polypeptide 2, isoforms beta/gamma;Thymopoietin;Thymopentin	TMPO	3.7	4.4263
